# Pd-Catalyzed Aromatic Dual C-H Acylations and Intramolecular Cyclization: Access to Quinoline-Substituted Hydroxyl Isoindolones

**DOI:** 10.3390/molecules29225397

**Published:** 2024-11-15

**Authors:** Hongke Xu, Yuchen Yang, Fei Li, Yuzhu Yang

**Affiliations:** 1 State Key Laboratory of Functions and Applications of Medicinal Plants, Guizhou Medical University, 3491 Gaohai Road, Guiyang 550014, China; 2 Natural Products Research Center of Guizhou Province, 3491 Baijin Road, Guiyang 550014, China

**Keywords:** palladium catalysis, C-H acylation, cyclization, bidentate directed system, hydroxyl isoindolones

## Abstract

A palladium-catalyzed aromatic dual C-H acylations followed with intramolecular cyclizations have been developed by the assistance of bidentate *N*-(quinolin-8-yl)benzamide. This tandem process involves the formation of three new chemical bonds, providing access to novel quinoline-substituted hydroxyl isoindolones skeleton under simple reaction conditions. The deuterium-labeled competition reaction has revealed that C-H bond cleavage is the turnover limiting step.

## 1. Introduction

**Scheme 1:**
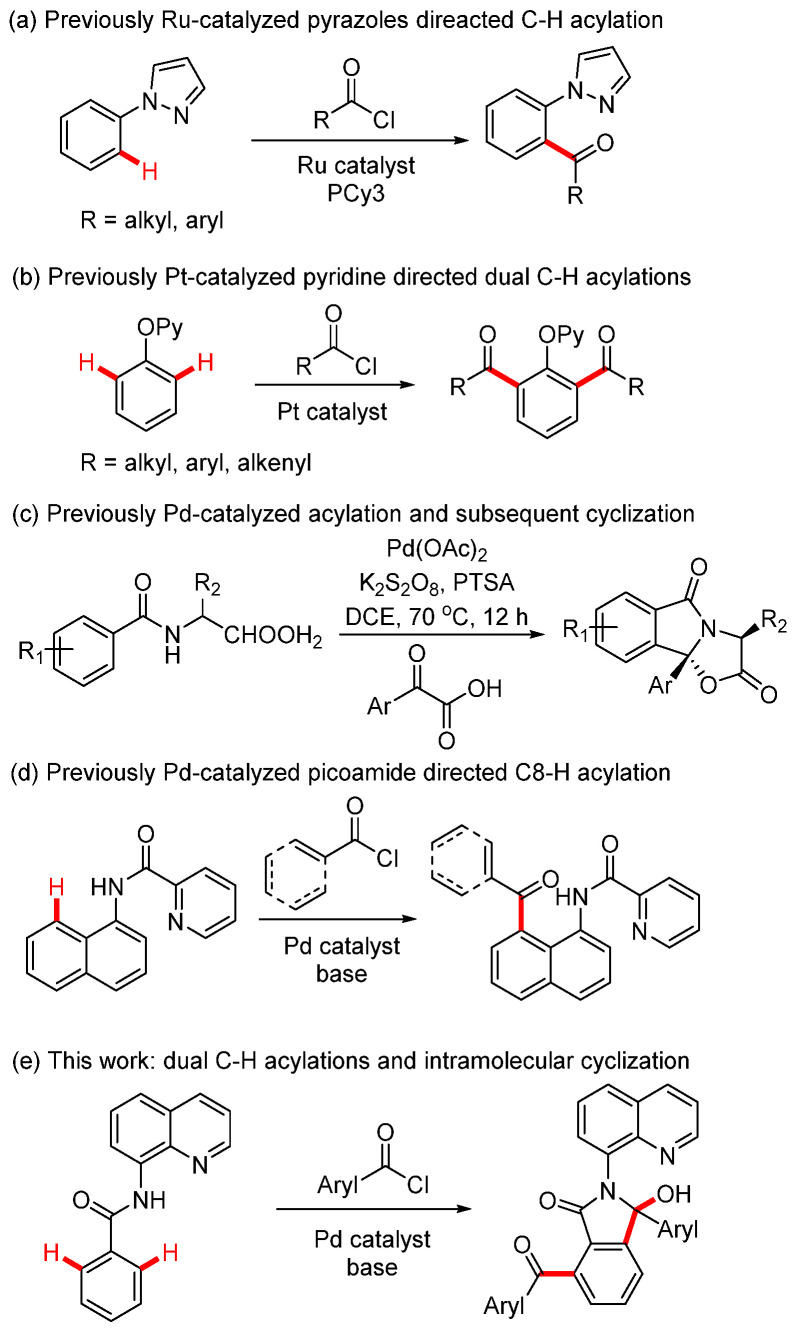
Metal-catalyzed C-H acylations from acyl chlorides.

Transition metal-catalyzed C-H acylation reactions have been intensively investigated as the ketone products serve as valuable moieties in natural products, functional materials, and drug discovery [1]. Several reagents have been developed in catalytic C-H acylations, including aldehyde [2], α-oxocarboxylic acids [3], alcohol [4], aryl methanes [5], cyclopropenones [6], anhydrides [7], ketenes [8], and acyl fluorides [9]. Acyl chlorides are less commonly used in C-H acylations since they are typically employed in Friedel–Crafts acylation under Lewis acid catalysis [10]. However, utilizing acyl chlorides in C-H acylations presents a promising strategy, as only a base is required for the cleavage of hydrogen chloride and no external oxidant is needed for substrate or metal catalyst turnover. To the best of our knowledge, very few reactions have described the C-H acylations with acyl chlorides assisted by directing group strategy. In 2013, the Frost group reported the Ru-catalyzed aromatic C-H acylation of arylpyrazoles, demonstrating good functional group tolerance of both aryl and alkyl acyl chlorides (Scheme 1a) [11]. In 2017, the Huo group reported Pt-catalyzed dual C-H acylation of 2-(aryloxy)pyridines, yielding diacylated products (Scheme 1b) [12]. In 2019, Wang and coworkers reported Pd-catalyzed ortho-acylation of *N*-benzoyl α-amino acid derivatives and subsequent intramolecular cyclizations for the synthesis of oxazoloisoindolinones (Scheme 1c) [13]. Later, Wu’s group described palladium-catalyzed C8-H acylation of 1-Naphthylamines with acyl chlorides (Scheme 1d) [14].

**Figure 1:**
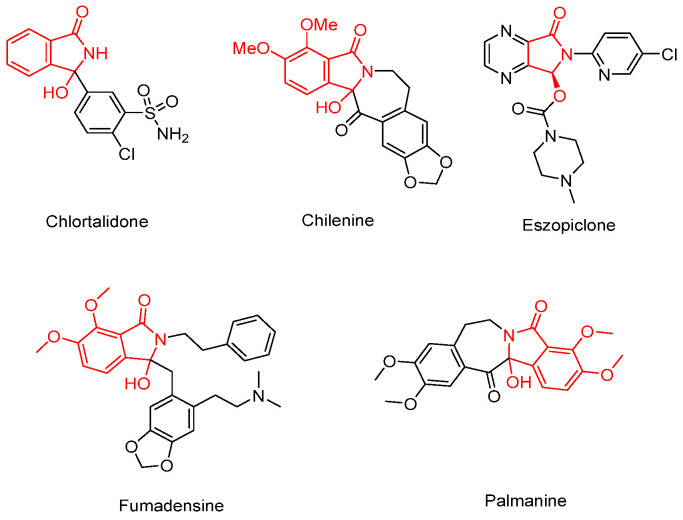
Bioactive compounds with a hydroxyl isoindolone skeleton.

Hydroxyl isoindolones represent a useful class of heterocycles that exist in many natural products and drugs, such as chlortalidone, chilenine, eszopicloneand, palmanine, and fumadensine (Figure 1) [15,16,17,18,19]. Therefore, the diversity of synthetic methods for the preparation of the hydroxyl isoindolone core structure has attracted the attention of organic chemists, who continue to make efforts in this area. Herein, we wish to present our findings on Pd-catalyzed dual acylation with acyl chlorides and intramolecular cyclization, facilitated by *N*-(quinolin-8-yl)benzamide-directed ortho C-H activation, resulting in novel quinoline-substituted hydroxyl isoindolones (Scheme 1e).

## 2. Results and Discussion

**Scheme 2:**
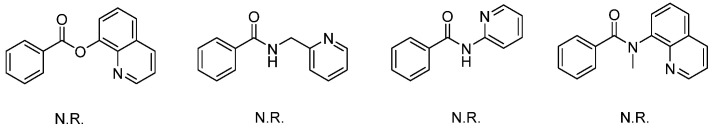
Screening of other bidentate-directing substrates.

We initiated the optimization process using *N*-(quinolin-8-yl)benzamide and benzoyl chloride as model substrates. The reaction yielded an unexpected product **3aa** in 39% yield under Pd(OAc)_2_ catalysis, utilizing KOAc as the base and toluene as the solvent (Table 1, entry 1). The structure of **3aa** was determined through NMR and HRMS analysis and was fully confirmed by X-ray diffraction (CDCC 2371075). The product was generated via dual acylations and intramolecular cyclization [20,21]. The subsequent optimization involved screening various metal catalysts, bases, solvents, and concentrations to enhance the yield. Different bases were tested in this reaction, and the results indicated that NaOAc was the optimal choice (entry 4). In addition to Pd(OAc)_2_, other palladium(II) catalysts, such as PdCl_2_(MeCN)_2_, PdCl_2_, Pd_2_(dba)_3_, and Pd(TFA)_2_, were also compatible in this tandem reaction, although they produced the corresponding product in lower yields (entries 9–12). The solvent screening included both polar and non-polar solvents (entries 13–20), and the highest yield was obtained using xylene as the solvent (entry 18). Subsequently, the reaction conditions, including concentration, temperature, and duration, were optimized (entries 21–25), resulting in a maximum yield of 83% with 2 mL of xylene at 90 °C for 15 h (entry 22). With lower Pd catalyst loading, the yield of the product decreased to 67% (5 mol% Pd(OAc)_2_) and 60% (2.5 mol% Pd(OAc)_2_). The product was isolated in 68% yield when the reaction was performed under air (entry 28). When benzoyl bromide was employed as the acylation reagent, the yield of the product decreased to 42% (entry 29). No reaction occurred in the absence of the palladium catalyst or base (entries 30–31). It is noteworthy that aside from the starting materials and product **3aa**, no other products or intermediates were detected by thin-layer chromatography (TLC) or isolated from chromatography during the optimization process. In addition to *N*-(quinolin-8-yl)benzamide as the directing group, other bidentate substrates were also tested in this reaction system; however, the results indicated that they were not promising for this transformation (Scheme 2).

**Scheme 3:**
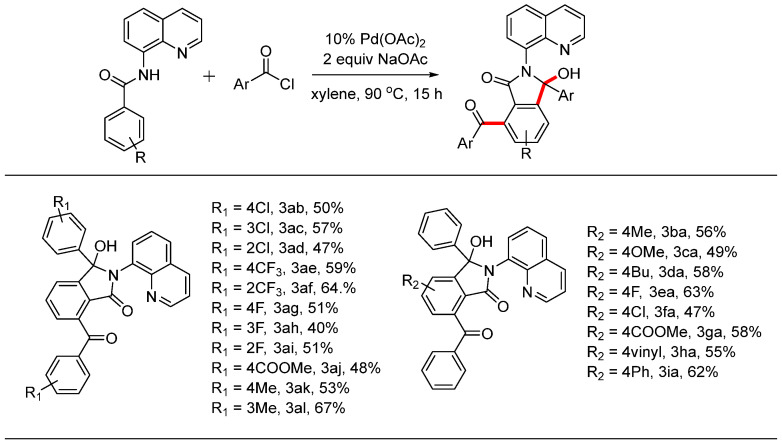
Pd-catalyzed dual C-H aroylations and cyclization reactions.

With the optimal reaction conditions established, we investigated the substrate scope using various substituted *N*-(quinolin-8-yl)benzamide derivatives, and the results are summarized in Scheme 3. The reaction conditions proved compatible with aromatic acyl chloride derivatives containing functional groups such as -F, -Cl, -CF_3_, -COOMe, and -Me, yielding the desired products in moderate yields. When comparing substituents in different positions on the phenyl ring, the steric effect did not significantly impact the yield of the product. For instance, the yield of product **3af** (64%) with ortho-substitution was higher than that of the para-substituted product **3ae** (59%). Similar results were observed for products with chloride groups (**3ab**, **3ac**, and **3ad**), fluorine groups (**3ag**, **3ah**, and **3ai**), and methyl groups (**3ak** and **3al**). Next, we also examined the scope of *N*-(quinolin-8-yl)benzamide derivatives. The introduction of various groups, including both electron-donating (-Me, -OMe, and -*^n^*Bu) and electron-withdrawing (-F, -Cl, and -COOMe) substituents, was compatible with the reaction conditions, resulting in the desired products in moderate yields. Additionally, vinyl and phenyl groups at the para position successfully underwent this reaction transformation, yielding the corresponding products **3ha** and **3ia** in 55% and 62%, respectively.

**Scheme 4:**
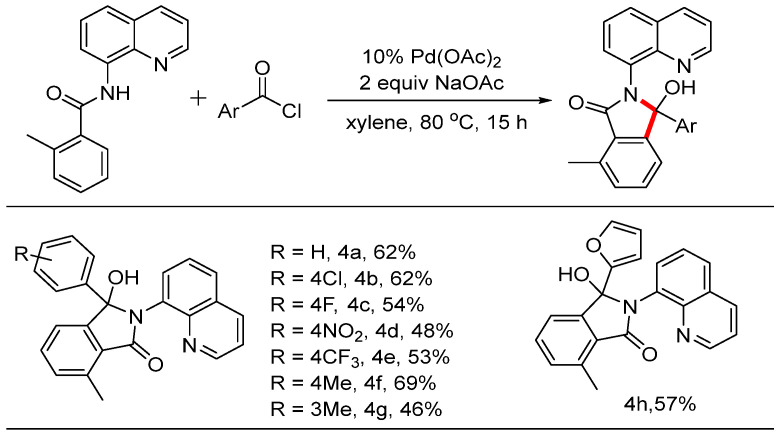
Pd-catalyzed mono C-H aroylation and cyclization reactions.

**Scheme 5:**
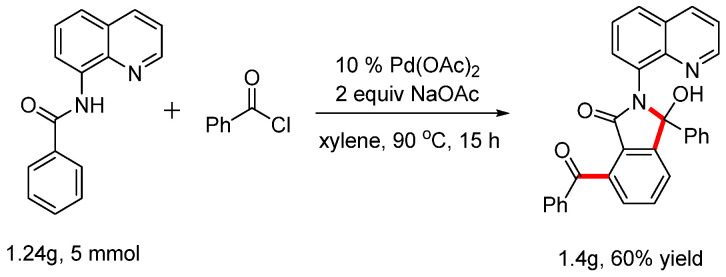
Gram-scale reaction.

When the ortho position of the phenyl ring was occupied by a methyl group, the reaction yielded only mono-acylation and cyclization products. The scope of this reaction was also investigated, and the results are summarized in Scheme 4. When benzoyl chloride was subjected to these reaction conditions, product **4a** was isolated with a yield of 62%. The structure was determined by NMR and HRMS and was fully confirmed by X-ray diffraction analysis (CDCC 2371076). Electron-withdrawing groups such as -Cl, -F, -NO_2_, and -CF_3_ at the para position were tolerated in this system, resulting in products with moderate yields. To observe the steric effect, the methyl group at the para position produced product **4f** with a yield of 69%, while the meta-substituted substrate yielded product **4g**, also of 69%. Notably, when 2-furoyl chloride was used in this reaction, the desired product **4h** was isolated with a yield of 57%. To test whether the reaction could be scaled up to generate preparatively useful quantities of material (Scheme 5), we attempted a gram-scale reaction using 1.24 g (5 mmol) of starting materials. Gratifyingly, this was converted into the product 1.40 g with a yield of 60%.

**Scheme 6:**
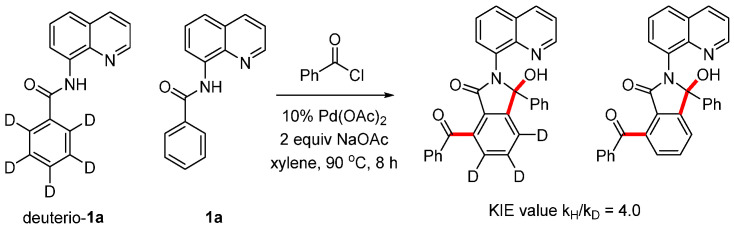
Kinetic isotope effect experiment.

To investigate the catalytic mechanism, we conducted a competition experiment using equimolar amounts of deuterio-**1a** and *N*-(quinolin-8-yl)benzamide **1a** with benzoyl chloride under our standard conditions for 8 h. This resulted in an intermolecular kinetic isotope effect (kH/kD) of 4.0. These results indicate that C-H activation is the rate-determining step of this reaction (Scheme 6).

**Scheme 7:**
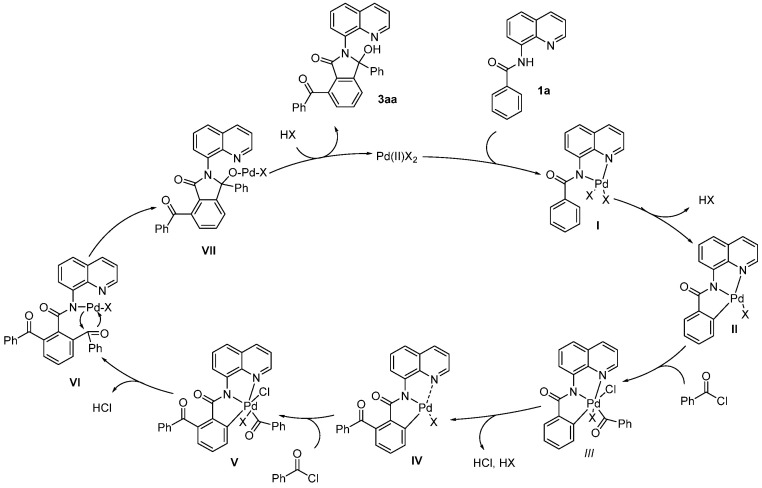
Proposed mechanism.

Based on the experimental results and previous literature [13,14], a plausible mechanism is proposed in Scheme 7. First, palladium intermediate I is generated by the coordination of two nitrogen atoms from the amide and quinoline of substrate 1a to the Pd catalyst, followed by a concerted metalation–deprotonation process that produces intermediate II. Next, the oxidative addition of acyl chlorides to II leads to the formation of intermediate III, which undergoes reductive elimination to yield the mono-acylated Pd intermediate IV. A similar oxidative addition and reductive elimination occur to generate Pd intermediates V and VI, in which the Pd atom coordinates to the nitrogen atom of benzamide. The intramolecular cyclization of VI produces intermediate VII, which is followed by protonation to yield the final product **3aa**, along with the simultaneous release of a Pd(II) species to complete the catalytic cycle.

## 3. Materials and Methods

### 3.1. General Information

^1^H NMR and ^13^C NMR were recorded in CDCl_3_ or DMSO-d6 at room temperature on the Bruker AVANCE NEO 600 (151 MHz for 13C NMR), Billerica, MA, USA. The chemical shifts scale is based on internal TMS. The peak patterns are indicated as follows: s, singlet; d, doublet; t, triplet; q, quartet; m, multiplet; qui, quintet; sxt, sextet. The coupling constants, J, are reported in Hertz (Hz). Mass spectroscopy data were collected on an HRMS-ESI instrument. Unless otherwise noted, all reagents were obtained from commercial suppliers and used without further purification. All solvents were purified and dried according to standard methods prior to use. Products were purified by flash column chromatography on 100–200 mesh silica gel, SiO_2_.

### 3.2. Typical Procedure for the Preparation of Benzamides

All benzamides **1** were synthesized from the corresponding benzoic acids or benzoyl chlorides and 8-aminoquinoline. The deuterated amides were synthesized according to a literature method, and the spectral properties are consistent with the literature values [22,23,24,25,26]. The following amides were synthesized according to the literature procedures [27].

### 3.3. General Procedure for the Synthesis of Compound ***3***

A Schlenk tube was equipped with a magnetic stir bar and charged with substituted *N*-(quinolin-8-yl)benzamide **1** (0.1 mmol), **2** (0.25 mmol), NaOAc (0.2 mmol, 16 mg), Pd(OAc)_2_ (0.01 mmol, 2.3 mg), and xylene (2 mL). Then, the flask was sealed under N_2_ and stirred at 90 °C for 16 h. After the reaction was quenched by the addition of water, the mixture was extracted with dichloromethane and the combined organic layer was dried over sodium sulfate. The concentration in vacuo followed by silica gel column purification with petroleum ether/ethyl acetate eluent gave the desired product **3**.

*7-Benzoyl-3-hydroxy-3-phenyl-2-(quinolin-8-yl)isoindolin-1-one* (**3aa**, new compound): Following the general procedure, the title compound was isolated by flash chromatography (eluent: petrol ether/ethyl acetate = 2/1~1/1) as a white solid in 83% yield (38 mg), mp 115–116 °C. ^1^H NMR (600 MHz, CDCl_3_) δ 10.72 (s, 1H), 8.82 (dd, *J* = 4.4, 1.7 Hz, 1H), 8.19 (dd, *J* = 8.3, 1.7 Hz, 1H), 7.93–7.89 (m, 2H), 7.70 (t, *J* = 7.6 Hz, 1H), 7.64 (dd, *J* = 8.2, 1.4 Hz, 1H), 7.60 (dd, *J* = 7.6, 1.4 Hz, 1H), 7.55–7.48 (m, 3H), 7.47–7.38 (m, 6H), 7.18–7.11 (m, 3H). ^13^C NMR (151 MHz, CDCl_3_) δ 196.6, 167.2, 150.7, 148.4, 143.6, 139.4, 138.1, 137.9, 137.7, 137.7, 137.1, 134.3, 133.6, 133.2, 132.0, 129.8, 129.4, 128.8, 128.2, 127.9, 127.5, 127.3, 127.2, 127.1, 126.9, 126.8, 124.0, 123.7, 121.2, 93.3. HRMS(ESI) *m*/*z* [M + H]^+^ Calcd for C_30_H_20_N_2_O_3_: 457.5010, found: 457.5086.

*7-(4-Chlorobenzoyl)-3-(4-chlorophenyl)-3-hydroxy-2-(quinolin-8-yl)isoindolin-1-one* (**3ab**, new compound): Following the general procedure, the title compound was isolated by flash chromatography (eluent: petrol ether/ethyl acetate = 2/1~1/1) as a white solid in 50% yield (26.25 mg), mp 125–128 °C. ^1^H NMR (600 MHz, CDCl_3_) δ 10.87 (s, 1H), 8.82 (dd, *J* = 4.4, 1.7 Hz, 1H), 8.22 (d, *J* = 8.2 Hz, 1H), 7.86–7.81 (m, 2H), 7.73 (d, *J* = 7.6 Hz, 1H), 7.71–7.67 (m, 1H), 7.59 (d, *J* = 7.5 Hz, 1H), 7.55 (dd, *J* = 7.5, 0.9 Hz, 1H), 7.50–7.44 (m, 3H), 7.39–7.37 (m, 2H), 7.35–7.32 (m, 2H), 7.16–7.11 (m, 2H). ^13^C NMR (151 MHz, CDCl_3_) δ 195.0, 167.0, 150.2, 148.5, 139.9, 138.2, 137.1, 135.5, 134.1, 133.6, 130.9, 129.5, 128.8, 128.4, 128.1, 128.0, 127.6, 124.3, 121.4, 92.9. HRMS(ESI) *m*/*z* [M + H]^+^ Calcd for C_30_H_18_Cl_2_N_2_O_3_: 525.0694, found: 525.0770.

*7-(3-Chlorobenzoyl)-3-(3-chlorophenyl)-3-hydroxy-2-(quinolin-8-yl)isoindolin-1-one* (**3ac**, new compound): Following the general procedure, the title compound was isolated by flash chromatography (eluent: petrol ether/ethyl acetate = 2/1~1/1) as a white solid in 57% yield (30 mg), mp 120–123 °C. ^1^H NMR (600 MHz, CDCl_3_) δ 10.61 (s, 1H), 8.80 (d, *J* = 4.4 Hz, 1H), 8.16 (d, *J* = 8.3 Hz, 1H), 7.90 (s, 1H), 7.76–7.70 (m, 2H), 7.65 (d, *J* = 8.2 Hz, 1H), 7.59–7.42 (m, 7H), 7.33 (d, *J* = 8.0 Hz, 1H), 7.25–7.21 (m, 1H), 7.11 (d, *J* = 5.0 Hz, 2H). ^13^C NMR (151 MHz, CDCl_3_) δ 195.2, 167.3, 150.6, 149.1, 143.9, 142.2, 139.1, 138.5, 137.2, 135.1, 134.6, 134.0, 133.7, 133.4, 132.3, 130.1, 129.9, 129.8, 129.6, 128.8, 128.4, 128.2, 128.1, 127.4, 127.3, 127.2, 125.2, 124.8, 121.8, 93.1. HRMS(ESI) *m*/*z* [M + H]^+^ Calcd for C_30_H_18_Cl_2_N_2_O_3_: 525.0694, found: 525.0775.

*7-(2-Chlorobenzoyl)-3-(2-chlorophenyl)-3-hydroxy-2-(quinolin-8-yl)isoindolin-1-one* (**3ad**, new compound): Following the general procedure, the title compound was isolated by flash chromatography (eluent: petrol ether/ethyl acetate = 2/1~1/1) as a white solid in 47% yield (25 mg), mp 114–117 °C. ^1^H NMR (600 MHz, CDCl_3_) δ 8.83 (dd, *J* = 4.4, 1.7 Hz, 1H), 8.18 (dd, *J* = 8.3, 1.7 Hz, 1H), 8.08 (dd, *J* = 6.9, 2.7 Hz, 1H), 7.84 (dd, *J* = 7.6, 1.4 Hz, 1H), 7.69 (d, *J* = 6.7 Hz, 2H), 7.64 (ddd, *J* = 11.0, 7.9, 1.5 Hz, 2H), 7.48–7.44 (m, 2H), 7.41 (dt, *J* = 6.4, 4.6 Hz, 2H), 7.39–7.34 (m, 1H), 7.29–7.27 (m, 1H), 7.15–7.12 (m, 1H), 7.08 (tt, *J* = 7.4, 5.3 Hz, 2H). ^13^C NMR (151 MHz, CDCl_3_) δ 195.1, 167.3, 149.3, 148.2, 143.3, 138.2, 138.1, 137.5, 136.2, 133.4, 133.1, 132.9, 132.5, 132.0, 130.9, 130.9, 130.7, 130.2, 130.0, 129.3, 128.9, 127.2, 127.2, 126.4, 126.2, 124.0, 121.2, 91.5. HRMS(ESI) *m*/*z* [M + H]^+^ Calcd for C_30_H_18_Cl_2_N_2_O_3_: 525.0694, found: 525.0774.

*3-Hydroxy-2-(quinolin-8-yl)-7-(4-(trifluoromethyl)benzoyl)-3-(4-(trifluoromethyl)phenyl)isoindolin-1-one* (**3ae**, new compound): Following the general procedure, the title compound was isolated by flash chromatography (eluent: petrol ether/ethyl acetate = 2/1~1/1) as a white solid in 59% yield (34.9 mg), mp 95–98 °C. ^1^H NMR (600 MHz, CDCl_3_) δ 10.98 (s, 1H), 8.84 (d, *J* = 4.4 Hz, 1H), 8.23 (d, *J* = 8.2 Hz, 1H), 8.01 (d, *J* = 7.9 Hz, 2H), 7.76 (t, *J* = 7.6 Hz, 1H), 7.68 (d, *J* = 8.1 Hz, 3H), 7.57 (dd, *J* = 21.6, 7.9 Hz, 4H), 7.52–7.44 (m, 5H) ^13^C NMR (151 MHz, CDCl_3_) δ 195.3, 167.0, 150.0, 148.6, 143.6, 139.7, 138.3, 136.8, 133.8, 131.8, 129.7 (d, *J* = 256.7 Hz), 128.1, 127.8, 127.1, 126.9, 125.7 (q, *J* = 3.7 Hz), 125.3 (q, *J* = 3.8 Hz), 124.6, 121.5, 92.9. HRMS(ESI) *m*/*z* [M + H]^+^ Calcd for C_32_H_18_F_6_N_2_O_3_: 593.1222, found: 593.1296.

*3-Hydroxy-2-(quinolin-8-yl)-7-(2-(trifluoromethyl)benzoyl)-3-(2-(trifluoromethyl)phenyl)isoindolin-1-one* (**3af**, new compound): Following the general procedure, the title compound was isolated by flash chromatography (eluent: petrol ether/ethyl acetate = 2/1~1/1) as a white solid in 64% yield (37.9 mg), mp 108–110 °C. ^1^H NMR (600 MHz, CDCl_3_) δ 10.15 (s, 1H), 8.69 (dd, *J* = 4.2, 1.6 Hz, 1H), 8.50 (dd, *J* = 7.4, 1.6 Hz, 1H), 8.09 (dd, *J* = 8.3, 1.7 Hz, 1H), 7.68–7.63 (m, 4H), 7.59 (d, *J* = 7.5 Hz, 2H), 7.55–7.48 (m, 5H), 7.46–7.42 (m, 2H), 7.38 (dd, *J* = 8.3, 4.2 Hz, 1H). ^13^C NMR (151 MHz, CDCl_3_) δ 195.2, 165.6, 148.3, 138.6, 137.9, 137.8, 134.6, 134.5, 131.8, 131.2, 130.3, 129.3, 128.9 (q, *J* = 200.8 Hz), 128.1, 127.5, 127.0 (q, *J* = 3.7 Hz), 124.6, 122.8(q, *J* = 3.8 Hz), 122.2, 121.8. HRMS(ESI) *m*/*z* [M + H]^+^ Calcd for C_32_H_18_F_6_N_2_O_3_: 593.1222, found: 593.1296.

*7-(4-Fluorobenzoyl)-3-(4-fluorophenyl)-3-hydroxy-2-(quinolin-8-yl)isoindolin-1-one* (**3ag**, new compound): Following the general procedure, the title compound was isolated by flash chromatography (eluent: petrol ether/ethyl acetate = 2/1~1/1) as a white solid in 51% yield (25 mg), mp 123–126 °C. ^1^H NMR (600 MHz, CDCl_3_) δ 10.10 (s, 1H), 8.81 (dd, *J* = 4.4, 1.8 Hz, 1H), 8.19 (dd, *J* = 8.3, 1.8 Hz, 1H), 7.95–7.90 (m, 2H), 7.72 (t, *J* = 7.6 Hz, 1H), 7.66 (dd, *J* = 8.3, 1.4 Hz, 1H), 7.59–7.35 (m, 7H), 7.10–7.04 (m, 2H), 6.85 (t, *J* = 8.7 Hz, 2H). ^13^C NMR (151 MHz, CDCl_3_) δ 194.6, 166.9, 161.5 (d, *J* = 255.6 Hz), 150.3, 148.5, 143.4, 138.1, 137.2, 135.4 (d, *J* = 3.0 Hz), 133.6 (d, *J* = 2.9 Hz), 133.4, 133.3, 132.2 (d, *J* = 9.4 Hz), 131.8, 129.4, 128.5 (d, *J* = 8.2 Hz), 127.8, 127.5, 126.9 (d, *J* = 14.1 Hz), 124.2, 121.3, 115.6, 115.5 (d, *J* = 21.9 Hz), 114.9, 92.9. HRMS(ESI) *m*/*z* [M + H]^+^ Calcd for C_30_H_18_F_2_N_2_O_3_: 493.1285, found: 493.1358.

*7-(3-Fluorobenzoyl)-3-(3-fluorophenyl)-3-hydroxy-2-(quinolin-8-yl)isoindolin-1-one* (**3ah**, new compound): Following the general procedure, the title compound was isolated by flash chromatography (eluent: petrol ether/ethyl acetate = 2/1~1/1) as a white solid in 40% yield (19.7 mg), mp 108–110 °C. ^1^H NMR (600 MHz, CDCl_3_) δ 10.77 (s, 1H), 8.90–8.78 (m, 1H), 8.22 (s, 1H), 7.74 (t, *J* = 7.6 Hz, 1H), 7.69 (d, *J* = 8.2 Hz, 1H), 7.64 (d, *J* = 8.5 Hz, 2H), 7.56 (t, *J* = 8.4 Hz, 2H), 7.52 (d, *J* = 7.7 Hz, 1H), 7.47 (dt, *J* = 15.9, 7.2 Hz, 2H), 7.38 (q, *J* = 7.3 Hz, 1H), 7.24–7.19 (m, 2H), 7.15 (q, *J* = 7.5 Hz, 1H), 7.09 (d, *J* = 7.9 Hz, 1H), 6.84 (t, *J* = 8.5 Hz, 1H). ^13^C NMR (151 MHz, CDCl_3_) δ 195.0, 166.9, 163.4 (d, *J* = 247.0Hz), 161.8, 150.1, 139.1, 136.9, 133.6, 130.1 (d, *J* = 6.5 Hz), 129.8 (d, *J* = 7.8 Hz), 129.5, 128.0, 124.4, 122.3, 121.4, 120.5 (d, *J* = 21.7 Hz), 115.9 (d, *J* = 22.4 Hz), 115.2 (d, *J* = 23.2 Hz), 114.0, 113.8, 92.8. HRMS(ESI) *m*/*z* [M + H]^+^ Calcd for C_30_H_18_F_2_N_2_O_3_: 493.1285, found: 493.1360.

*7-(2-Fluorobenzoyl)-3-(2-fluorophenyl)-3-hydroxy-2-(quinolin-8-yl)isoindolin-1-one* (**3ai**, new compound): Following the general procedure, the title compound was isolated by flash chromatography (eluent: petrol ether/ethyl acetate = 2/1~1/1) as a white solid in 51% yield (25 mg), mp 125–127 °C. ^1^H NMR (600 MHz, CDCl_3_) δ 8.81 (ddd, *J* = 12.1, 4.4, 1.7 Hz, 1H), 8.18 (ddd, *J* = 28.3, 8.4, 1.8 Hz, 1H), 7.90 (td, *J* = 7.6, 1.9 Hz, 1H), 7.86–7.84 (m, 1H), 7.73–7.64 (m, 2H), 7.63–7.56 (m, 2H), 7.56–7.50 (m, 1H), 7.47–7.39 (m, 3H), 7.23–7.17 (m, 1H), 7.11 (ddt, *J* = 7.3, 5.0, 2.4 Hz, 1H), 7.05 (dd, *J* = 10.9, 8.2 Hz, 1H), 6.95–6.91 (m, 1H), 6.78 (ddd, *J* = 11.6, 8.8, 5.4 Hz, 1H). ^13^C NMR (151 MHz, CDCl_3_) δ 193.2, 167.5, 158.4 ((d, *J* = 249.8 Hz)), 149.3, 148.5, 139.1, 135.0 (d, *J* = 8.9 Hz), 134.9, 133.3, 133.2, 131.5, 130.7 (d, *J* = 8.5 Hz), 129.4 (d, *J* = 2.4 Hz), 127.8, 127.4, 127.3, 124.1 (d, *J* = 3.6 Hz), 123.8, 122.0, 121.4, 116.8, 116.6 (d, *J* = 22.3 Hz), 115.8 (d, *J* = 21.7 Hz), 90.8. HRMS(ESI) *m*/*z* [M + H]^+^ Calcd for C_30_H_18_F_2_N_2_O_3_: 493.1285, found: 493.1364.

*Methyl4-(1-hydroxy-1-(4-(methoxycarbonyl)phenyl)-3-oxo-2-(quinolin-8-yl)isoindoline-4-carbonyl)benzoate* (**3aj**, new compound): Following the general procedure, the title compound was isolated by flash chromatography (eluent: petrol ether/ethyl acetate = 2/1~1/1) as a white solid in 48% yield (27.5 mg), mp 111–113 °C. ^1^H NMR (600 MHz, CDCl_3_) δ 10.80 (s, 1H), 8.81 (dd, *J* = 4.5, 1.8 Hz, 1H), 8.19–8.13 (m, 1H), 8.09–8.05 (m, 2H), 7.94 (d, *J* = 8.4 Hz, 2H), 7.86–7.78 (m, 2H), 7.73 (t, *J* = 7.6 Hz, 1H), 7.65–7.61 (m, 1H), 7.58 (d, *J* = 7.5 Hz, 1H), 7.54 (dd, *J* = 7.6, 1.4 Hz, 1H), 7.52–7.45 (m, 3H), 7.44 (dd, *J* = 8.3, 4.4 Hz, 1H), 7.40 (t, *J* = 7.9 Hz, 1H), 3.87 (s, 3H), 3.81 (s, 3H). ^13^C NMR (151 MHz, CDCl_3_) δ 196.0, 167.3, 166.8, 166.5, 150.4, 149.0, 144.9, 143.7, 140.8, 138.5, 137.3, 134.3, 134.0, 133.5, 132.1, 130.3, 130.0, 129.9, 129.8, 129.7, 128.6, 128.0, 127.4, 127.3, 127.1, 124.9, 121.8, 93.4, 77.6, 77.4, 77.2, 52.7, 52.4. HRMS(ESI) *m*/*z* [M + H]^+^ Calcd for C_34_H_24_N_2_O_7_: 573.1584, found: 573.1572.

*3-Hydroxy-7-(4-methylbenzoyl)-2-(quinolin-8-yl)-3-(p-tolyl)isoindolin-1-one* (**3ak**, new compound): Following the general procedure, the title compound was isolated by flash chromatography (eluent: petrol ether/ethyl acetate = 2/1~1/1) as a white solid in 53% yield (25.6 mg), mp 115–118 °C. ^1^H NMR (600 MHz, CDCl_3_) δ 10.29 (s, *J* = 229.3 Hz, 1H), 8.81 (dt, *J* = 4.4, 2.6 Hz, 1H), 8.17 (d, *J* = 8.2 Hz, 1H), 7.81 (d, *J* = 7.8 Hz, 2H), 7.68 (t, *J* = 7.5 Hz, 1H), 7.61 (dd, *J* = 15.7, 7.9 Hz, 2H), 7.50 (d, *J* = 7.4 Hz, 1H), 7.47 (d, *J* = 7.6 Hz, 1H), 7.43 (q, *J* = 6.9 Hz, 2H), 7.28 (d, *J* = 7.9 Hz, 2H), 7.21 (d, *J* = 8.0 Hz, 2H), 6.96 (d, *J* = 7.9 Hz, 2H), 2.34 (s, 3H), 2.19 (s, 3H). ^13^C NMR (151 MHz, CDCl_3_) δ 196.0, 167.2, 150.7, 144.2, 137.7, 136.6, 134.7, 133.2, 129.8, 129.4, 129.1, 128.9, 128.7, 127.6, 127.2, 126.5, 123.9, 121.2, 93.3, 21.6, 20.9. HRMS(ESI) *m*/*z* [M + H]^+^ Calcd for C_32_H_24_N_2_O_3_: 485.1787, found: 485.1864.

*3-Hydroxy-7-(3-methylbenzoyl)-2-(quinolin-8-yl)-3-(m-tolyl)isoindolin-1-one* (**3al**, new compound): Following the general procedure, the title compound was isolated by flash chromatography (eluent: petrol ether/ethyl acetate = 2/1~1/1) as a white solid in 67% yield (32.4 mg), mp 122–125 °C. ^1^H NMR (600 MHz, CDCl_3_) δ 10.66 (s, 1H), 8.86–8.76 (m, 1H), 8.15 (d, *J* = 8.3 Hz, 1H), 7.80 (s, 1H), 7.70 (dd, *J* = 17.2, 8.4 Hz, 2H), 7.63 (d, *J* = 7.9 Hz, 2H), 7.53 (dd, *J* = 11.6, 7.6 Hz, 2H), 7.43 (q, *J* = 6.7 Hz, 2H), 7.37–7.25 (m, 4H), 7.08 (t, *J* = 7.6 Hz, 1H), 6.96 (d, *J* = 7.5 Hz, 1H), 2.37 (s, 3H), 2.22 (s, 3H). ^13^C NMR (151 MHz, CDCl_3_) δ 196.6, 167.2, 150.7, 148.4, 143.6, 139.4, 138.1, 137.9, 137.7, 137.6, 137.1, 134.3, 133.6, 133.2, 132.0, 129.8, 129.4, 128.8, 128.2, 127.9, 127.5, 127.3, 127.2, 127.1, 126.9, 124.0, 123.7, 121.2, 93.3, 21.3, 21.2. HRMS(ESI) *m*/*z* [M + H]^+^ Calcd for C_32_H_24_N_2_O_3_: 485.1787, found: 485.1864.

*7-Benzoyl-3-hydroxy-5-methyl-3-phenyl-2-(quinolin-8-yl)isoindolin-1-one* (**3ba**, new compound): Following the general procedure, the title compound was isolated by flash chromatography (eluent: petrol ether/ethyl acetate = 2/1~1/1) as a white solid in 56% yield (26.4 mg), mp 125–127 °C. ^1^H NMR (600 MHz, CDCl_3_) δ 10.71 (s, 1H), 8.78 (d, *J* = 4.5 Hz, 1H), 8.12 (d, *J* = 8.3 Hz, 1H), 7.92 (d, *J* = 7.7 Hz, 2H), 7.59 (dd, *J* = 13.5, 7.8 Hz, 2H), 7.50 (t, *J* = 7.7 Hz, 1H), 7.45–7.37 (m, 6H), 7.31 (d, *J* = 27.5 Hz, 2H), 7.19–7.11 (m, 3H), 2.45 (s, 3H); ^13^C NMR (151 MHz, CDCl_3_) δ 196.7, 167.4, 151.1, 148.6, 144.7, 143.7, 139.9, 138.2, 137.6, 137.3, 133.9, 133.5, 132.0, 129.8, 129.5, 128.7, 128.5, 128.2, 128.1, 127.3, 127.0, 126.8, 124.7, 124.7, 121.4, 93.3, 22.0. HRMS(ESI) *m*/*z* [M + H]^+^ Calcd for C_31_H_22_N_2_O_3_: 471.1630, found: 471.1706.

*7-Benzoyl-3-hydroxy-5-methoxy-3-phenyl-2-(quinolin-8-yl)isoindolin-1-one* (**3ca**, new compound): Following the general procedure, the title compound was isolated by flash chromatography (eluent: petrol ether/ethyl acetate = 2/1~1/1) as a white solid in 49% yield (23.8 mg), mp 118–120 °C.^1^H NMR (600 MHz, CDCl_3_) δ 8.81 (dd, *J* = 4.4, 1.7 Hz, 1H), 8.15 (dd, *J* = 8.3, 1.7 Hz, 1H), 8.10–8.06 (m, 1H), 7.95–7.91 (m, 2H), 7.60 (dd, *J* = 8.0, 1.4 Hz, 1H), 7.56 (d, *J* = 7.5 Hz, 1H), 7.53–7.49 (m, 1H), 7.46 (t, *J* = 7.7 Hz, 1H), 7.43–7.39 (m, 5H), 7.19–7.15 (m, 2H), 7.15–7.12 (m, 1H), 7.03 (d, *J* = 2.2 Hz, 1H), 6.94 (d, *J* = 2.2 Hz, 1H), 3.84 (s, 3H); ^13^C NMR (151 MHz, CDCl_3_) δ 196.1, 167.1, 164.1, 153.2, 148.5, 139.9, 139.2, 137.0, 133.7, 133.6, 132.1, 130.2, 129.9, 129.5, 128.6, 128.5, 128.2, 128.2, 127.3, 127.1, 126.7, 121.4, 119.7, 114.9, 108.6, 93.1, 56.1. HRMS(ESI) *m*/*z* [M + H]^+^ Calcd for C_31_H_22_N_2_O_4_: 487.1580, found: 487.1657.

*7-Benzoyl-5-(tert-butyl)-3-hydroxy-3-phenyl-2-(quinolin-8-yl)isoindolin-1-one* (**3da,** new compound): Following the general procedure, the title compound was isolated by flash chromatography (eluent: petrol ether/ethyl acetate = 2/1~1/1) as a white solid in 58% yield (29.7 mg), mp 117–120 °C. ^1^H NMR (600 MHz, CDCl_3_) δ 10.77 (s, 1H), 8.80 (dd, *J* = 4.5, 1.8 Hz, 1H), 8.15 (dd, *J* = 8.2, 1.9 Hz, 1H), 7.95–7.91 (m, 2H), 7.62–7.58 (m, 1H), 7.57 (d, *J* = 1.6 Hz, 1H), 7.55 (dd, *J* = 7.6, 1.3 Hz, 1H), 7.53–7.49 (m, 2H), 7.44–7.37 (m, 6H), 7.15 (dt, *J* = 15.0, 6.9 Hz, 3H), 1.34 (s, 9H). ^13^C NMR (151 MHz, CDCl_3_) δ 197.08, 167.31, 158.04, 150.82, 148.58, 143.80, 139.96, 138.17, 137.36, 137.21, 133.86, 133.53, 132.09, 129.87, 129.51, 128.55, 128.16, 128.11, 127.37, 127.07, 126.75, 125.51, 124.80, 121.41, 121.04, 93.57, 35.86, 31.34. HRMS(ESI) *m*/*z* [M + H]^+^ Calcd for C_34_H_28_N_2_O_3_: 513.2100, found: 513.2176.

*7-Benzoyl-5-fluoro-3-hydroxy-3-phenyl-2-(quinolin-8-yl)isoindolin-1-one* (**3ea**, new compound): Following the general procedure, the title compound was isolated by flash chromatography (eluent: petrol ether/ethyl acetate = 2/1~1/1) as a white solid in 63% yield (29.9 mg), mp 97–100 °C. ^1^H NMR (600 MHz, CDCl_3_) δ 11.27–10.51 (s, 1H), 8.81 (dd, *J* = 4.4, 1.7 Hz, 1H), 8.17 (dd, *J* = 8.4, 1.8 Hz, 1H), 7.91 (d, *J* = 7.6 Hz, 2H), 7.62 (dd, *J* = 15.5, 7.9 Hz, 2H), 7.54 (t, *J* = 7.4 Hz, 1H), 7.46–7.40 (m, 6H), 7.25–7.22 (m, 1H), 7.21–7.13 (m, 4H). ^13^C NMR (151 MHz, CDCl_3_) δ 194.8, 166.4, 165.68 (d, *J* = 214.0 Hz), 148.7, 143.6, 139.2, 138.3, 136.7, 133.9, 133.5, 132.1, 129.8, 129.6, 128.7, 128.5, 128.4, 127.6, 127.1, 126.7, 121.5, 115.9 (d, *J* = 24.9 Hz), 111.8 (d, *J* = 23.8 Hz), 93.0. HRMS(ESI) *m*/*z* [M + H]^+^ Calcd for C_30_H_19_FN_2_O_3_: 475.1380, found: 475.1459.

*7-Benzoyl-5-chloro-3-hydroxy-3-phenyl-2-(quinolin-8-yl)isoindolin-1-one* (**3fa**, new compound): Following the general procedure, the title compound was isolated by flash chromatography (eluent: petrol ether/ethyl acetate = 2/1~1/1) as a white solid in 47% yield (23 mg), mp 96–100 °C. ^1^H NMR (600 MHz, CDCl_3_) δ 10.77 (s, 1H), 8.81 (dd, *J* = 4.4, 1.6 Hz, 1H), 8.17 (d, *J* = 8.3 Hz, 1H), 7.91 (d, *J* = 7.6 Hz, 2H), 7.64 (d, *J* = 8.2 Hz, 1H), 7.60 (d, *J* = 7.5 Hz, 1H), 7.57–7.49 (m, 2H), 7.47–7.39 (m, 7H), 7.19 (t, *J* = 7.1 Hz, 2H), 7.16 (dd, *J* = 8.3, 6.0 Hz, 1H). ^13^C NMR (151 MHz, CDCl_3_) δ 194.8, 166.4, 152.4, 148.7, 143.6, 139.9, 139.2, 139.1, 138.3, 136.8, 133.9, 133.4, 132.1, 129.9, 129.6, 128.7, 128.5, 128.4, 128.2, 127.7, 127.1, 126.7, 125.5, 124.7, 121.5, 93.1. HRMS(ESI) *m*/*z* [M + H]^+^ Calcd for C_30_H_19_ClN_2_O_3_: 491.1084, found: 491.1161.

*7-Benzoyl-3-hydroxy-1-oxo-3-phenyl-2-(quinolin-8-yl)isoindoline-5-carboxylate* (**3ga**, new compound): Following the general procedure, the title compound was isolated by flash chromatography (eluent: petrol ether/ethyl acetate = 2/1~1/1) as a white solid in 58% yield (29.8 mg), mp 113–115 °C. ^1^H NMR (600 MHz, CDCl_3_) δ 8.83 (dd, *J* = 4.3, 2.0 Hz, 1H), 8.20 (d, *J* = 8.2 Hz, 2H), 8.13 (d, *J* = 1.6 Hz, 1H), 7.89 (d, *J* = 7.6 Hz, 2H), 7.67 (dd, *J* = 8.2, 1.8 Hz, 1H), 7.61 (dd, *J* = 7.6, 1.5 Hz, 1H), 7.54 (d, *J* = 7.3 Hz, 1H), 7.49–7.46 (m, 1H), 7.43 (ddd, *J* = 14.5, 8.4, 5.7 Hz, 5H), 7.17 (dt, *J* = 14.9, 6.9 Hz, 3H), 3.92 (d, *J* = 1.9 Hz, 3H). ^13^C NMR (151 MHz, CDCl_3_) δ 195.3, 166.3, 165.4, 150.9, 148.6, 143.4, 138.9, 138.1, 137.7, 136.7, 134.7, 133.7, 133.3, 131.9, 130.5, 129.7, 129.4, 129.1, 128.5, 128.3, 128.2, 127.6, 127.0, 126.6, 125.3, 121.4, 93.3, 52.6. HRMS(ESI) *m*/*z* [M + H]^+^ Calcd for C_32_H_22_N_2_O_5_: 515.1529, found: 515.1609.

*7-Benzoyl-3-hydroxy-3-phenyl-2-(quinolin-8-yl)-5-vinylisoindolin-1-one* (**3ha**, new compound): Following the general procedure, the title compound was isolated by flash chromatography (eluent: petrol ether/ethyl acetate = 2/1~1/1) as a white solid in 55% yield (26.5 mg), mp 120–123 °C. ^1^H NMR (600 MHz, CDCl_3_) δ 10.61 (s, *J* = 99.1 Hz, 1H), 8.81 (d, *J* = 4.6 Hz, 1H), 8.15 (d, *J* = 8.4 Hz, 1H), 7.94 (d, *J* = 7.7 Hz, 2H), 7.60 (dd, *J* = 12.8, 7.8 Hz, 2H), 7.55 (s, 1H), 7.53–7.48 (m, 2H), 7.45–7.37 (m, 6H), 7.21–7.11 (m, 3H), 6.76 (dd, *J* = 17.6, 10.8 Hz, 1H), 5.87 (d, *J* = 17.6 Hz, 1H), 5.41 (d, *J* = 10.9 Hz, 1H). ^13^C NMR (151 MHz, CDCl_3_) δ 196.2, 166.8, 151.2, 148.4, 142.9, 139.5, 137.8, 137.0, 135.3, 133.4, 129.7, 129.3, 128.4, 128.1, 128.0, 126.9, 126.6, 126.2, 125.8, 121.5, 121.2, 117.7, 93.2. HRMS(ESI) *m*/*z* [M + H]^+^ Calcd for C_32_H_22_N_2_O_3_: 483.5390, found: 483.1703.

*7-Benzoyl-3-hydroxy-3,5-diphenyl-2-(quinolin-8-yl)isoindolin-1-one* (**3ia**, new compound): Following the general procedure, the title compound was isolated by flash chromatography (eluent: petrol ether/ethyl acetate = 2/1~1/1) as a white solid in 62% yield (32.9 mg), mp 118–121 °C. ^1^H NMR (600 MHz, DMSO-d_6_) δ 8.83 (dd, *J* = 4.2, 1.8 Hz, 1H), 8.32 (d, *J* = 8.3 Hz, 1H), 7.89 (ddd, *J* = 13.3, 5.6, 3.2 Hz, 4H), 7.75–7.72 (m, 2H), 7.69–7.65 (m, 2H), 7.57 (ddd, *J* = 29.5, 14.9, 7.5 Hz, 5H), 7.50–7.45 (m, 3H), 7.43–7.40 (m, 1H), 7.17–7.09 (m, 3H). ^13^C NMR (151 MHz, DMSO-d_6_) δ 195.4, 165.0, 151.3, 149.9, 145.0, 143.7, 139.3, 138.2, 137.3, 136.7, 133.7, 133.4, 129.5, 129.3, 128.8, 128.7, 128.6, 128.2, 128.1, 127.8, 127.3, 127.1, 126.7, 126.6, 126.1, 126.0, 122.0, 121.6, 92.7. HRMS(ESI) *m*/*z* [M + H]^+^ Calcd for C_36_H_24_N_2_O_3_: 533.1787, found: 533.1861.

### 3.4. General Procedure for the Synthesis of Compound ***4***

A Schlenk tube was equipped with a magnetic stir bar and charged with 2-methyl-*N*-(quinolin-8-yl)benzamide **1i** (0.1 mmol), **2** (0.25 mmol), NaOAc (0.2 mmol, 16 mg), Pd(OAc)_2_ (0.01 mmol, 2.3 mg), and xylene (2 mL). Then, the flask was sealed under N_2_ and stirred at 80 °C for 16 h. After the reaction was quenched by the addition of water, the mixture was extracted with dichloromethane, and the combined organic layer was dried over sodium sulfate. The concentration in vacuo followed by silica gel column purification with petroleum ether/ethyl acetate eluent gave the desired product 4.

*3-Hydroxy-7-methyl-3-phenyl-2-(quinolin-8-yl)isoindolin-1-one* (**4a**, new compound): Following the general procedure, the title compound was isolated by flash chromatography (eluent: petrol ether/ethyl acetate = 2/1~1/1) as a white solid in 62% yield (22.7 mg), mp 130–132 °C. ^1^H NMR (600 MHz, CDCl_3_) δ 9.85 (s, 1H), 8.80 (dd, *J* = 4.3, 1.7 Hz, 1H), 8.18 (dd, *J* = 8.3, 1.8 Hz, 1H), 7.69 (dd, *J* = 8.2, 1.4 Hz, 1H), 7.59 (dd, *J* = 7.5, 1.4 Hz, 1H), 7.50–7.44 (m, 2H), 7.43–7.40 (m, 3H), 7.29 (d, *J* = 7.6 Hz, 1H), 7.20–7.10 (m, 4H), 2.81 (s, 3H). ^13^C NMR (151 MHz, CDCl_3_) δ 170.2, 151.4, 149.3, 140.6, 138.3, 138.2, 133.2, 132.6, 131.4, 129.8, 128.3, 128.1, 127.9, 127.2, 126.9, 126.8, 121.7, 120.6, 92.7, 17.9. HRMS(ESI) *m*/*z* [M + Na]^+^ Calcd for C_24_H_18_N_2_O_2_: 389.1368, found: 389.1262.

*3-(4-Chlorophenyl)-3-hydroxy-7-methyl-2-(quinolin-8-yl)isoindolin-1-one* (**4b**, new compound): Following the general procedure, the title compound was isolated by flash chromatography (eluent: petrol ether/ethyl acetate = 2/1~1/1) as a white solid in 62% yield (24.8 mg), mp 107–109 °C. ^1^H NMR (600 MHz, CDCl_3_) δ 10.08–9.88 (m, 1H), 8.79 (d, *J* = 4.3 Hz, 1H), 8.20 (d, *J* = 8.3 Hz, 1H), 7.72 (d, *J* = 8.2 Hz, 1H), 7.59 (d, *J* = 7.4 Hz, 1H), 7.52–7.41 (m, 3H), 7.34 (d, *J* = 8.5 Hz, 2H), 7.29 (d, *J* = 7.6 Hz, 1H), 7.14 (dd, *J* = 15.7, 7.9 Hz, 3H), 2.80 (s, 3H). ^13^C NMR (151 MHz, CDCl_3_) δ 170.0, 151.0, 149.3, 139.4, 138.4, 138.4, 134.1, 133.3, 132.5, 131.7, 129.9, 128.5, 128.5, 128.1, 127.4, 126.7, 121.8, 120.5, 92.3, 17.9. HRMS(ESI) *m*/*z* [M + Na]^+^ Calcd for C_24_H_17_ClN_2_O_2_: 423.0979, found: 423.0876.

*3-(4-Fluorophenyl)-3-hydroxy-7-methyl-2-(quinolin-8-yl)isoindolin-1-one* (**4c**, new compound): Following the general procedure, the title compound was isolated by flash chromatography (eluent: petrol ether/ethyl acetate = 2/1~1/1) as a white solid in 54% yield (20.7 mg), mp 114–116 °C. ^1^H NMR (600 MHz, CDCl_3_) δ 9.96 (d, *J* = 90.1 Hz, 1H), 8.84–8.79 (m, 1H), 8.23 (d, *J* = 8.0 Hz, 1H), 7.74 (d, *J* = 8.1 Hz, 1H), 7.56 (dd, *J* = 7.5, 1.5 Hz, 1H), 7.52 (d, *J* = 7.9 Hz, 1H), 7.47 (t, *J* = 7.6 Hz, 2H), 7.40–7.33 (m, 2H), 7.30 (d, *J* = 7.6 Hz, 1H), 7.17 (d, *J* = 7.5 Hz, 1H), 6.84 (t, *J* = 8.7 Hz, 2H), 2.80 (s, 3H). ^13^C NMR (151 MHz, CDCl_3_) δ 169.6, 163.0 (d, *J* = 246.6 Hz), 150.7, 148.9, 138.0, 136.1 (d, *J* = 3.0 Hz), 132.9, 132.1, 131.2, 129.5, 128.5 (d, *J* = 8.3 Hz), 127.6, 126.9, 126.3, 121.4, 120.1, 114.9, 114.7, 92.0, 17.5. HRMS(ESI) *m*/*z* [M + H]^+^ Calcd for C_24_H_17_FN_2_O_2_: 385.1274, found: 385.1346.

*3-Hydroxy-7-methyl-3-(4-nitrophenyl)-2-(quinolin-8-yl)isoindolin-1-one* (**4d**, new compound): Following the general procedure, the title compound was isolated by flash chromatography (eluent: petrol ether/ethyl acetate = 2/1~1/1) as a white solid in 48% yield (19.7 mg), mp 118–120 °C. ^1^H NMR (600 MHz, CDCl_3_) δ 10.28 (s, 1H), 8.82 (tt, *J* = 4.5, 1.3 Hz, 1H), 8.25 (ddt, *J* = 8.5, 4.1, 1.8 Hz, 1H), 8.04–7.99 (m, 2H), 7.75 (ddt, *J* = 8.1, 3.4, 1.6 Hz, 1H), 7.60 (t, *J* = 8.5 Hz, 3H), 7.55–7.45 (m, 3H), 7.35–7.30 (m, 1H), 7.13 (d, *J* = 7.5 Hz, 1H), 2.80 (s, 3H). ^13^C NMR (151 MHz, CDCl_3_) δ 169.8, 150.3, 149.4, 148.3, 147.8, 144.3, 138.7, 138.5, 133.7, 133.5, 132.4, 132.0, 129.9, 128.3, 128.1, 127.4, 126.6, 123.7, 121.9, 120.5, 91.9, 17.9. HRMS(ESI) *m*/*z* [M + H]^+^ Calcd for C_24_H_17_N_3_O_4_: 412.1219, found: 412.1286.

*3-Hydroxy-7-methyl-2-(quinolin-8-yl)-3-(4-(trifluoromethyl)phenyl)isoindolin-1-one* (**4e**, new compound): Following the general procedure, the title compound was isolated by flash chromatography (eluent: petrol ether/ethyl acetate = 2/1~1/1) as a white solid in 53% yield (23.0 mg), mp 123–126 °C. ^1^H NMR (600 MHz, CDCl_3_) δ 10.11 (s, 1H), 8.82 (d, *J* = 4.3 Hz, 1H), 8.23 (d, *J* = 8.3 Hz, 1H), 7.74 (d, *J* = 8.2 Hz, 1H), 7.60 (d, *J* = 7.4 Hz, 1H), 7.54 (t, *J* = 9.0 Hz, 3H), 7.46 (dt, *J* = 21.6, 7.9 Hz, 4H), 7.31 (d, *J* = 7.6 Hz, 1H), 7.14 (d, *J* = 7.5 Hz, 1H), 2.81 (s, 3H). ^13^C NMR (151 MHz, CDCl_3_) δ 170.0, 150.8, 149.4, 145.0, 138.6, 138.4, 133.9 (d, *J* = 3.4 Hz), 132.6, 131.8, 129.9 (d, *J* = 205.4 Hz), 128.2, 127.5 (d, *J* = 6.5 Hz), 126.6, 125.4, 125.4, 125.4, 125.4, 121.9, 120.6, 92.2, 17.9. HRMS(ESI) *m*/*z* [M + H]^+^ Calcd for C_25_H_17_F_3_N_2_O_2_: 457.1242, found: 457.1136.

*3-Hydroxy-7-methyl-2-(quinolin-8-yl)-3-(p-tolyl)isoindolin-1-one* (**4f**, new compound): Following the general procedure, the title compound was isolated by flash chromatography (eluent: petrol ether/ethyl acetate = 2/1~1/1) as a white solid in 69% yield (26.2 mg), mp 116–119 °C. ^1^H NMR (600 MHz, CDCl_3_) δ 9.81 (s, 1H), 8.80 (dd, *J* = 4.4, 1.7 Hz, 1H), 8.20–8.16 (m, 1H), 7.70 (dd, *J* = 8.2, 1.4 Hz, 1H), 7.62–7.58 (m, 1H), 7.51–7.41 (m, 3H), 7.28 (t, *J* = 7.9 Hz, 3H), 7.18 (d, *J* = 7.5 Hz, 1H), 6.97 (d, *J* = 8.0 Hz, 2H), 2.81 (s, 3H), 2.21 (s, 3H). ^13^C NMR (151 MHz, CDCl_3_) δ 169.75, 151.14, 148.84, 137.78, 137.40, 137.19, 132.75, 130.94, 129.39, 128.60, 127.42, 126.84, 126.43, 126.30, 121.24, 120.16, 92.37, 20.91, 17.48. HRMS(ESI) *m*/*z* [M + Na]^+^ Calcd for C_25_H_20_N_2_O_2_: 403.1525, found: 403.1424.

*3-Hydroxy-7-methyl-2-(quinolin-8-yl)-3-(m-tolyl)isoindolin-1-one* (**4g**, new compound): Following the general procedure, the title compound was isolated by flash chromatography (eluent: petrol ether/ethyl acetate = 2/1~1/1) as a white solid in 46% yield (17.5 mg), mp 115–117 °C. ^1^H NMR (600 MHz, CDCl_3_) δ 9.69 (s, 1H), 8.81 (d, *J* = 4.2 Hz, 1H), 8.20 (d, *J* = 8.3 Hz, 1H), 7.72 (d, *J* = 8.2 Hz, 1H), 7.59 (d, *J* = 7.4 Hz, 1H), 7.52–7.43 (m, 3H), 7.28 (d, *J* = 7.6 Hz, 1H), 7.24 (s, 1H), 7.19 (t, *J* = 6.7 Hz, 2H), 7.06 (t, *J* = 7.6 Hz, 1H), 6.95 (d, *J* = 7.5 Hz, 1H), 2.80 (s, 3H), 2.20 (s, 3H). ^13^C NMR (151 MHz, CDCl_3_) δ 169.8, 151.2, 140.1, 137.9, 137.5, 132.8, 131.0, 129.5, 128.5, 127.8, 127.5, 127.1, 123.7, 121.3, 120.2, 92.4, 21.3, 17.5. HRMS(ESI) *m*/*z* [M + Na]^+^ Calcd for C_25_H_20_N_2_O_2_: 403.1525, found: 403.1424.

*3-(Furan-2-yl)-3-hydroxy-7-methyl-2-(quinolin-8-yl)isoindolin-1-one* (**4h**, new compound): Following the general procedure, the title compound was isolated by flash chromatography (eluent: petrol ether/ethyl acetate = 2/1~1/1) as a white solid in 57% yield (20.3 mg), mp 122–125 °C. ^1^H NMR (600 MHz, CDCl_3_) δ 8.87 (dd, *J* = 4.4, 1.8 Hz, 1H), 8.31 (dd, *J* = 8.3, 1.7 Hz, 1H), 7.84 (dd, *J* = 8.2, 1.3 Hz, 2H), 7.64 (s, 1H), 7.59 (d, *J* = 7.5 Hz, 1H), 7.53 (dd, *J* = 8.3, 4.4 Hz, 1H), 7.42 (dd, *J* = 15.2, 7.5 Hz, 2H), 7.26 (d, *J* = 1.4 Hz, 1H), 6.50 (d, *J* = 3.3 Hz, 1H), 6.20 (dd, *J* = 3.3, 1.8 Hz, 1H), 2.88 (s, 3H). ^13^C NMR (151 MHz, CDCl_3_) δ 169.2, 148.8, 147.9, 142.7, 138.0, 132.6, 131.5, 129.3, 127.5, 127.5, 126.9, 126.5, 121.3, 119.9, 109.9, 109.3, 88.9, 17.4. HRMS(ESI) *m*/*z* [M + Na]^+^ Calcd for C_22_H_16_N_2_O_3_: 379.1161, found: 379.1054.

## 4. Conclusions

In conclusion, we have developed a novel dual C-H acylations and intramolecular cyclization sequence using bidentate *N*-(quinolin-8-yl)benzamide and aromatic acyl chlorides under oxidant-free and ligand-free conditions. This process involves the formation of three new chemical bonds and provides straightforward access to new hydroxyl isoindolono-quinoline skeletons. Further studies and applications of this innovative synthetic methodology are currently underway in our laboratory.

## Figures and Tables

**Table 1:** Optimization of the reaction conditions ^a^.

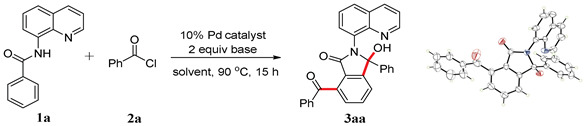				
Entry	Catalyst	Base	Solvent	Yield (%) ^b^
1	Pd(OAc)_2_	KOAc	1 mL Toluene	39
2	Pd(OAc)_2_	Na_2_CO_3_	1 mL Toluene	62
3	Pd(OAc)_2_	LiOAc	1 mL Toluene	68
4	Pd(OAc)_2_	NaOAc	1 mL Toluene	72
5	Pd(OAc)_2_	HCOONa	1 mL Toluene	trace
6	Pd(OAc)_2_	DBU	1 mL Toluene	N.R.
7	Pd(OAc)_2_	K_2_CO_3_	1 mL Toluene	53
8	Pd(OAc)_2_	K_3_PO_4_	1 mL Toluene	41
9	PdCl_2_(MeCN)_2_	NaOAc	1 mL Toluene	60
10	PdCl_2_	NaOAc	1 mL Toluene	55
11	Pd_2_(dba)_3_	NaOAc	1 mL Toluene	68
12	Pd(TFA)2	NaOAc	1 mL Toluene	64
13	Pd(OAc)_2_	NaOAc	1 mL DMF	13
14	Pd(OAc)_2_	NaOAc	1 mL MeCN	18
15	Pd(OAc)_2_	NaOAc	1 mL DCE	44
16	Pd(OAc)_2_	NaOAc	1 mL 1,4-dioxane	22
17	Pd(OAc)_2_	NaOAc	1 mL DMSO	15
18	Pd(OAc)_2_	NaOAc	1 mL Xylene	76
19	Pd(OAc)_2_	NaOAc	1 mL PhCl	63
20	Pd(OAc)_2_	NaOAc	1 mL Benzene	54
21	Pd(OAc)_2_	NaOAc	0.5 mL Xylene	68
22	Pd(OAc)_2_	NaOAc	2 mL Xylene	83
23	Pd(OAc)_2_	NaOAc	2 mL Xylene	54 ^c^
24	Pd(OAc)_2_	NaOAc	2 mL Xylene	55 ^d^
25	Pd(OAc)_2_	NaOAc	2 mL Xylene	73 ^e^
26	Pd(OAc)_2_	NaOAc	2 mL Xylene	67 ^f^
27	Pd(OAc)_2_	NaOAc	2 mL Xylene	60 ^g^
28	Pd(OAc)_2_	NaOAc	2 mL Xylene	68 ^h^
29	Pd(OAc)_2_	NaOAc	2 mL Xylene	42 ^i^
30	-	NaOAc	2 mL Xylene	N.R.
31	Pd(OAc)_2_	-	2 mL Xylene	N.R.

^a^ Reaction condition 1a (0.1 mmol), 2a (0.25 mmol), Pd(OAc)_2_ (0.01 mmol), and NaOAc (0.2 mmol) in 2 mL dry xylene at 90 °C under N_2_ for 16 h. ^b^ Isolated yield. ^c^ Reaction temperature was 80 °C. ^d^ Reaction temperature was 120 °C. ^e^ Reaction time was 24 h. ^f^ With 5 mol% Pd(OAc)_2_ catalyst. ^g^ With 2.5 mol% Pd(OAc)_2_ catalyst. ^h^ Under air. ^i^ Benzyol bromide as the acylation reagent.

## Data Availability

Data are contained within the article and Supplementary Materials.

## Supplementary Materials

The following supporting information can be downloaded at: https://www.mdpi.com/article/10.3390/molecules29225397/s1. Kinetic isotope effect measurements. Copies of ^1^H and ^13^C NMR Spectra for products. Single-crystal information (**3aa** and **4a**).
