# Supplementary material for: Pd-Catalyzed Aromatic Dual C-H Acylations and Intramolecular Cyclization: Access to Quinoline-Substituted Hydroxyl Isoindolones
Source: Molecules. 2024 Nov 15;29(22):5397. doi: 10.3390/molecules29225397 (PMC11596677; doi:10.3390/molecules29225397)
Supplement: Supplementary file 1 [file molecules-29-05397-s001.zip › molecules-3303667-supplementary.pdf]

Supporting Information

Pd-Catalyzed Aromatic Dual C-H Acylations and Intramolecular Cyclization: Access to Quinoline-Substituted Hydroxyl Isoindolones

Hongke Xu[a,b], Yuchen Yang[a,b], Fei Li[a,b] and Yuzhu Yang[a,b]*

[a]State Key Laboratory of Functions and Applications of Medicinal Plants, Guizhou Medical University, 3491 Gaohai Road, Guiyang 550014, P. R. China

[b]Natural Products Research Center of Guizhou Province, 3491 Baijin Road, Guiyang 550014, P. R. China.

Email: yangyuzhu@gmc.edu.cn

Contents:

1. Kinetic isotope effect measurements

2. Copies of ^{1}H and ^{13}C NMR Spectra for products

3. Single-crystal information (**3aa** and **4a**)

1. Kinetic isotope effect measurements

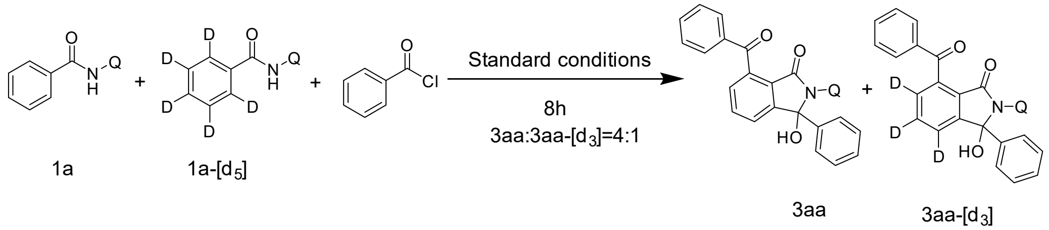

A Schlenk tube was equipped with a magnetic stir bar and charged with N-(quinolin-8-yl)benzamide 1a (0.1 mmol, 24.8mg), 1a-d_5 (0.1 mmol, 24.8mg), 2a (0.5 mmol, 58μL), NaOAc (0.4 mmol, 32mg), $Pd(OAc)_2$ (0.02 mmol, 4.6mg), and Xylene (4mL). Then, the flask was sealed under N2 and stirred at 90° C for 8 h. After the reaction was quenched by the addition of water, the mixture was extracted with dichloromethane, and the combined organic layer was dried over sodium sulfate. The concentration in vacuo followed by silica gel column purification with petroleum ether/ethyl acetate eluent gave the desired product 1a/1a-d1, and then this was analyzed by the ^{1}H NMR spectrum.

The KIE value was calculated as $k_H/k_D = 4.0$.

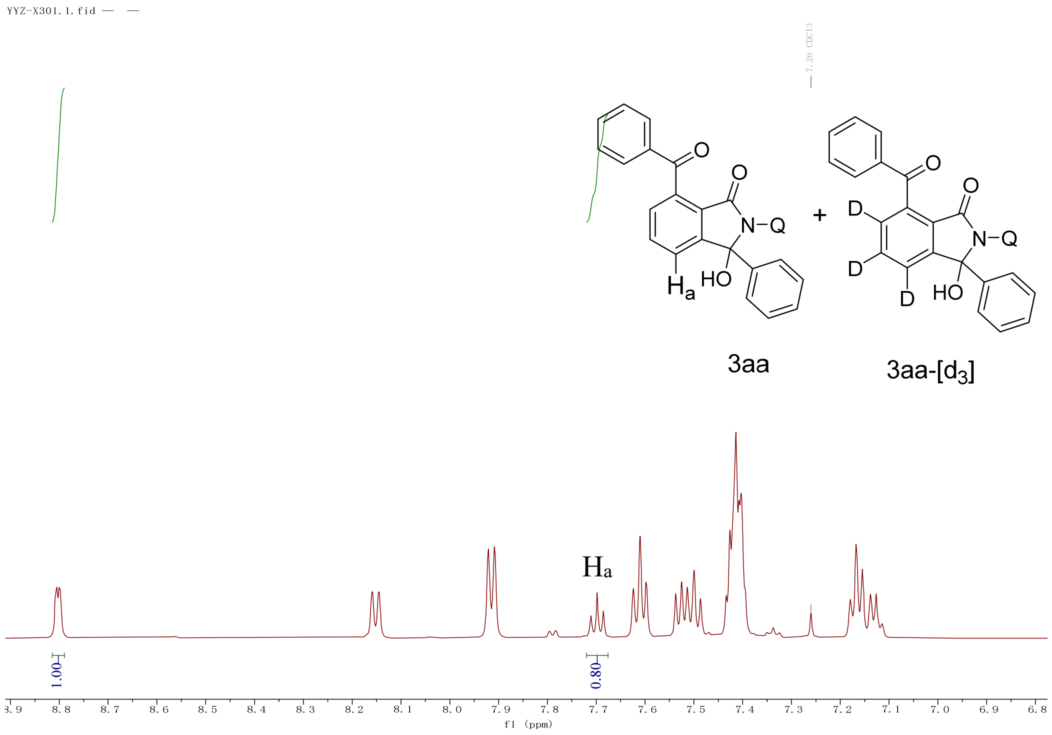

In the ^{1}H NMR spectra with chemical shift 7.7 ppm, Ha, the KIE value can be calculated via $k_H/k_D=0.8/(1-0.8) = 4$

2. Copies of ^{1}H and ^{13}C NMR Spectra for products

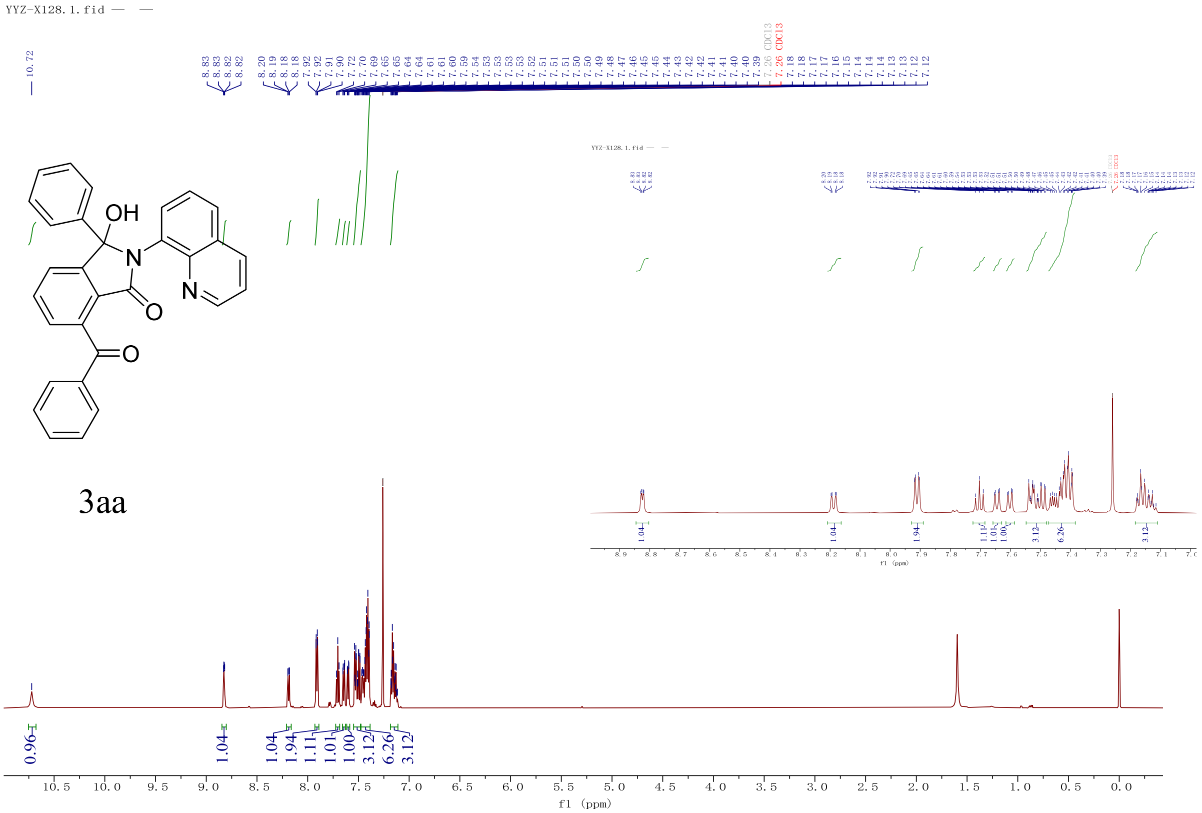

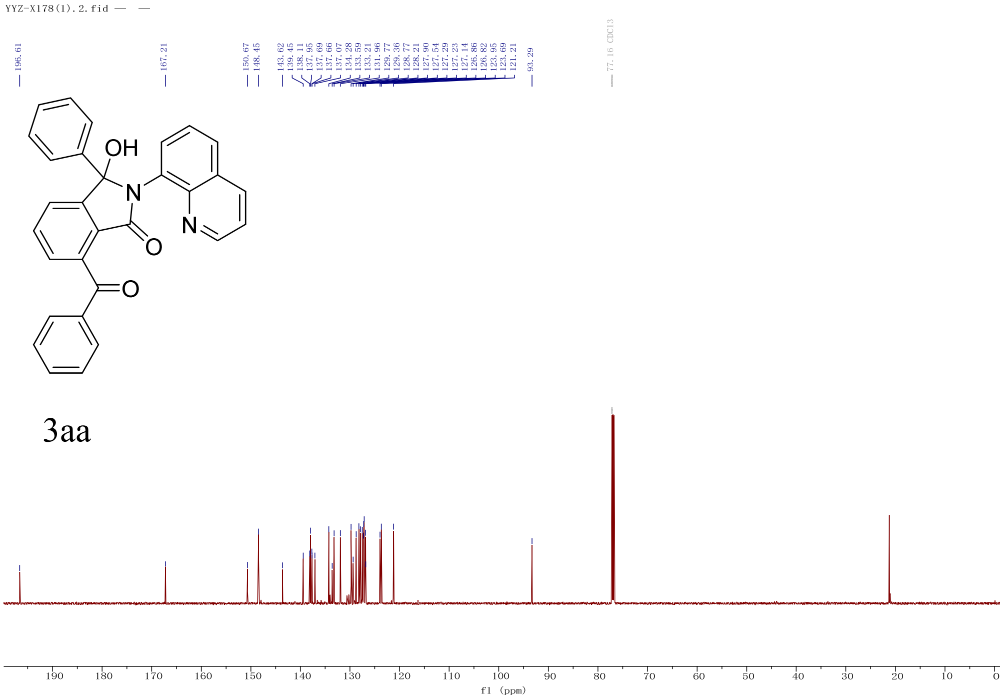

3ab

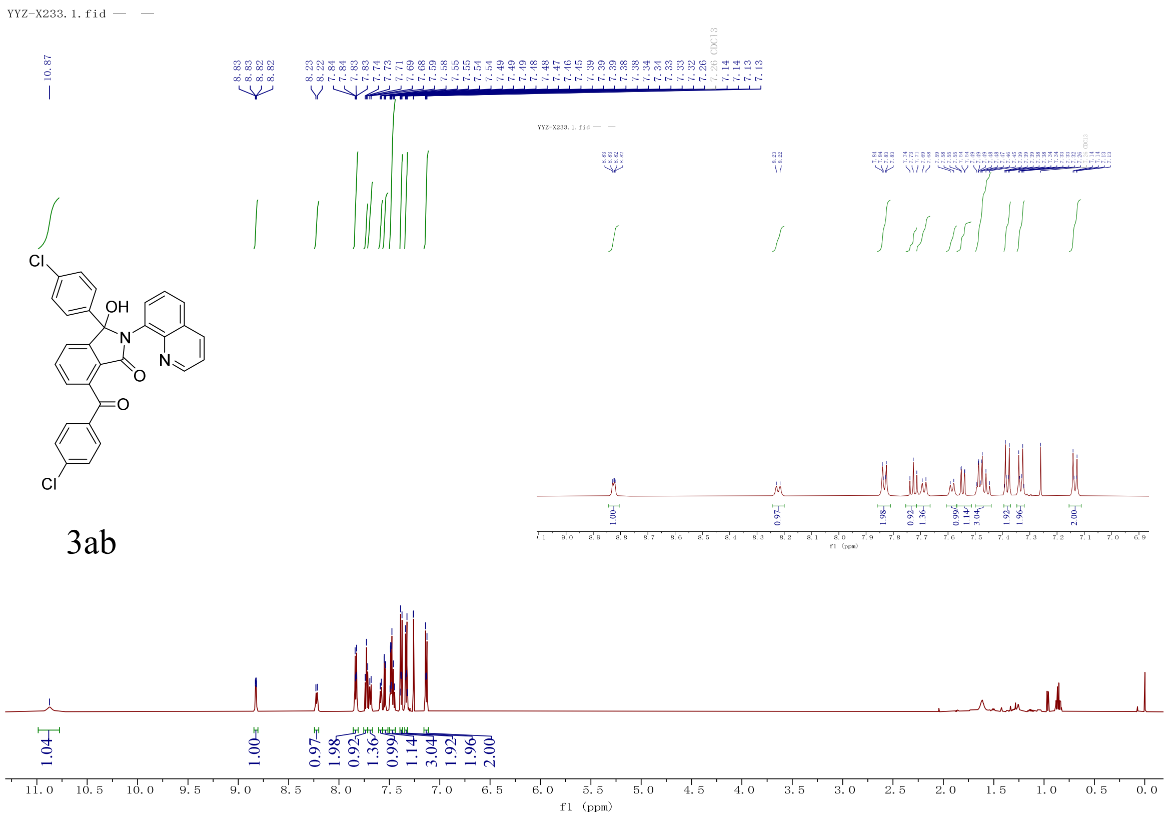
YYZ-X233.1.fid
10.87
8.83
8.83
8.82
8.82
8.23
8.22
7.84
7.83
7.83
7.74
7.73
7.71
7.69
7.68
7.59
7.58
7.55
7.55
7.54
7.54
7.49
7.49
7.48
7.48
7.47
7.46
7.45
7.39
7.39
7.38
7.38
7.34
7.34
7.33
7.33
7.32
7.26
7.26 CDCl3
7.14
7.14
7.13
7.13
1.04
1.00
0.97
1.98
0.92
1.36
0.99
1.14
3.04
1.92
1.96
2.00
f1 (ppm)

3ab

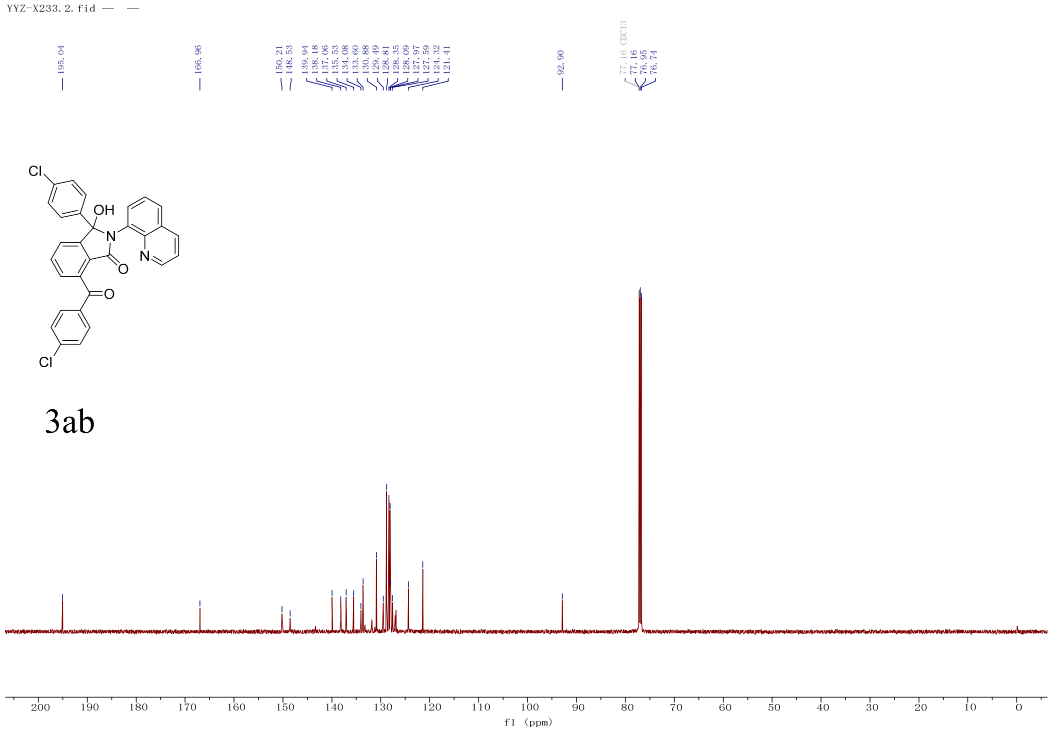
YYZ-X233.2.fid
195.04
166.96
150.21
148.53
139.94
138.18
137.06
135.53
134.08
133.60
130.88
129.49
128.81
128.35
128.09
127.97
127.59
124.32
121.41
92.90
77.16 CDCl3
77.16
76.95
76.74
f1 (ppm)

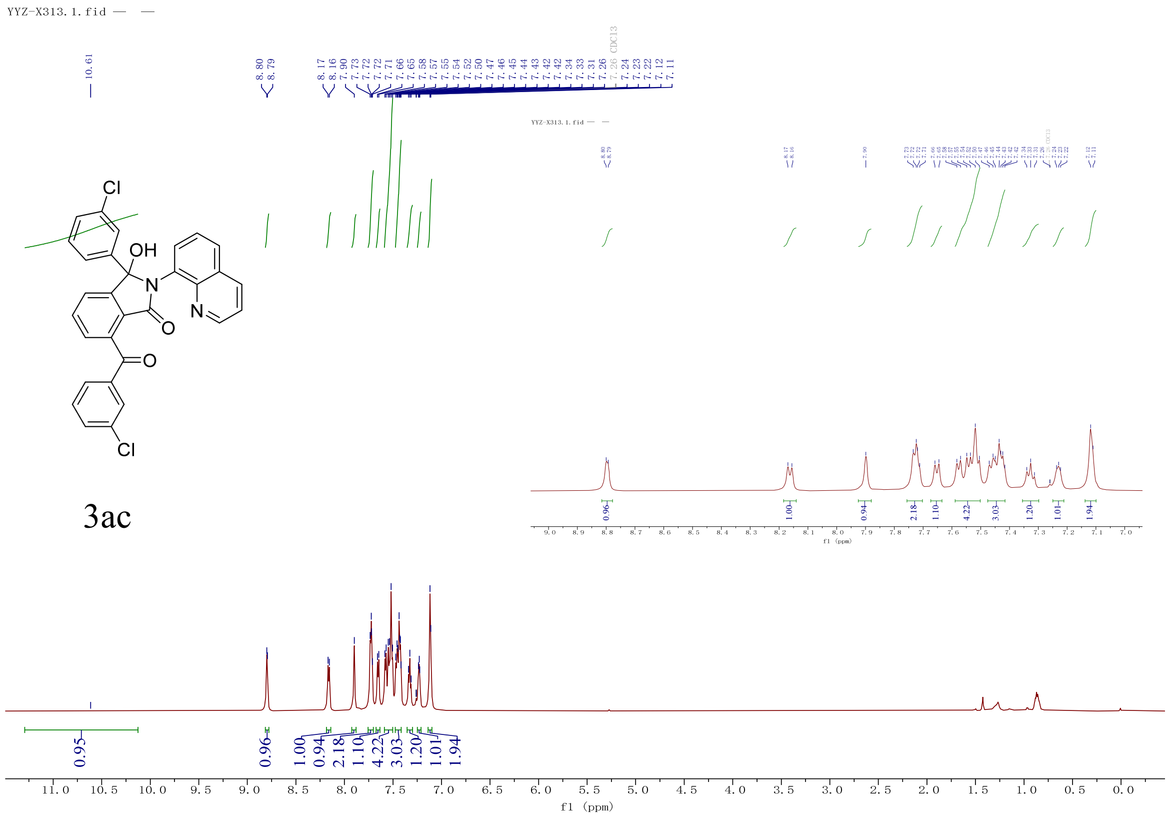
YYZ-X313.1.fid
10.61
8.80 8.79
8.17 8.16 7.90 7.73 7.72 7.72 7.71 7.66 7.65 7.58 7.57 7.55 7.54 7.52 7.50 7.47 7.46 7.45 7.44 7.43 7.42 7.42 7.34 7.33 7.31 7.26 CDCl3 7.24 7.23 7.22 7.12 7.11
Cl
OH
N
N
O
O
Cl
3ac
0.95
0.96
1.00
0.94
2.18
1.10
4.22
3.03
1.20
1.01
1.94
f1 (ppm)

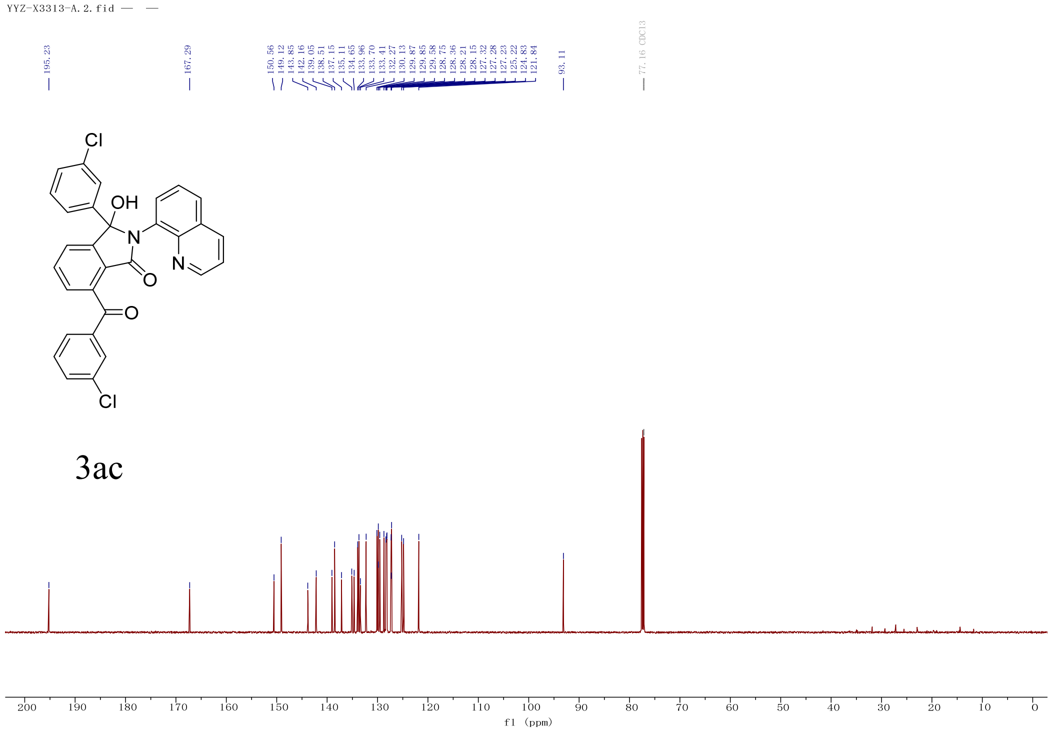
YYZ-X3313-A.2.fid
195.23
167.29
150.56 149.12 143.85 142.16 139.05 138.51 137.15 135.11 134.65 133.96 133.70 133.41 132.27 130.13 129.87 129.85 129.58 128.75 128.36 128.21 128.15 127.32 127.28 127.23 125.22 124.83 121.84
93.11
77.16 CDCl3
Cl
OH
N
N
O
O
Cl
3ac
f1 (ppm)

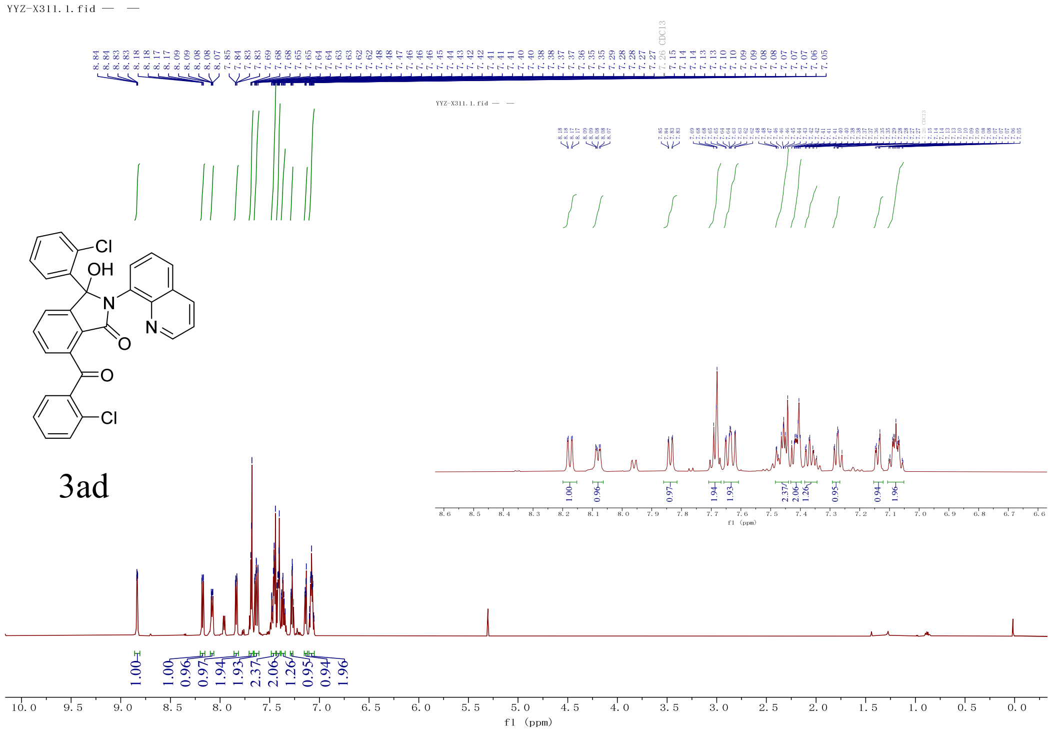
YYZ-X311.1.fid — —
8.84 8.84 8.83 8.83 8.18 8.18 8.17 8.17 8.09 8.09 8.08 8.08 8.07 7.85 7.84 7.83 7.83 7.69 7.68 7.68 7.65 7.65 7.64 7.64 7.63 7.63 7.62 7.62 7.48 7.48 7.47 7.46 7.46 7.45 7.44 7.43 7.42 7.42 7.41 7.41 7.41 7.40 7.40 7.38 7.38 7.37 7.37 7.36 7.35 7.35 7.29 7.28 7.28 7.27 7.27 7.26 CDCl3 7.15 7.14 7.14 7.13 7.13 7.10 7.10 7.09 7.09 7.08 7.08 7.07 7.07 7.07 7.06 7.05
Cl
OH
N
N
O
O
Cl
3ad
1.00 0.96 0.97 1.94 1.93 2.37 2.06 1.26 0.95 0.94 1.96
f1 (ppm)

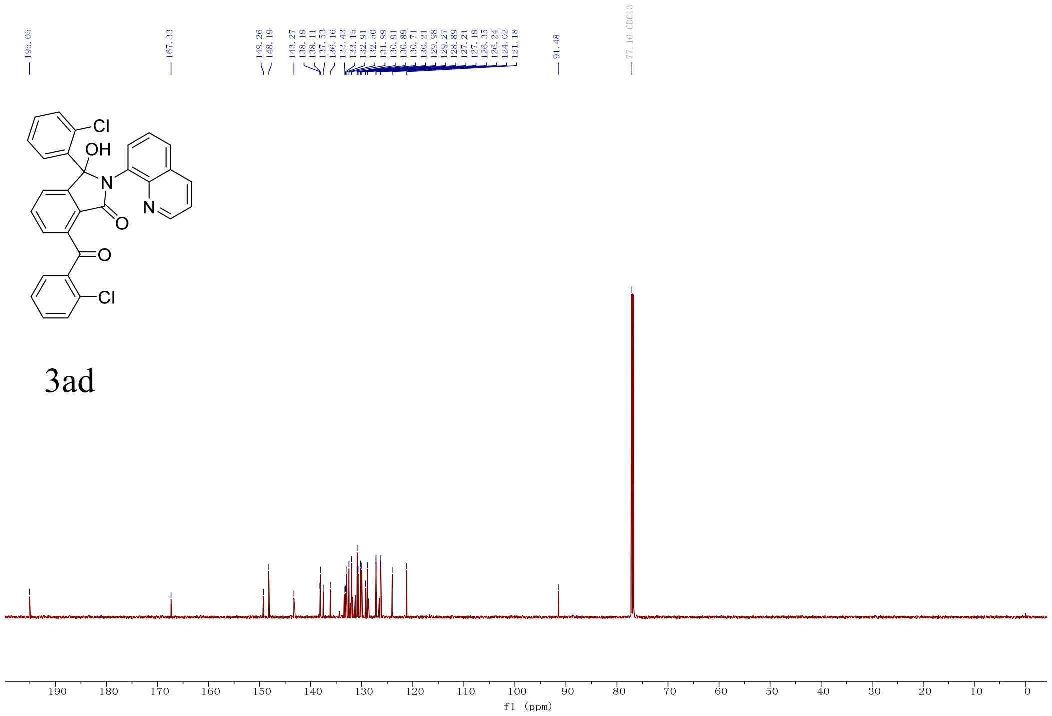
YYZ-X311-A.2.fid — —
195.05
167.33
149.26 148.19
143.27 138.19 138.11 137.53 136.16 133.43 133.15 132.91 132.50 131.99 130.91 130.89 130.71 130.21 129.98 129.27 128.89 127.21 127.19 126.35 126.24 124.02 121.18
91.48
77.16 CDCl3
Cl
OH
N
N
O
O
Cl
3ad
f1 (ppm)

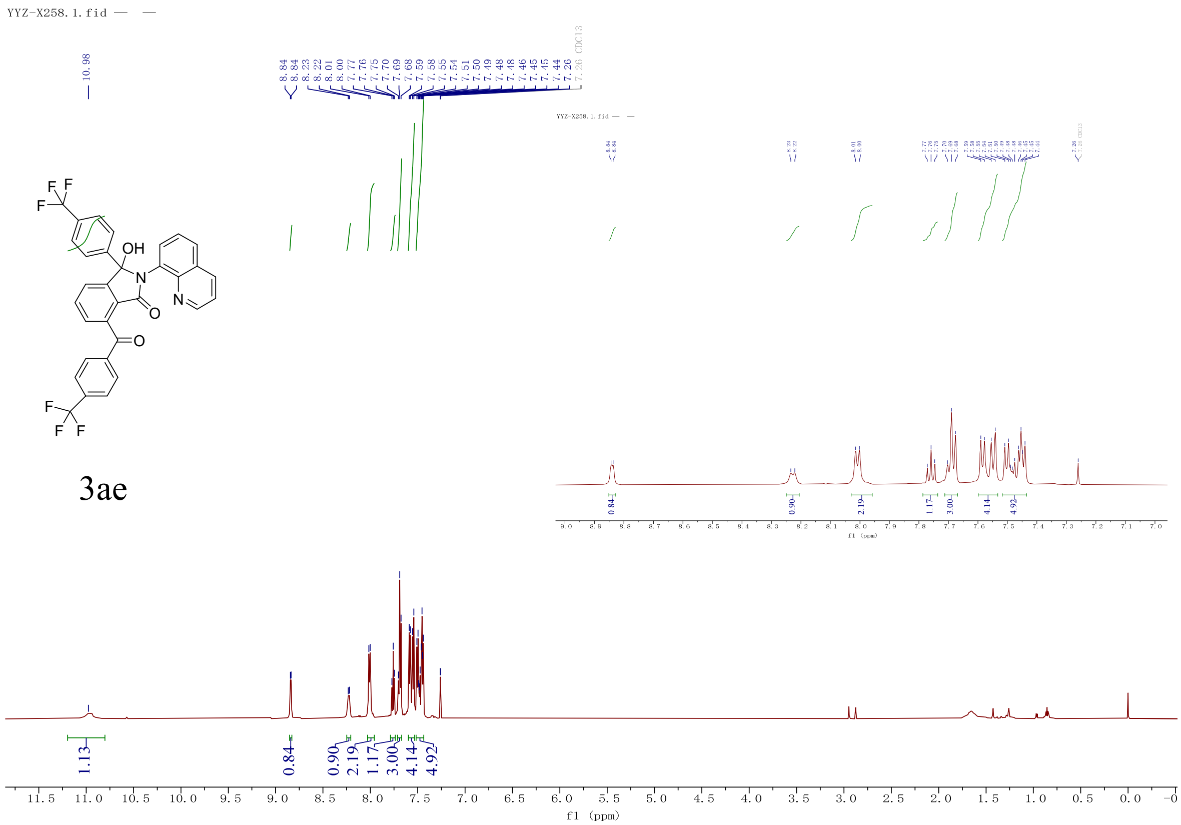
YYZ-X258.1.fid
10.98
8.84 8.84 8.23 8.22 8.01 8.00 7.77 7.76 7.75 7.70 7.69 7.68 7.59 7.58 7.55 7.54 7.51 7.50 7.49 7.48 7.46 7.45 7.45 7.44 7.26 7.26 CDCl3
F
F
F
OH
N
N
O
O
F
F
F
0.84
0.90
2.19
1.17
3.00
4.14
4.92
1.13
f1 (ppm)

3ae

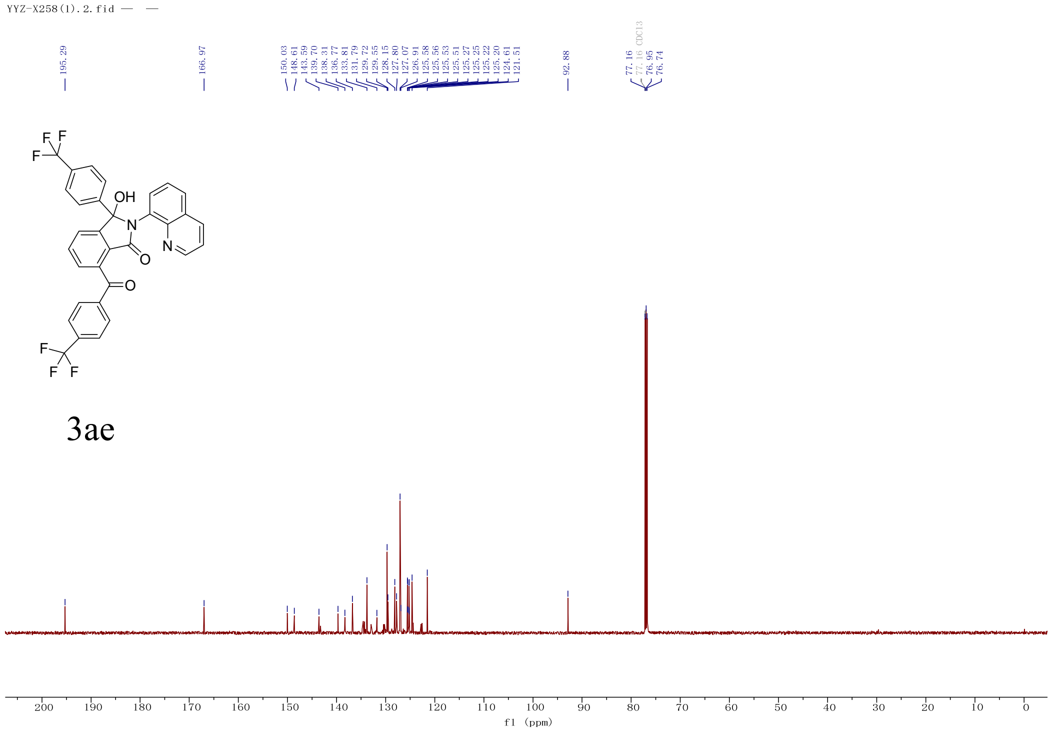
YYZ-X258(1).2.fid
195.29
166.97
150.03 148.61 143.59 139.70 138.31 136.37 133.81 131.79 129.72 129.55 128.15 127.80 127.07 126.94 125.58 125.56 125.53 125.51 125.27 125.25 125.22 125.20 124.61 121.51
92.88
77.16 77.16 CDCl3 76.95 76.74
F
F
F
OH
N
N
O
O
F
F
F
f1 (ppm)

3ae

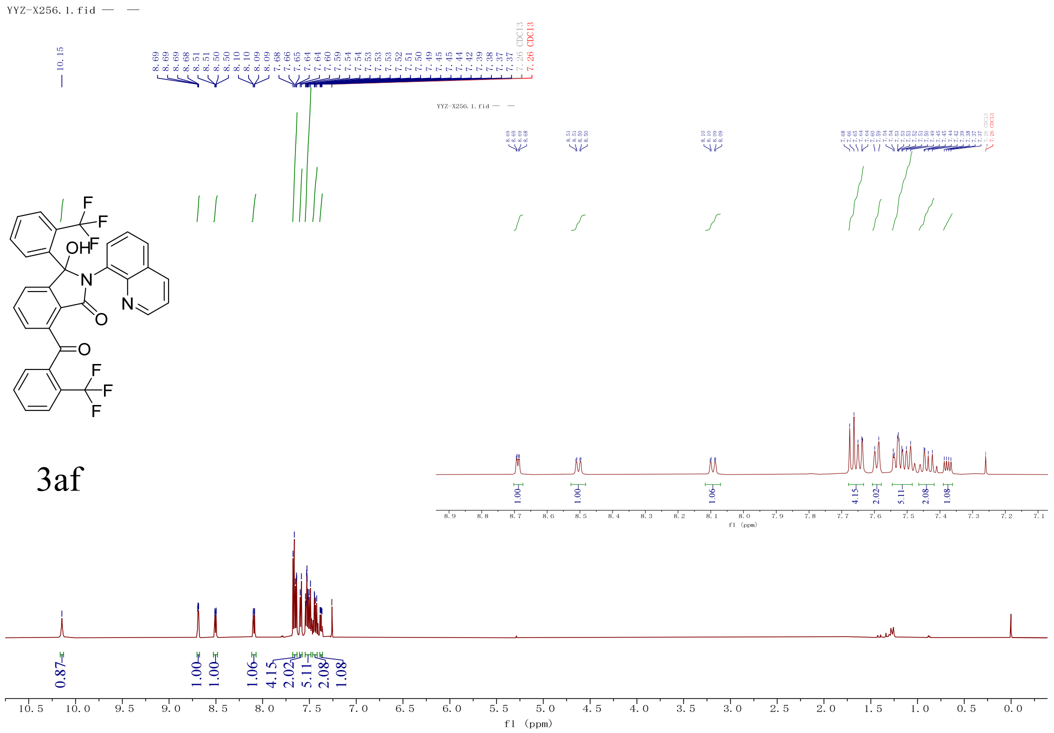

YYZ-X256.1.fid
10.15
8.69 8.69 8.69 8.68 8.51 8.51 8.50 8.50 8.10 8.10 8.09 8.09 7.68 7.66 7.65 7.64 7.64 7.60 7.59 7.54 7.54 7.53 7.53 7.53 7.52 7.51 7.50 7.49 7.45 7.45 7.44 7.42 7.39 7.38 7.37 7.37
7.26 CDCl3
7.26 CDCl3
0.87 1.00 1.00 1.06 4.15 2.02 5.11 2.08 1.08
10.5 10.0 9.5 9.0 8.5 8.0 7.5 7.0 6.5 6.0 5.5 5.0 4.5 4.0 3.5 3.0 2.5 2.0 1.5 1.0 0.5 0.0
f1 (ppm)

3af

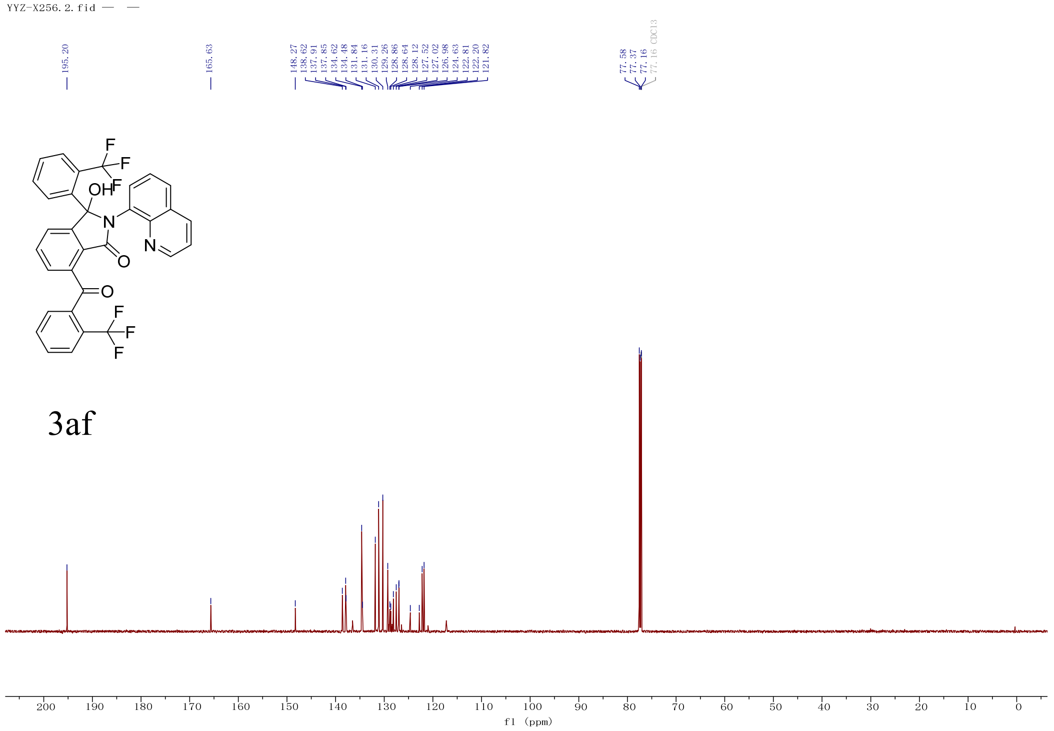

YYZ-X256.2.fid
195.20
165.63
148.27 138.62 137.91 137.85 134.62 134.48 131.84 131.16 130.31 129.26 128.86 128.64 128.12 127.52 127.02 126.98 124.63 122.81 122.20 121.82
77.58 77.37 77.16
77.16 CDCl3
200 190 180 170 160 150 140 130 120 110 100 90 80 70 60 50 40 30 20 10 0
f1 (ppm)

3af

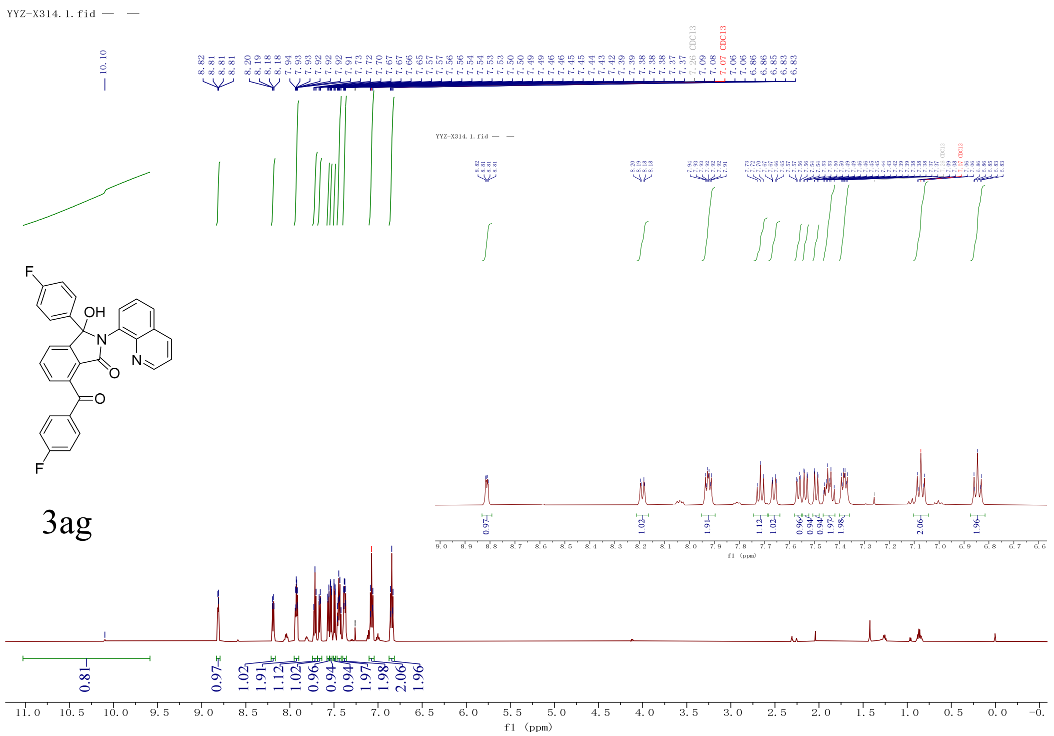

YYZ-X314.1.fid
10.10
8.82 8.81 8.81 8.81 8.20 8.19 8.18 8.18 7.94 7.93 7.93 7.92 7.92 7.92 7.91 7.91 7.73 7.72 7.70 7.67 7.67 7.66 7.65 7.57 7.57 7.56 7.56 7.54 7.54 7.53 7.53 7.50 7.50 7.49 7.49 7.46 7.46 7.45 7.45 7.44 7.43 7.42 7.39 7.39 7.38 7.38 7.38 7.37 7.37 7.26 CDCl3 7.09 7.08 7.07 CDCl3 7.06 7.06 6.86 6.86 6.85 6.83 6.83
0.81 0.97 1.02 1.91 1.12 1.02 0.96 0.94 0.94 1.97 1.98 2.06 1.96
f1 (ppm)

3ag

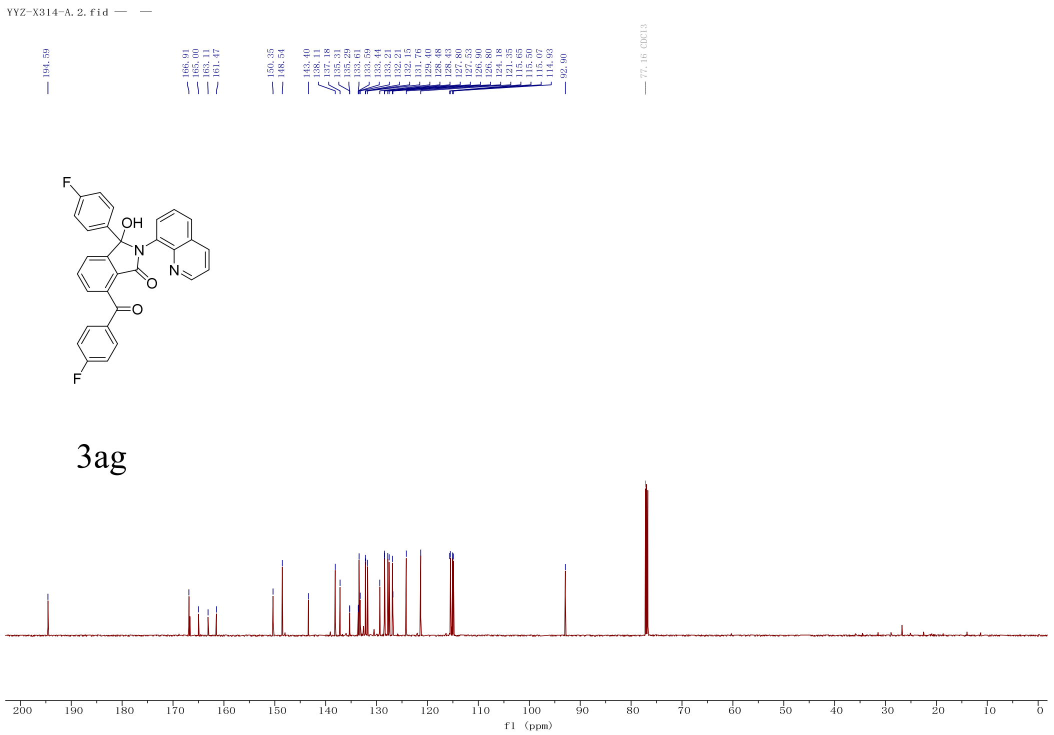

YYZ-X314-A.2.fid
194.59
166.91 165.00 163.11 161.47
150.35 148.54
143.40 138.11 137.18 135.31 135.29 133.61 133.59 133.44 133.21 132.21 132.15 131.76 129.40 128.48 128.43 127.80 127.53 126.90 126.80 124.18 121.35 115.65 115.50 115.07 114.93
92.90
77.16 CDCl3
f1 (ppm)

3ag

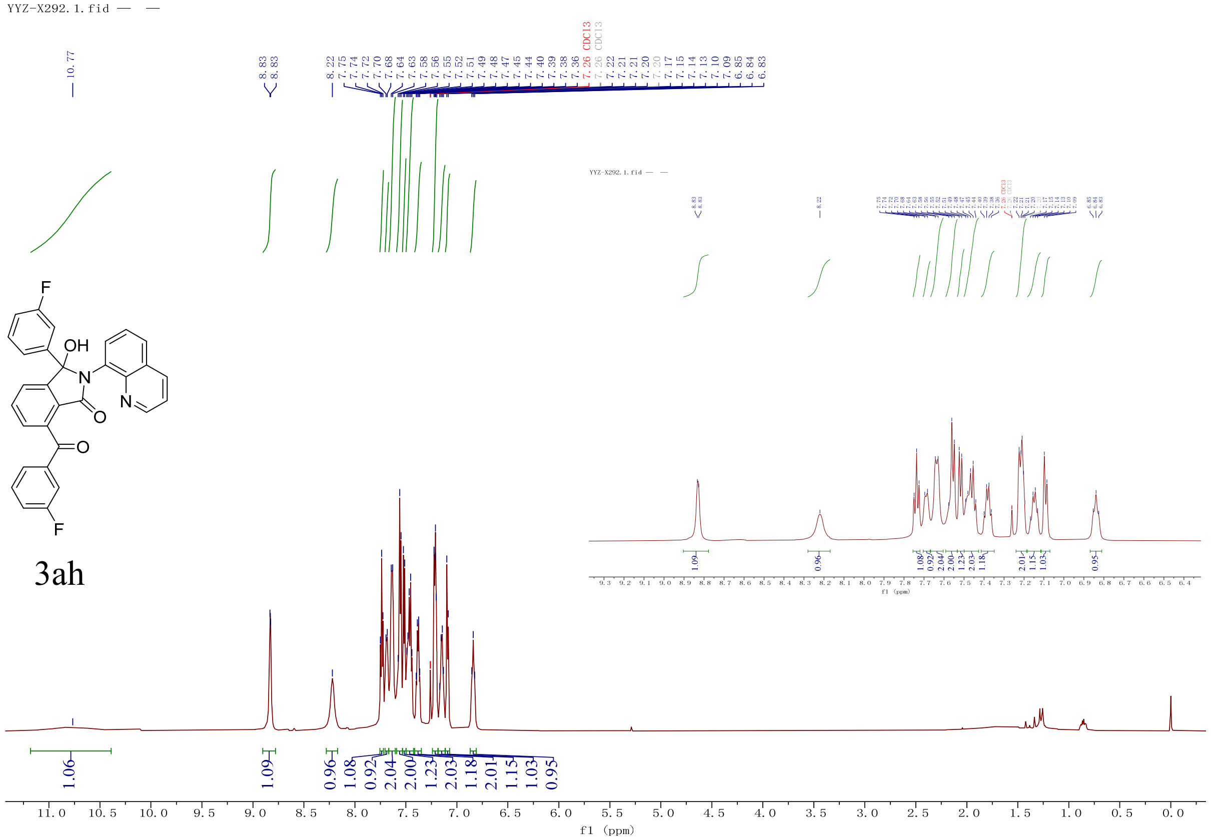
YYZ-X292.1.fid
10.77
8.83
8.83
8.22
7.75
7.74
7.72
7.70
7.68
7.64
7.63
7.58
7.56
7.55
7.52
7.51
7.49
7.48
7.47
7.45
7.44
7.40
7.39
7.38
7.36
7.26 CDCl3
7.26 CDCl3
7.22
7.21
7.21
7.20
7.20
7.17
7.15
7.14
7.13
7.10
7.09
6.85
6.84
6.83
F
OH
N
N
O
O
F
1.06
1.09
0.96
1.08
0.92
2.04
2.00
1.23
2.03
1.18
2.01
1.15
1.03
0.95
f1 (ppm)

3ah

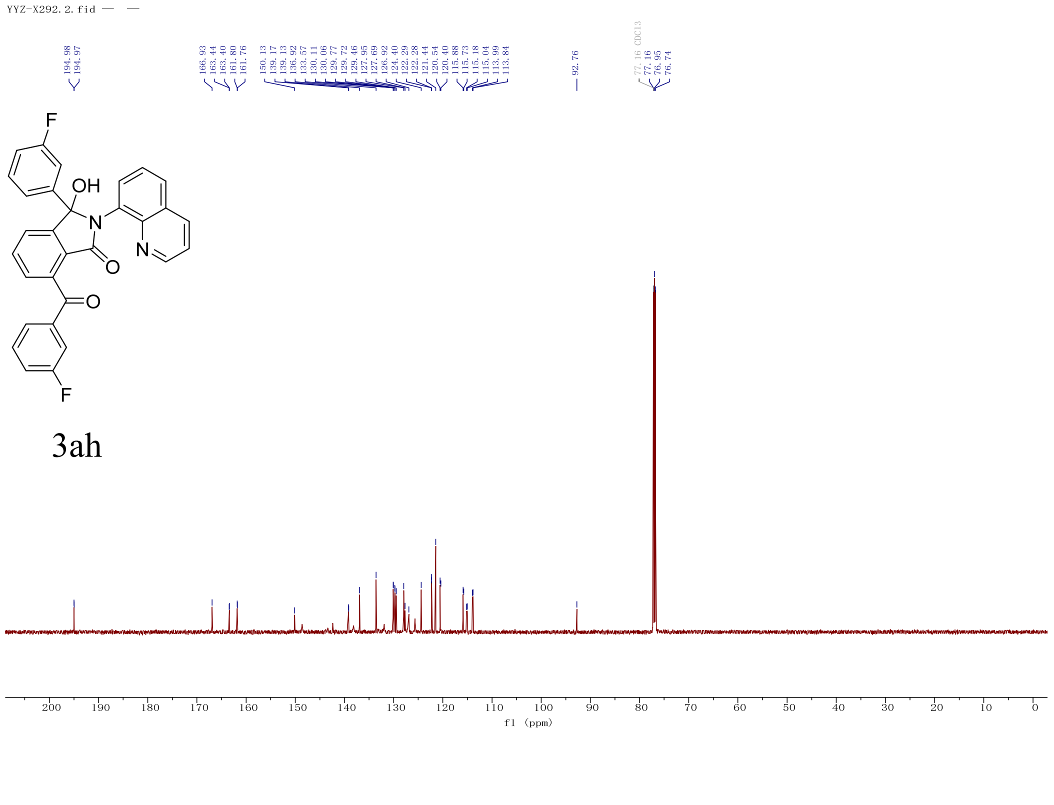
YYZ-X292.2.fid
194.98
194.97
166.93
163.44
163.40
161.80
161.76
150.13
139.17
139.13
136.92
133.57
130.11
130.06
129.77
129.72
129.46
127.95
127.69
126.92
124.40
122.29
122.28
121.41
120.54
120.40
115.88
115.73
115.18
115.04
113.99
113.84
92.76
77.16 CDCl3
77.16
76.95
76.74
F
OH
N
N
O
O
F
f1 (ppm)

3ah

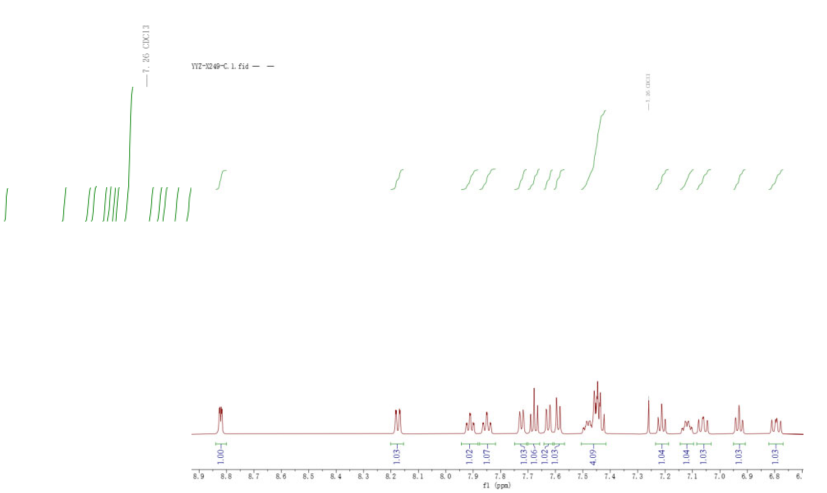

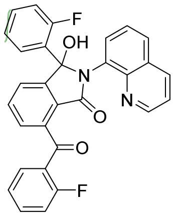

3ai

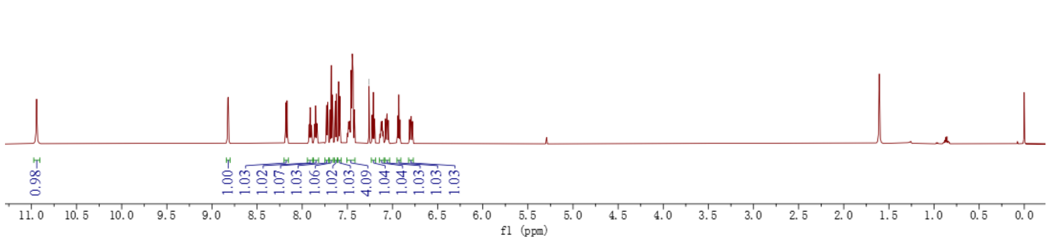

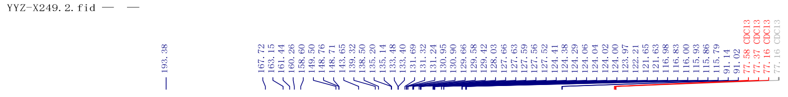

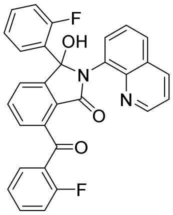

3ai

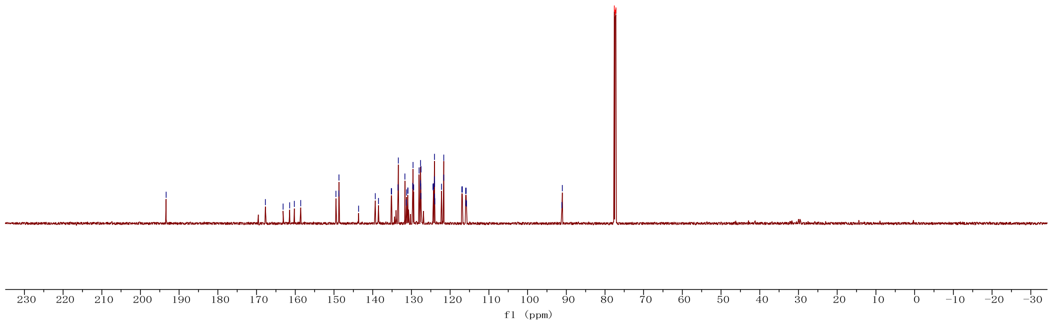

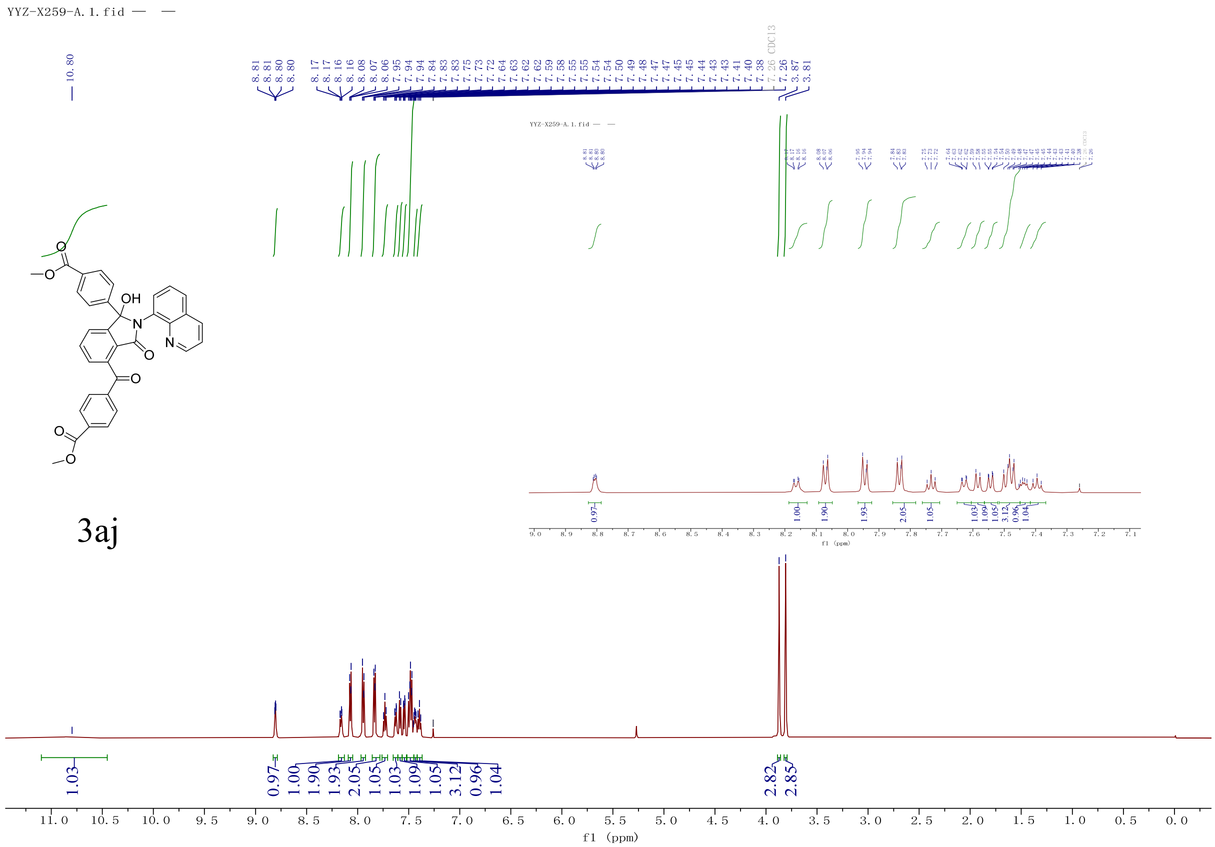

3aj

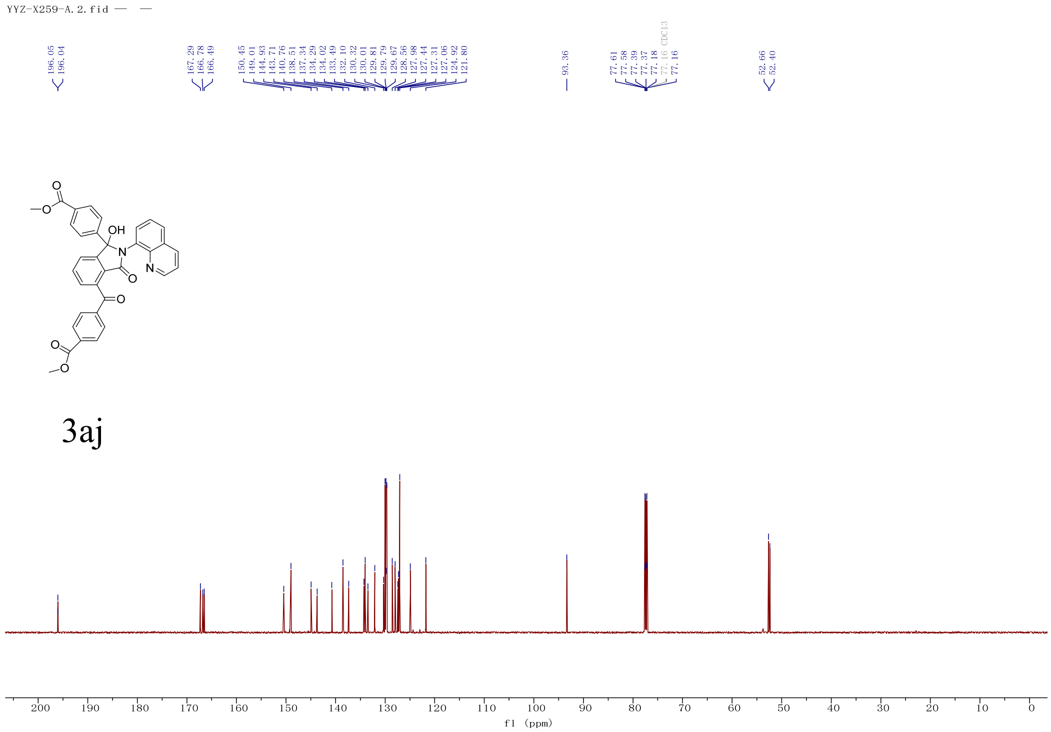

3aj

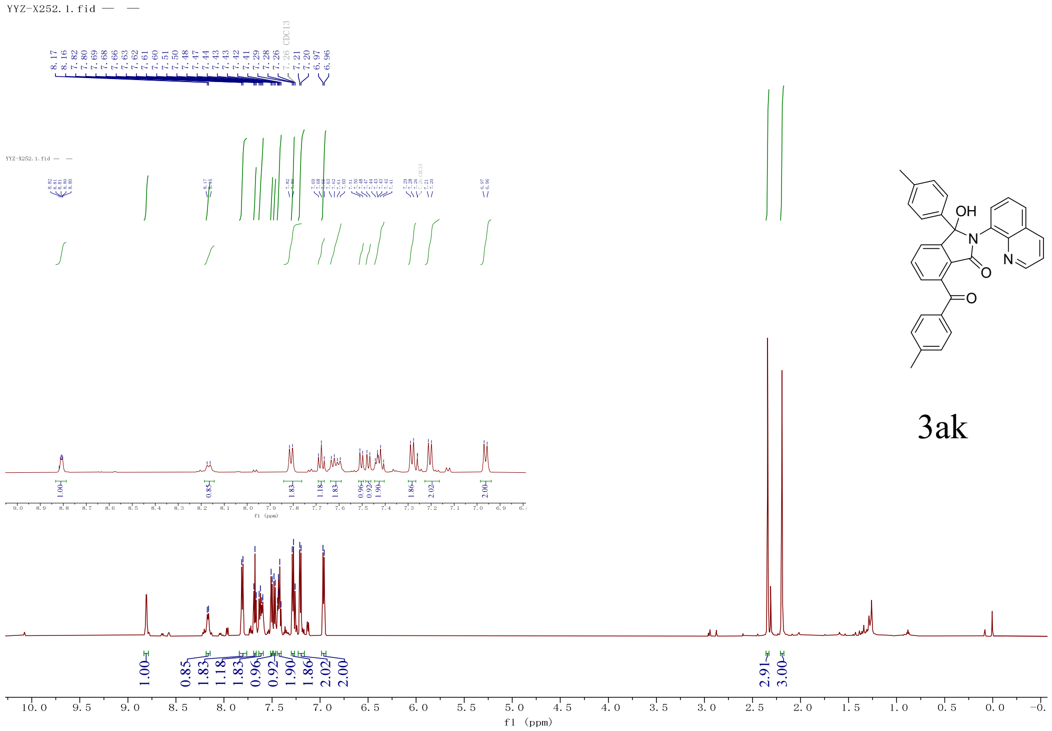
YYZ-X252.1.fid — —
8.17
8.16
7.82
7.80
7.69
7.68
7.66
7.63
7.62
7.61
7.60
7.51
7.50
7.48
7.47
7.44
7.43
7.43
7.42
7.41
7.29
7.28
7.26 CDCl3
7.21
7.20
6.97
6.96
1.00
0.85
1.83
1.18
1.83
0.96
0.92
1.90
1.86
2.02
2.00
2.91
3.00
f1 (ppm)

3ak

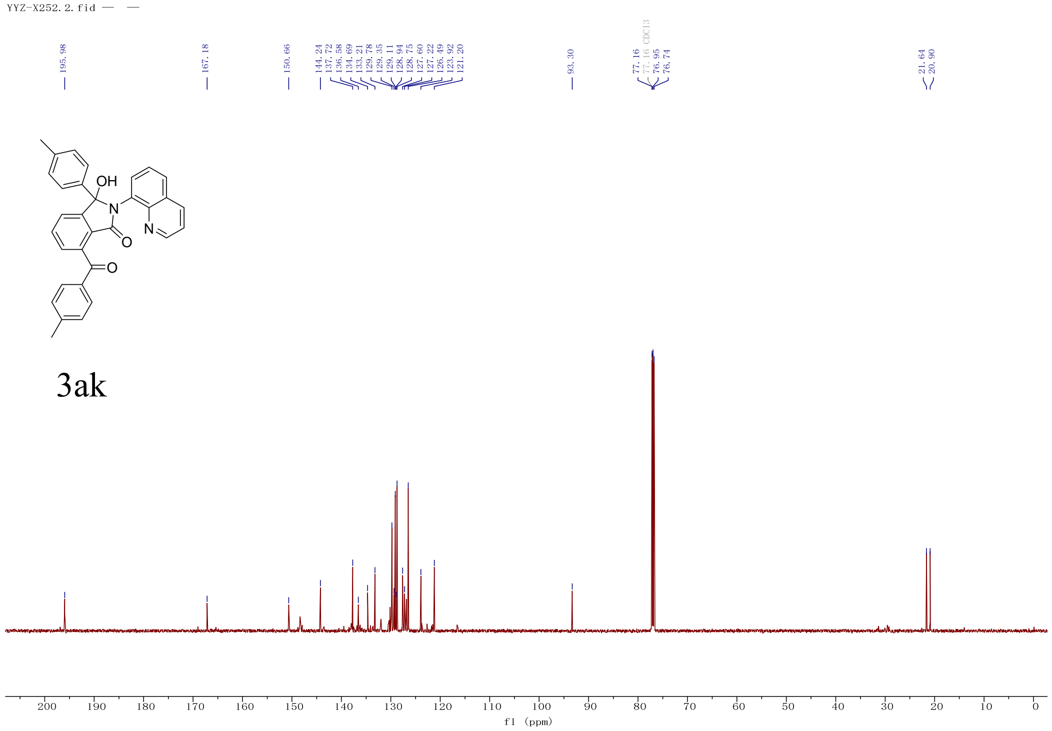
YYZ-X252.2.fid — —
195.98
167.18
150.66
144.24
137.72
136.58
134.69
133.21
129.78
129.35
129.11
128.94
128.75
127.60
127.22
126.49
123.92
121.20
93.30
77.16
77.16 CDCl3
76.95
76.74
21.64
20.90
f1 (ppm)

3ak

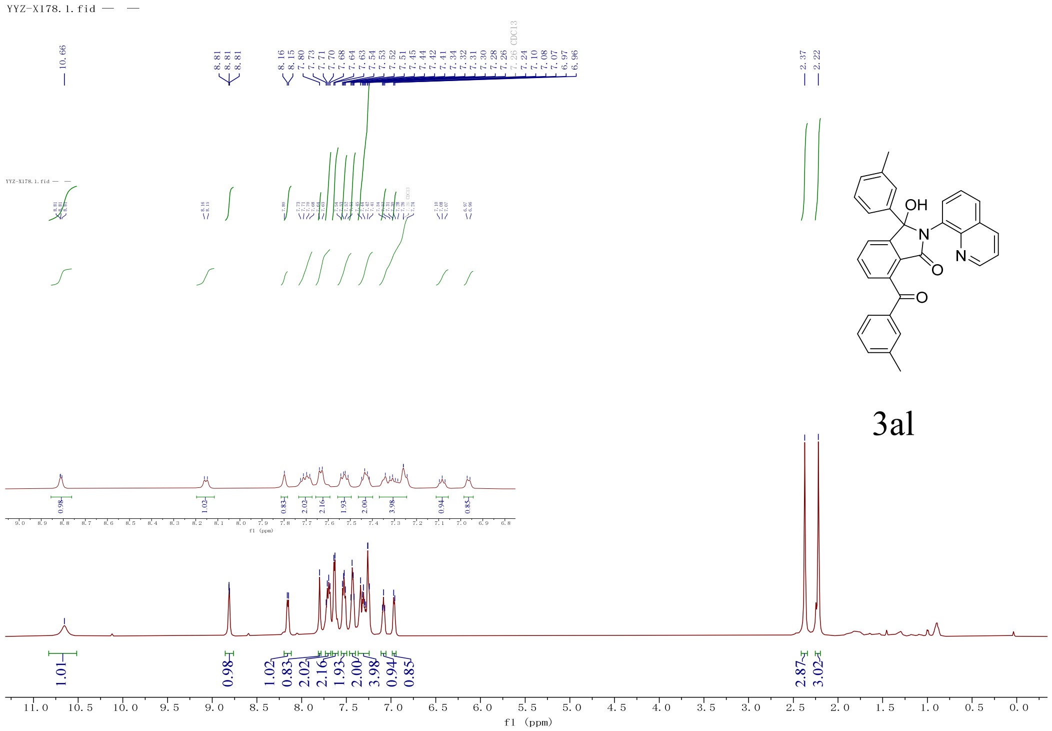
YYZ-X178.1.fid
10.66
8.81
8.81
8.81
8.16
8.15
7.80
7.73
7.71
7.70
7.68
7.64
7.63
7.54
7.53
7.52
7.51
7.45
7.44
7.42
7.41
7.34
7.32
7.31
7.30
7.28
7.26
7.26 CDCl3
7.24
7.10
7.08
7.07
6.97
6.96
2.37
2.22
OH
N
O
N
O
3al
1.01
0.98
1.02
0.83
2.02
2.16
1.93
2.00
3.98
0.94
0.85
2.87
3.02
f1 (ppm)

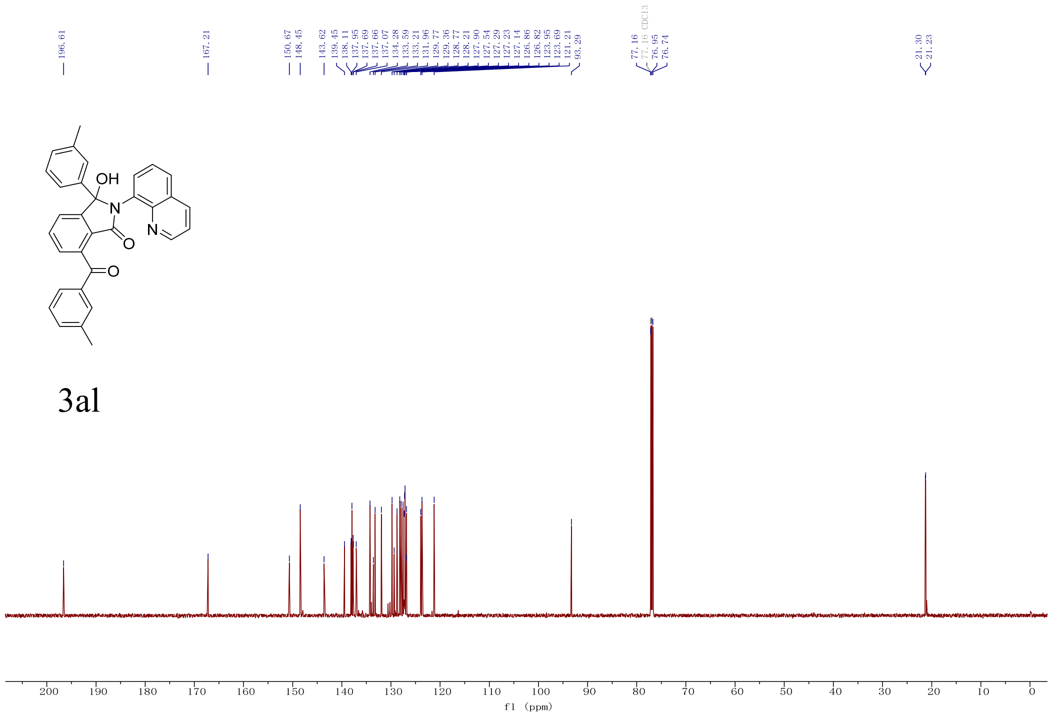
YYZ-X178(1).2.fid
196.61
167.21
150.67
148.45
143.62
139.45
138.11
137.95
137.69
137.66
137.07
134.28
133.59
133.21
131.96
129.77
129.36
128.77
128.21
127.90
127.54
127.29
127.23
127.14
126.86
126.82
123.95
123.69
121.21
93.29
77.16
77.16 CDCl3
76.95
76.74
21.30
21.23
OH
N
O
N
O
3al
f1 (ppm)

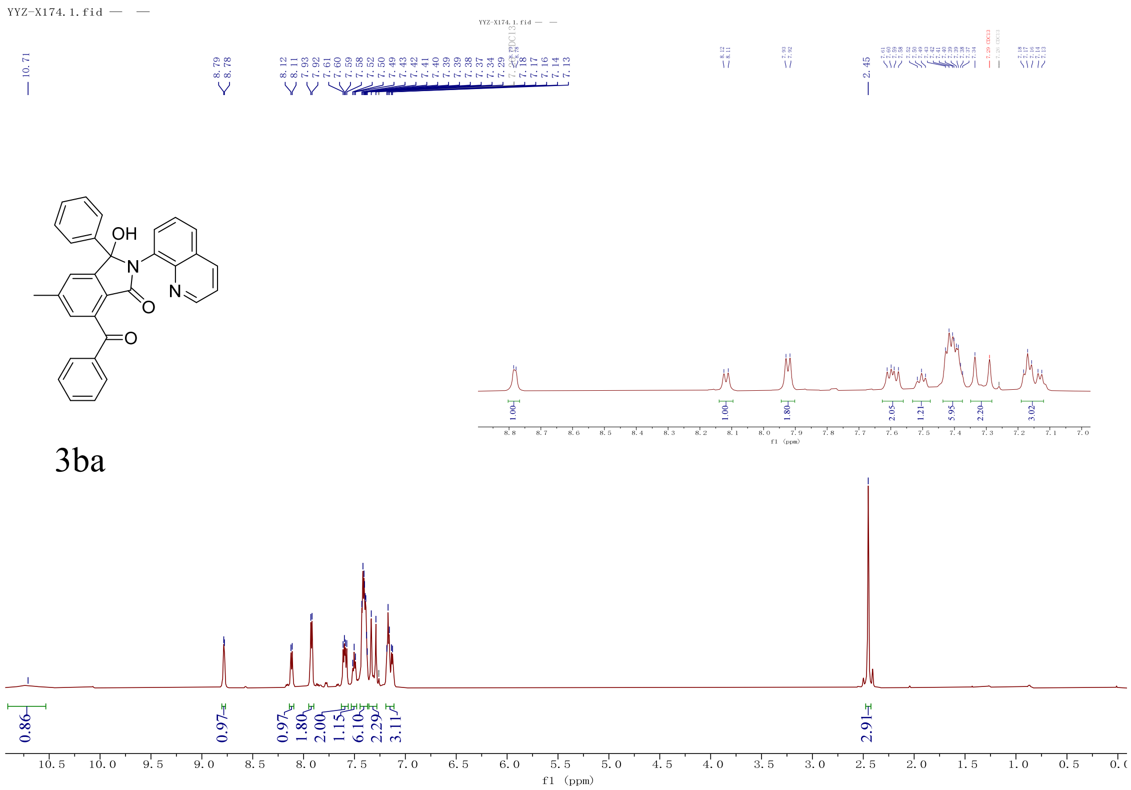
YYZ-X174.1.fid — —
10.71
8.79
8.78
8.12
8.11
7.93
7.92
7.61
7.60
7.59
7.58
7.52
7.50
7.49
7.43
7.42
7.41
7.40
7.39
7.39
7.38
7.37
7.34
7.29
7.18
7.17
7.16
7.14
7.13
2.45
OH
N
N
O
O
3ba
0.86
0.97
0.97
1.80
2.00
1.15
6.10
2.29
3.11
2.91
f1 (ppm)

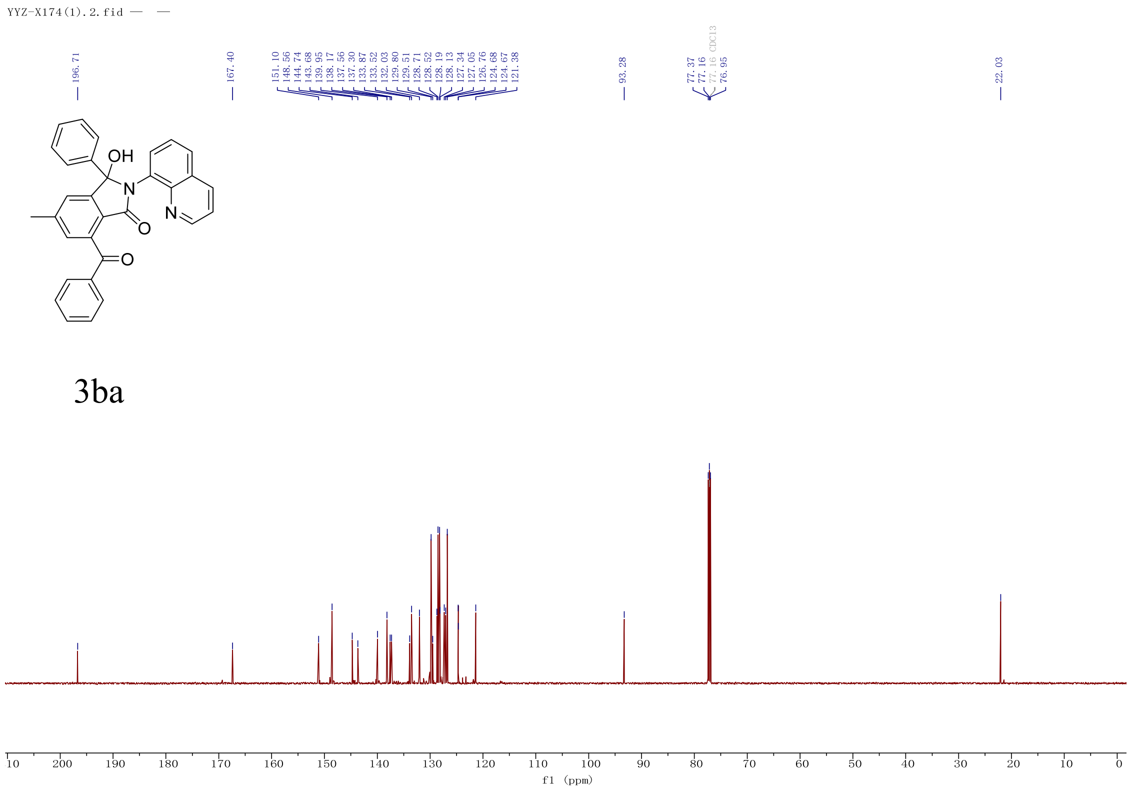
YYZ-X174(1).2.fid — —
196.71
167.40
151.10
148.56
144.74
143.68
139.95
138.17
137.56
137.30
133.87
133.52
132.03
129.80
129.51
128.71
128.52
128.19
128.13
127.34
127.05
126.76
124.68
124.67
121.38
93.28
77.37
77.16 CDCl3
76.95
22.03
OH
N
N
O
O
3ba
f1 (ppm)

YYZ-X261.1.fid — —

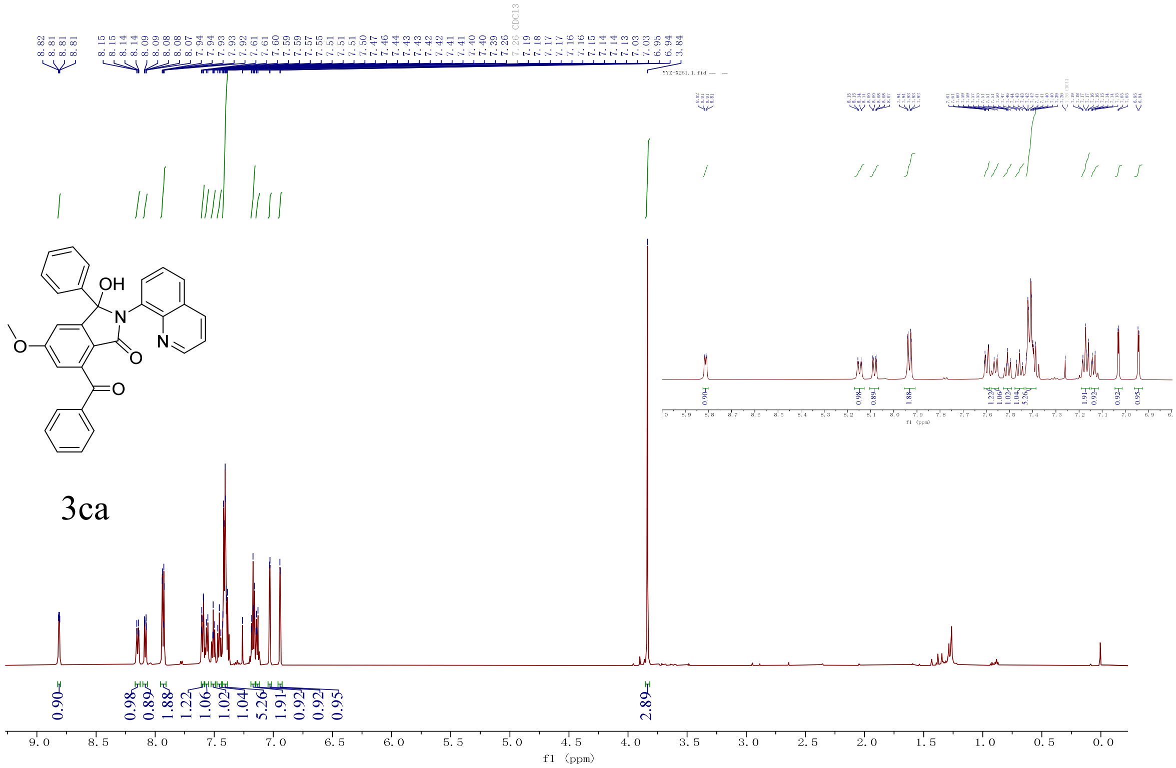

YYZ-X261(1).2.fid — —

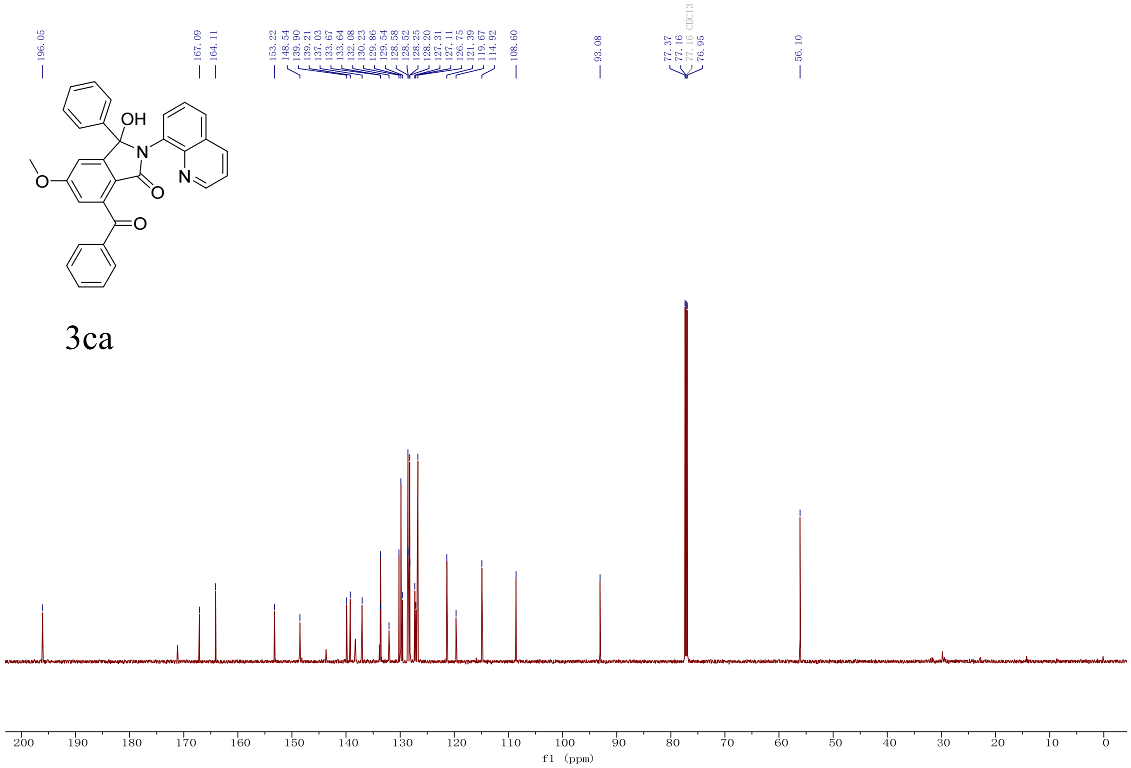

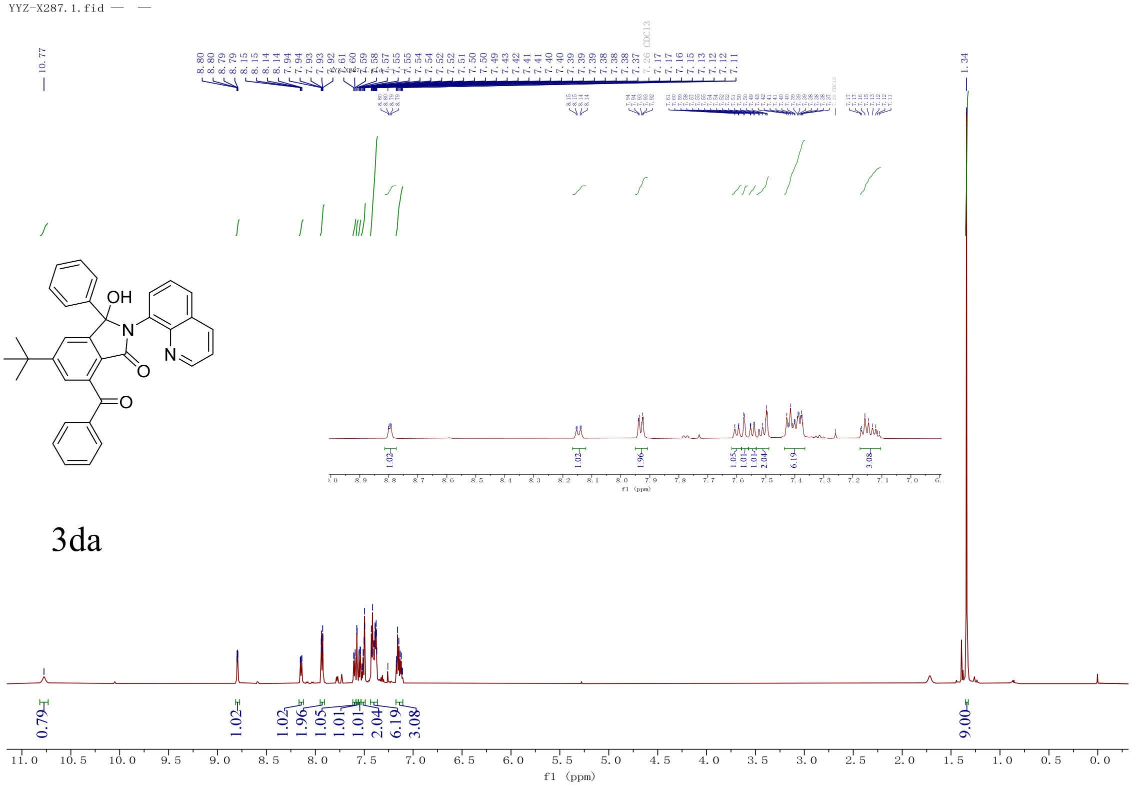
YYZ-X287.1.fid
10.77
8.80 8.80 8.79 8.79 8.15 8.15 8.14 8.14 7.94 7.94 7.93 7.93 7.92 7.61 7.60 7.59 7.58 7.57 7.55 7.55 7.54 7.54 7.52 7.52 7.51 7.50 7.50 7.49 7.43 7.42 7.41 7.41 7.40 7.40 7.39 7.39 7.39 7.38 7.38 7.38 7.37 7.26 CDCl3 7.17 7.17 7.16 7.15 7.13 7.13 7.12 7.11
1.34
OH
N
N
O
O
3da
0.79
1.02
1.02
1.96
1.05
1.01
1.01
2.04
6.19
3.08
9.00
f1 (ppm)

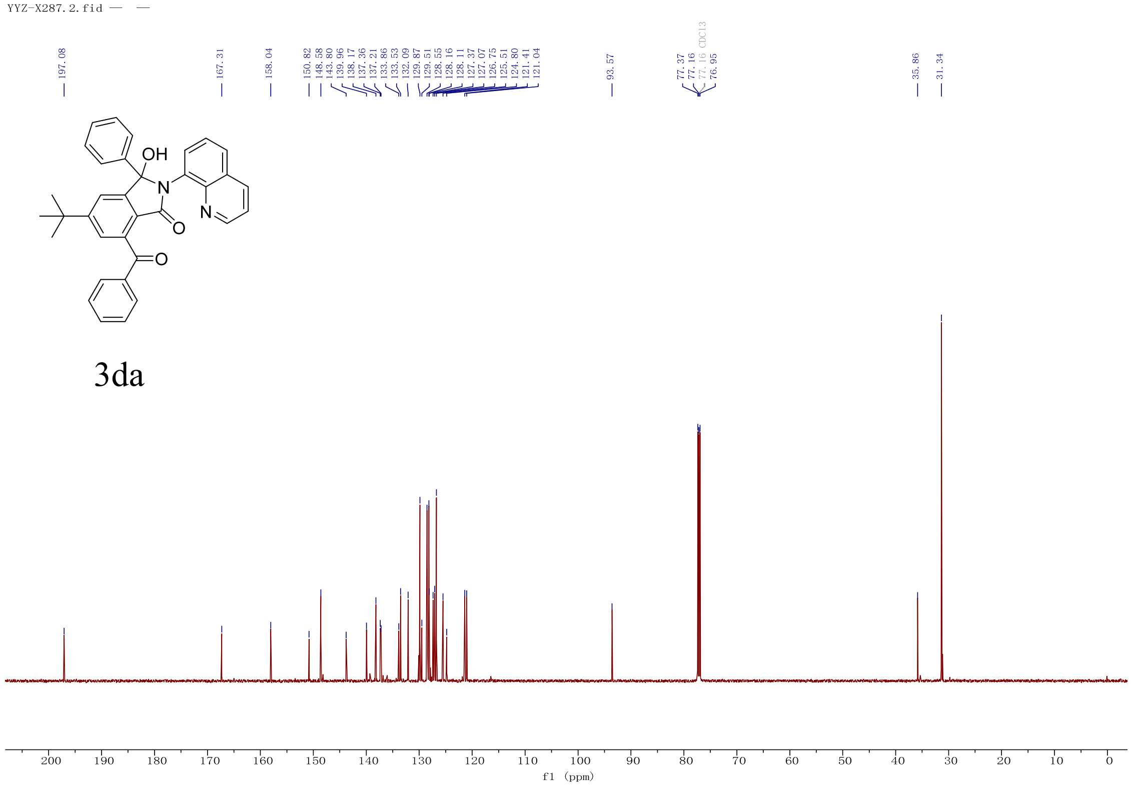
YYZ-X287.2.fid
197.08
167.31
158.04
150.82 148.58 143.80 139.96 138.17 137.36 137.21 133.86 133.53 132.09 129.87 129.51 128.55 128.16 128.11 127.37 127.07 126.75 125.51 124.80 121.41 121.04
93.57
77.37 77.16 CDCl3 76.95
35.86
31.34
OH
N
N
O
O
3da
f1 (ppm)

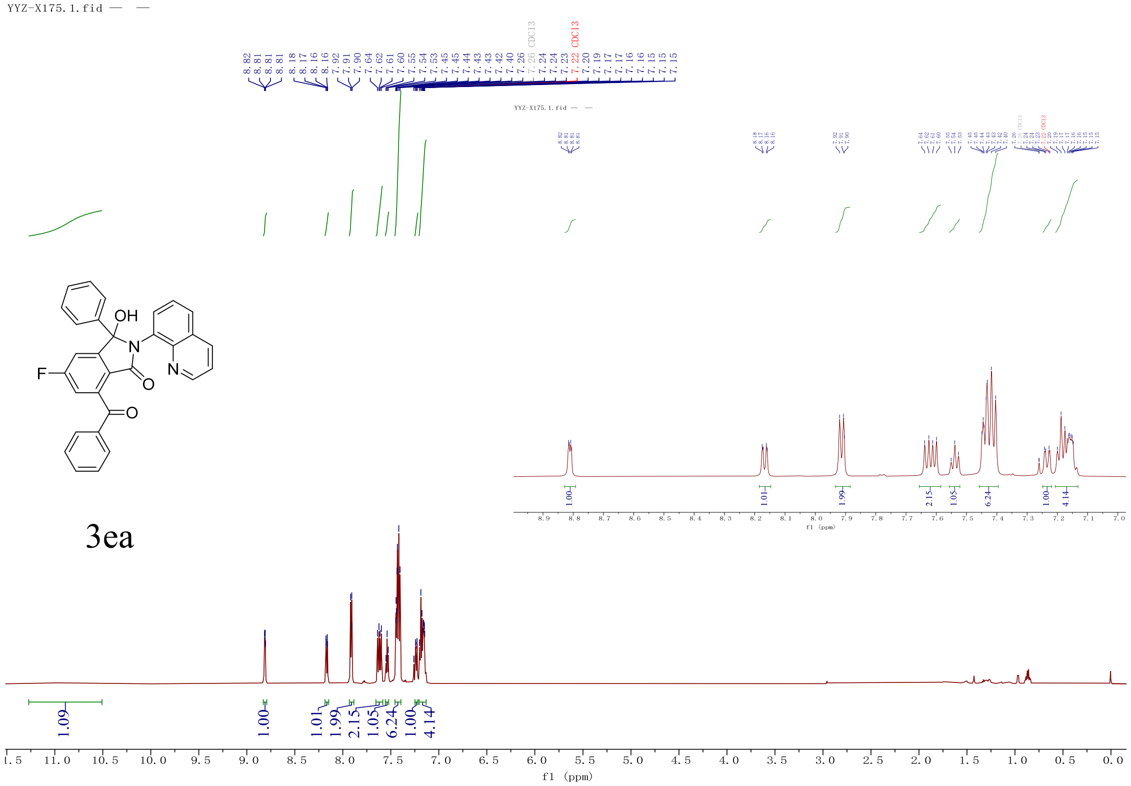
YYZ-X175.1.fid — —
8.82 8.81 8.81 8.81 8.18 8.17 8.16 8.16 7.92 7.91 7.90 7.64 7.62 7.61 7.60 7.55 7.54 7.53 7.45 7.45 7.44 7.43 7.43 7.42 7.40 7.26 CDCl3 7.24 7.24 7.23 7.22 CDCl3 7.20 7.19 7.17 7.17 7.16 7.16 7.15 7.15
1.09 1.00 1.01 1.99 2.15 1.05 6.24 1.00 4.14
f1 (ppm)

3ea

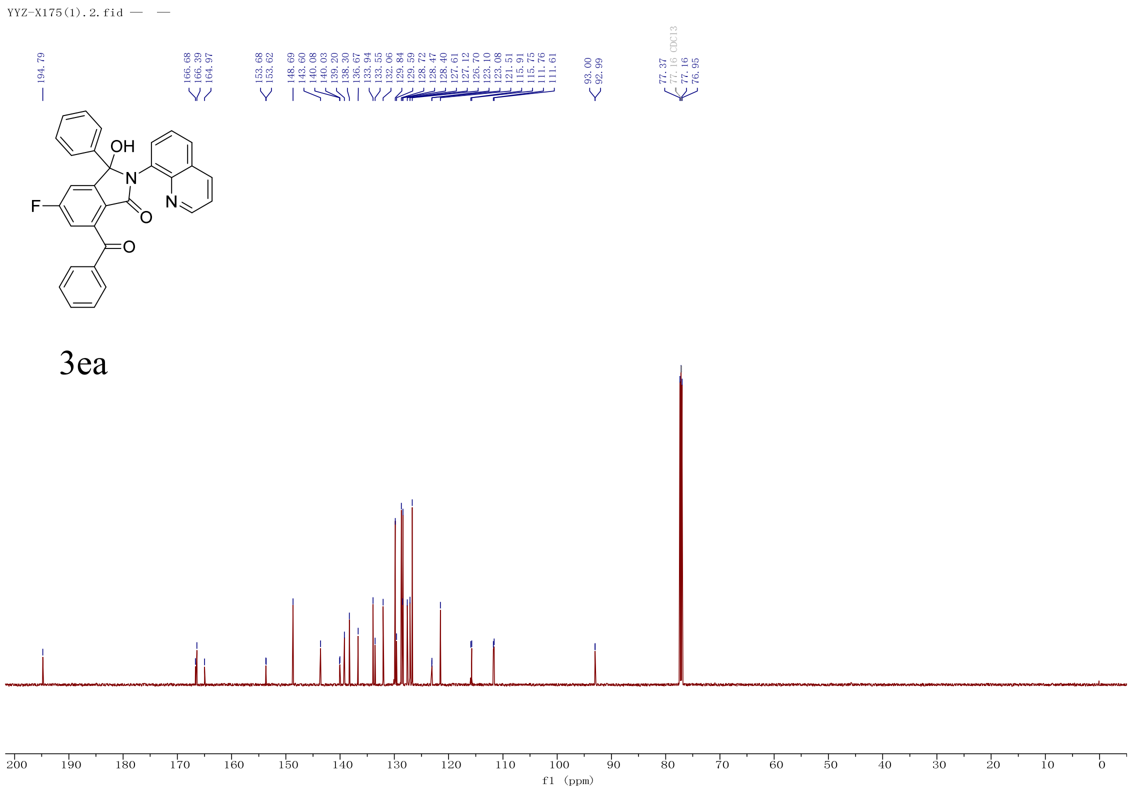
YYZ-X175(1).2.fid — —
194.79
166.68 166.39 164.97
153.68 153.62
148.69 143.60 140.08 140.03 139.20 138.30 136.67 133.94 133.55 132.06 129.84 129.59 128.72 128.47 128.40 127.61 127.12 126.70 123.10 123.08 121.51 115.91 115.75 111.76 111.61
93.00 92.99
77.37 77.16 CDCl3 76.95
f1 (ppm)

3ea

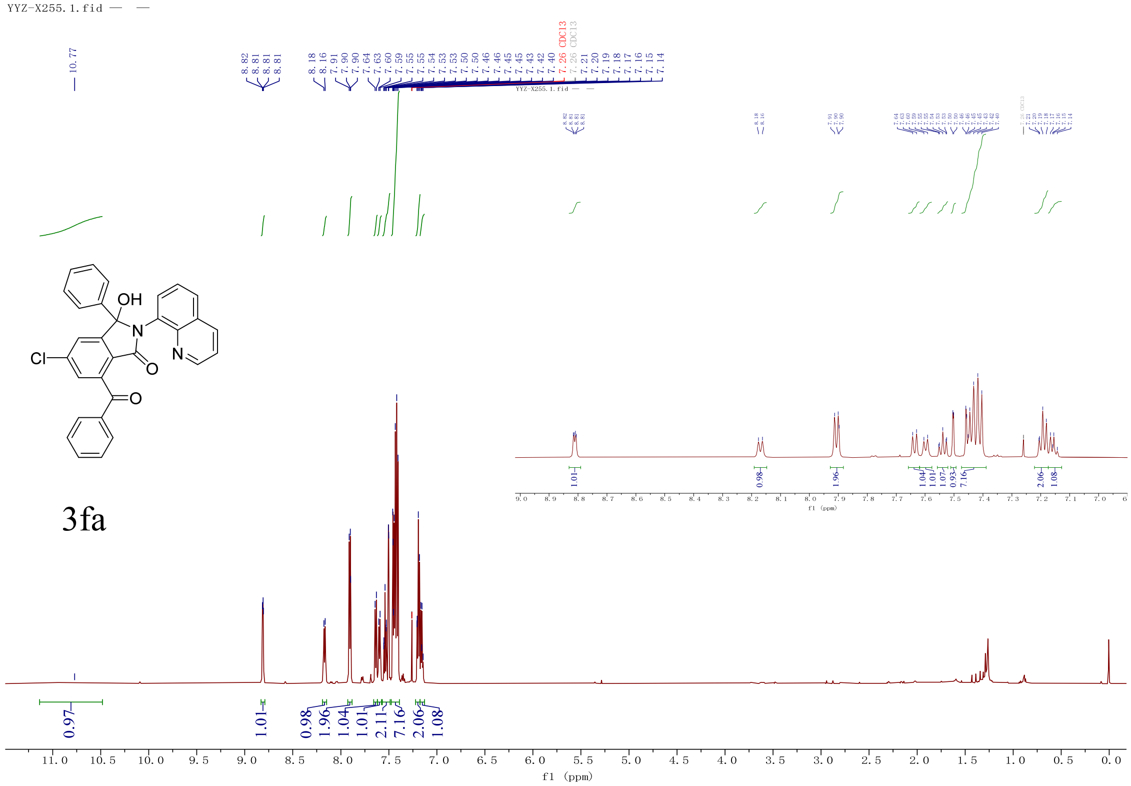

YYZ-X255.1.fid
10.77
8.82
8.81
8.81
8.81
8.18
8.16
7.91
7.90
7.90
7.64
7.63
7.60
7.59
7.55
7.55
7.54
7.53
7.53
7.50
7.50
7.46
7.46
7.45
7.43
7.42
7.40
7.26 CDCl3
7.26 CDCl3
7.21
7.20
7.19
7.18
7.17
7.16
7.15
7.14
OH
N
Cl
N
O
O
0.97
1.01
0.98
1.96
1.04
1.01
2.11
7.16
2.06
1.08
f1 (ppm)

3fa

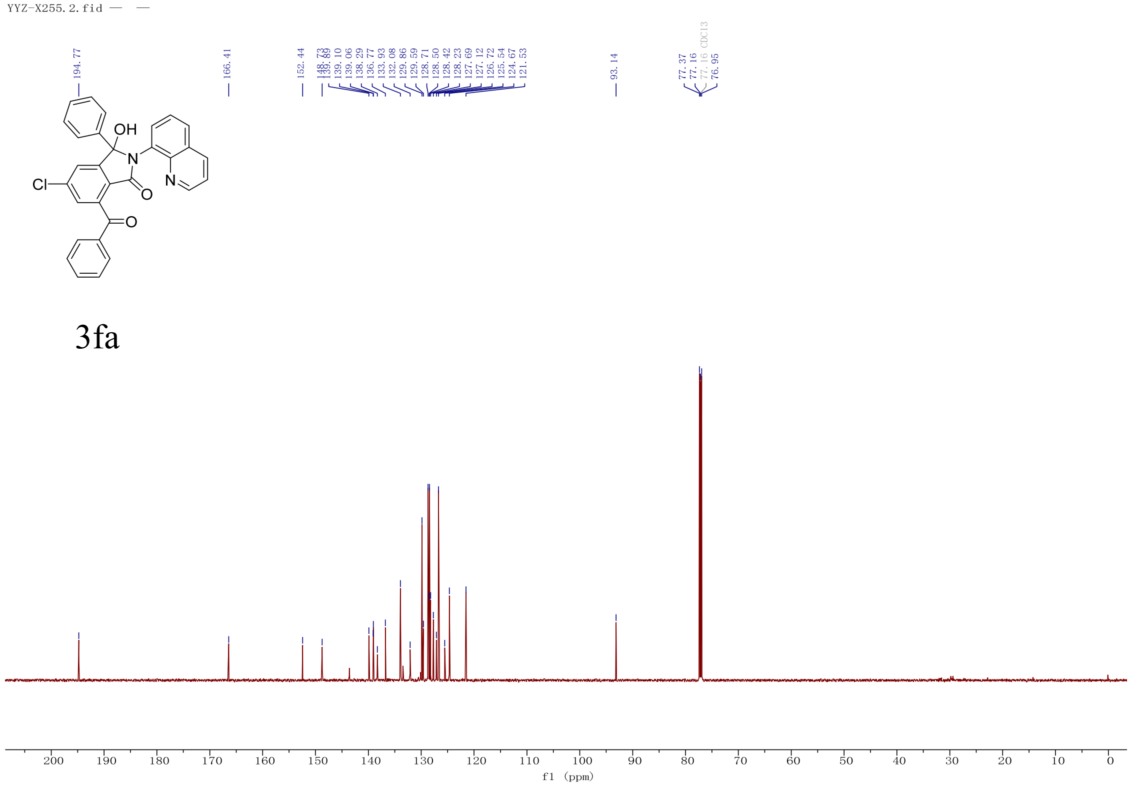

YYZ-X255.2.fid
194.77
166.41
152.44
148.73
139.89
139.10
139.06
138.29
136.77
133.93
132.08
129.86
129.59
128.71
128.50
128.42
128.23
127.69
127.12
126.72
125.54
124.67
121.53
93.14
77.37
77.16
77.16 CDCl3
76.95
OH
N
Cl
N
O
O
f1 (ppm)

3fa

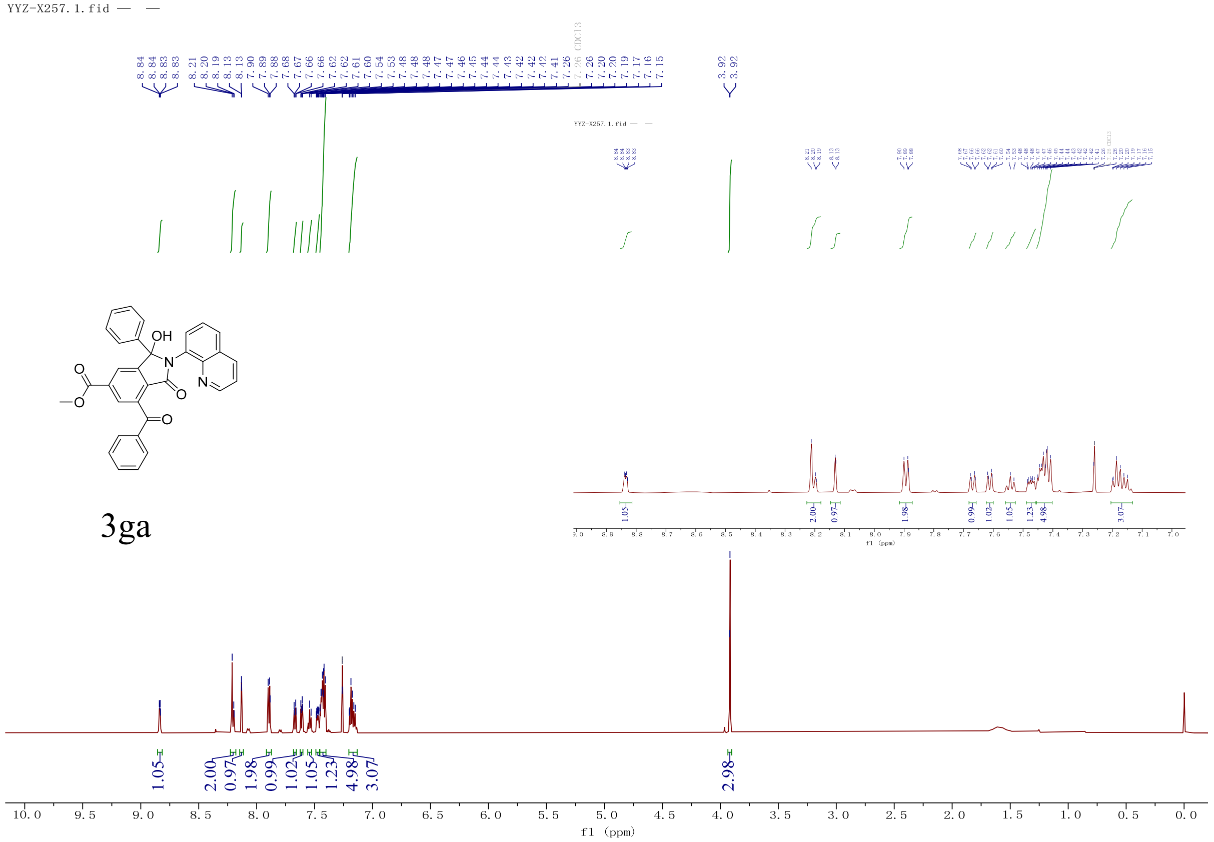
YYZ-X257.1.fid
8.84
8.84
8.83
8.83
8.21
8.20
8.19
8.13
8.13
7.90
7.89
7.88
7.68
7.67
7.66
7.66
7.62
7.62
7.61
7.60
7.54
7.53
7.48
7.48
7.47
7.47
7.46
7.45
7.44
7.43
7.42
7.42
7.42
7.41
7.26
7.26 CDCl3
7.20
7.20
7.19
7.17
7.16
7.15
3.92
3.92
1.05
2.00
0.97
1.98
0.99
1.02
1.05
1.23
4.98
3.07
2.98
f1 (ppm)
OH
N
N
O
O
O
O

3ga

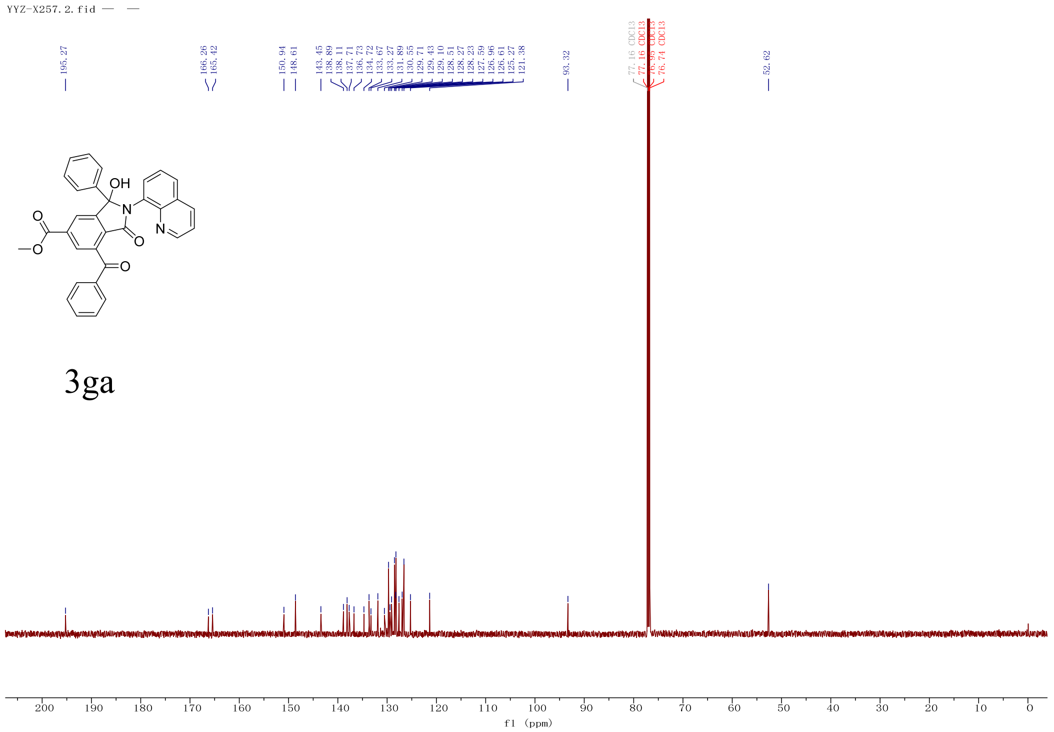
YYZ-X257.2.fid
195.27
166.26
165.42
150.94
148.61
143.45
138.89
138.11
137.71
136.73
134.72
133.67
133.27
131.89
130.55
129.71
129.43
129.10
128.51
128.27
128.23
127.59
126.96
126.61
125.27
121.38
93.32
77.16 CDCl3
77.16 CDCl3
76.95 CDCl3
76.74 CDCl3
52.62
f1 (ppm)
OH
N
N
O
O
O
O

3ga

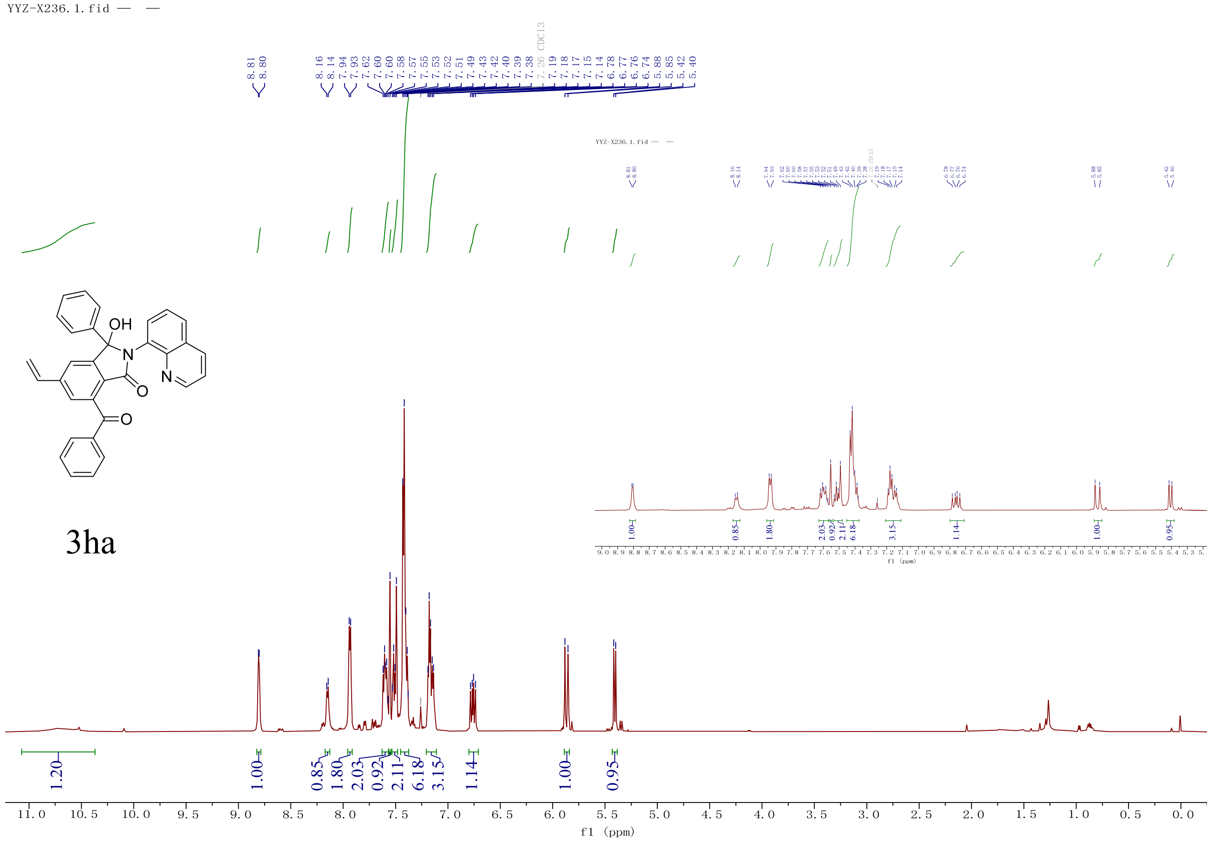
YYZ-X236.1.fid
8.81
8.80
8.16
8.14
7.94
7.93
7.62
7.60
7.60
7.58
7.57
7.55
7.53
7.52
7.51
7.49
7.43
7.42
7.40
7.39
7.38
7.26 CDCl3
7.19
7.18
7.17
7.15
7.14
6.78
6.77
6.76
6.74
5.88
5.85
5.42
5.40
OH
N
N
O
O
1.20
1.00
0.85
1.80
2.03
0.92
2.11
6.18
3.15
1.14
1.00
0.95
f1 (ppm)

3ha

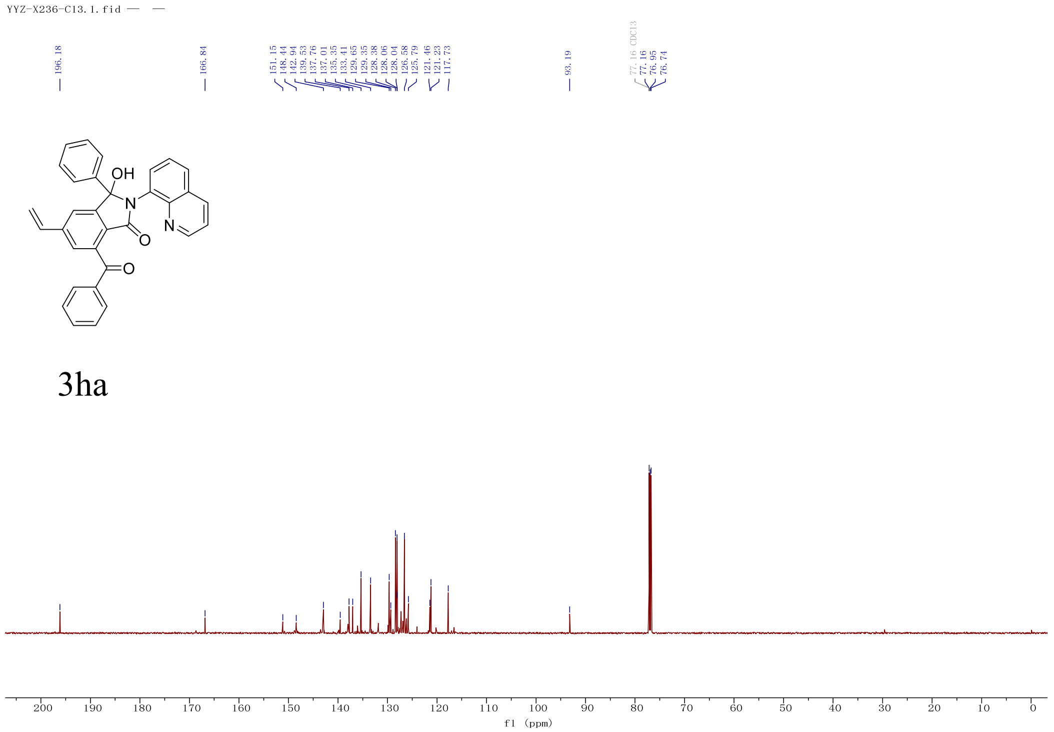
YYZ-X236-C13.1.fid
196.18
166.84
151.15
148.44
142.94
139.53
137.76
137.01
135.35
133.41
129.65
129.35
128.38
128.06
128.04
126.58
125.79
121.46
121.23
117.73
93.19
77.16 CDCl3
77.16
76.95
76.74
OH
N
N
O
O
f1 (ppm)

3ha

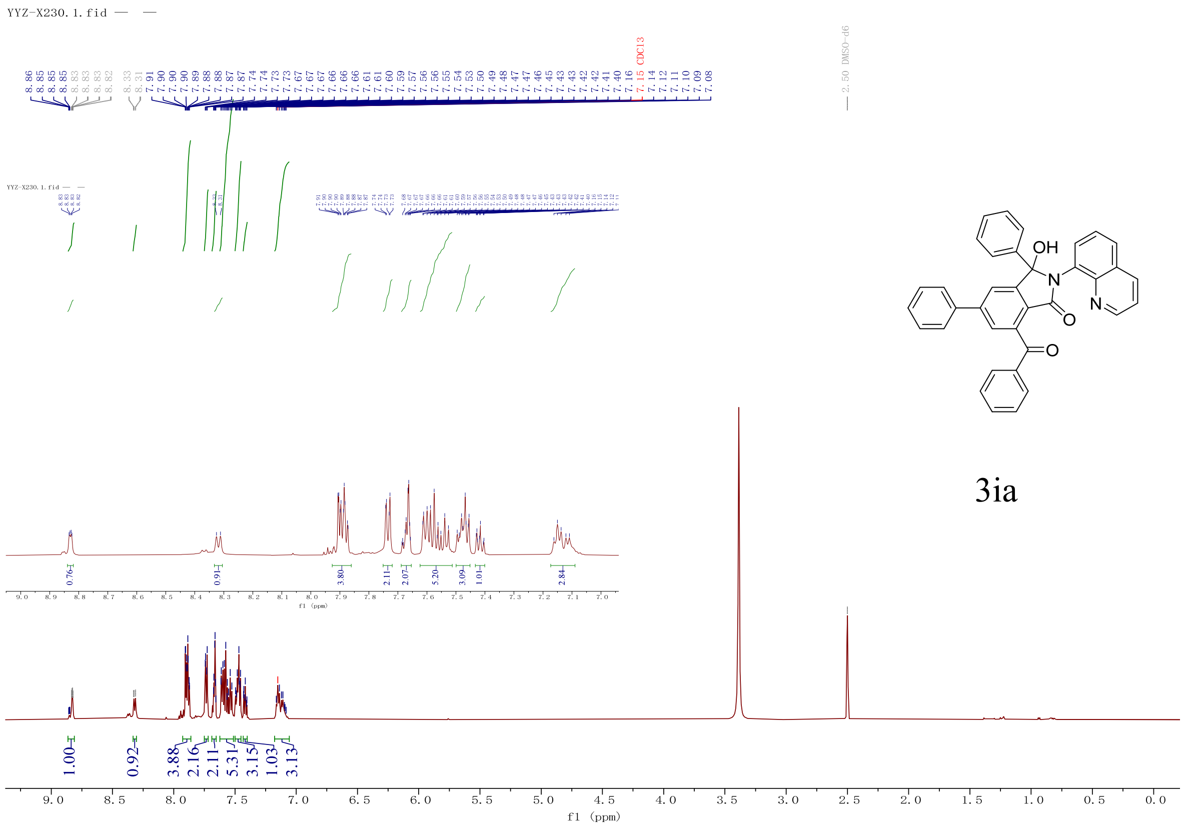
YYZ-X230.1.fid
2.50 DMSO-d6
7.15 CDCl3
OH
N
N
O
O
f1 (ppm)

3ia

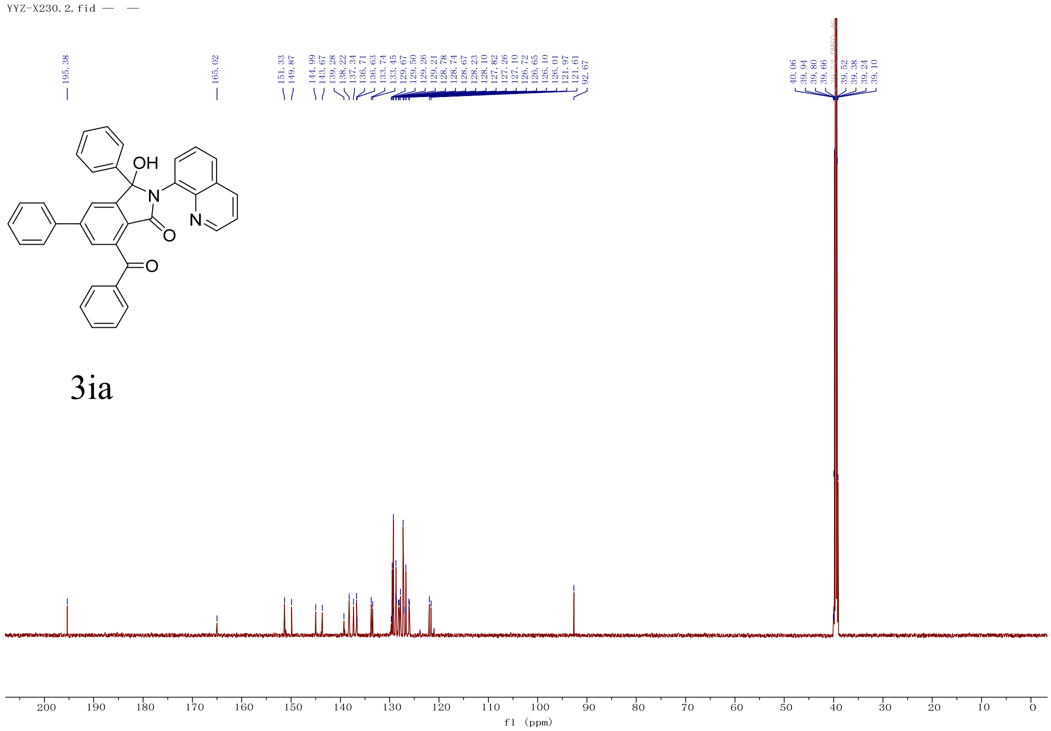
YYZ-X230.2.fid
OH
N
N
O
O
f1 (ppm)

3ia

YYZ-X286.1.fid

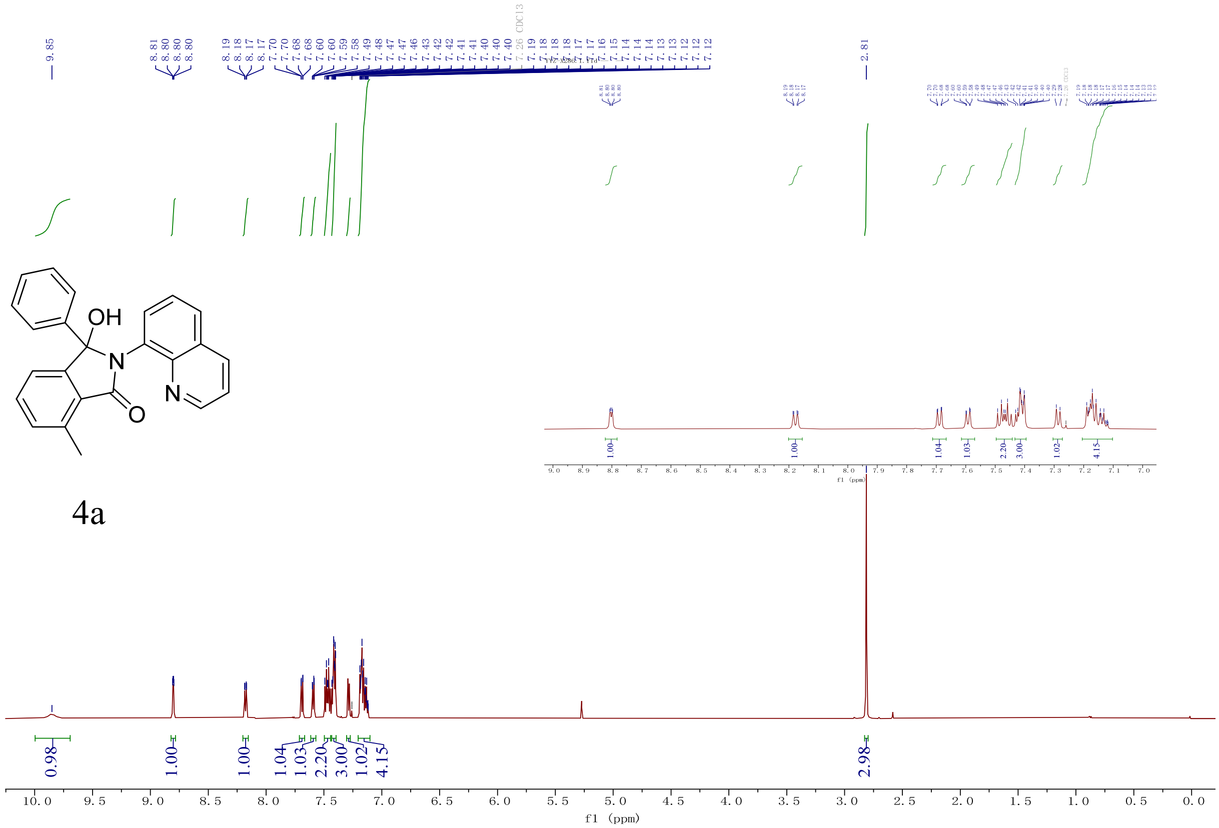

4a

YYZ-X286.2.fid

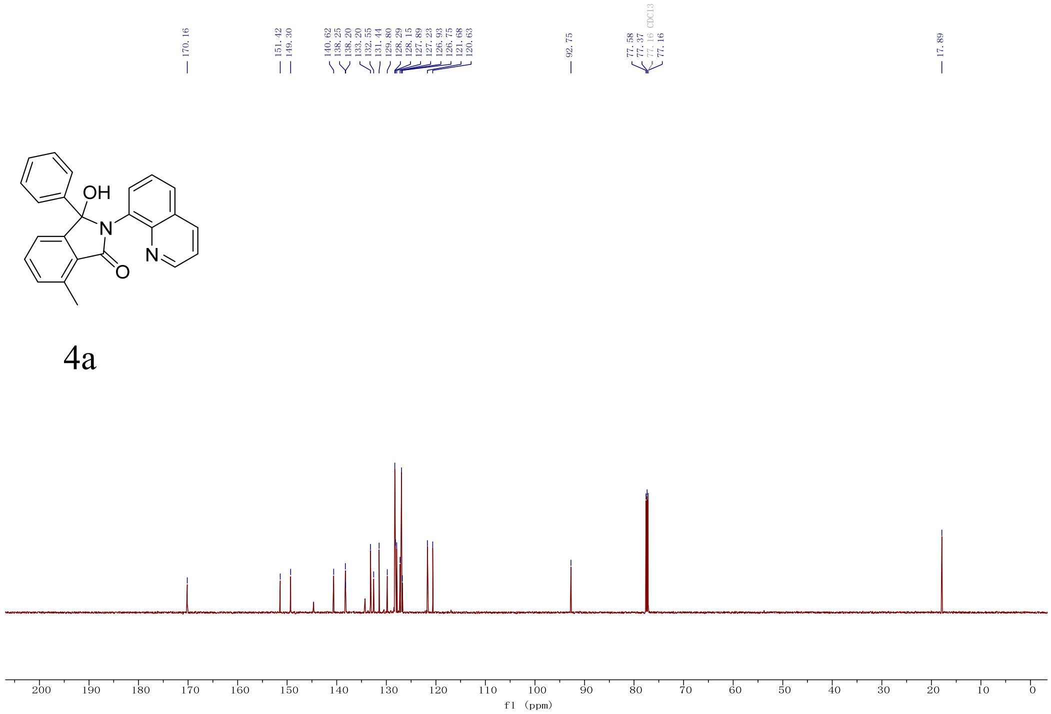

4a

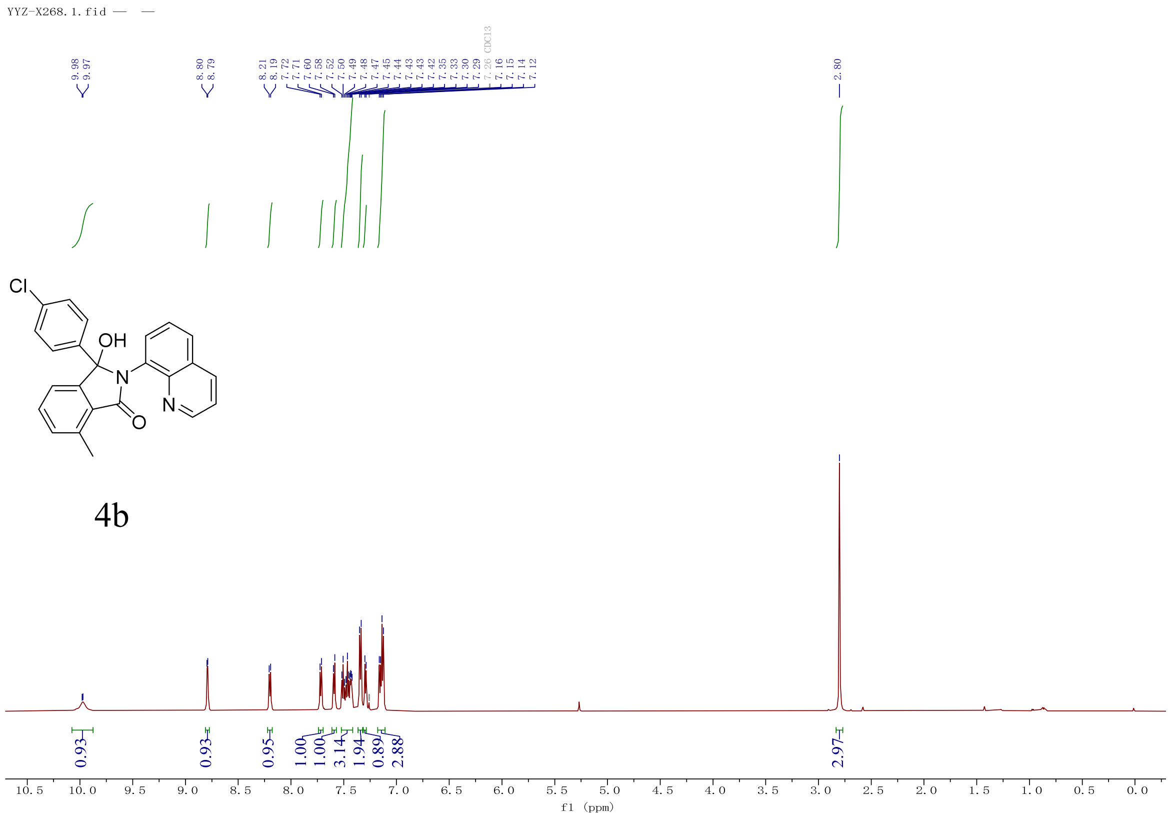
YYZ-X268.1.fid
9.98
9.97
8.80
8.79
8.21
8.19
7.72
7.71
7.60
7.58
7.52
7.50
7.49
7.48
7.47
7.45
7.44
7.43
7.43
7.42
7.35
7.33
7.30
7.29
7.26 CDCl3
7.16
7.15
7.14
7.12
2.80
Cl
OH
N
N
O
0.93
0.93
0.95
1.00
1.00
3.14
1.94
0.89
2.88
2.97
f1 (ppm)

4b

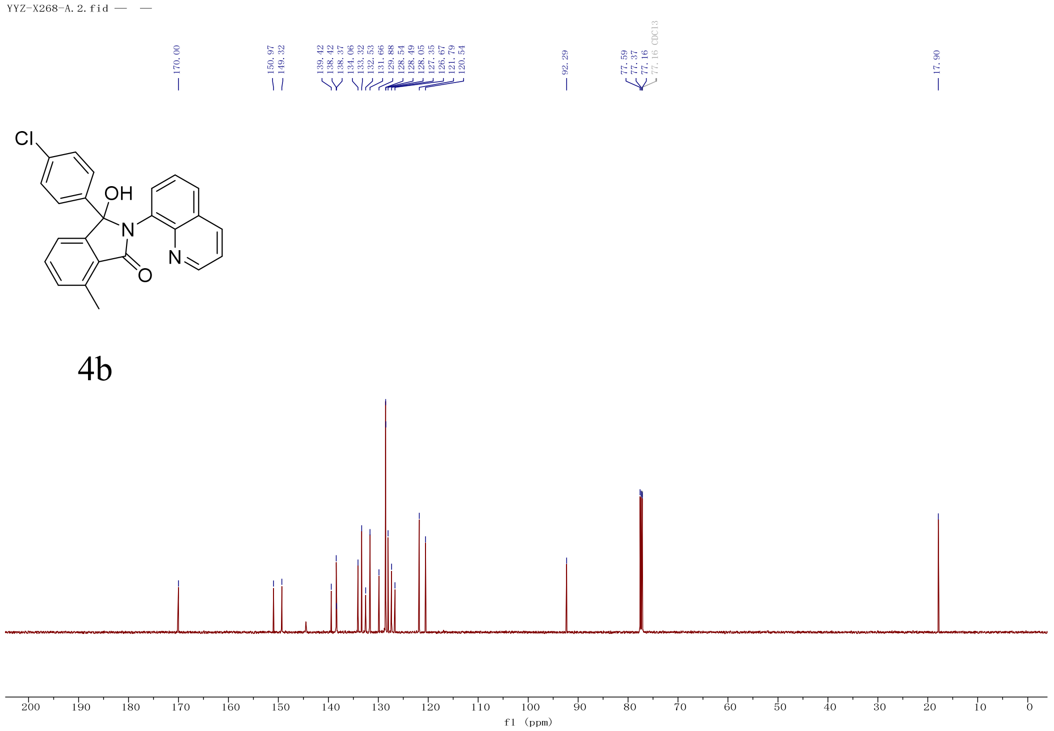
YYZ-X268-A.2.fid
170.00
150.97
149.32
139.42
138.42
138.37
134.06
133.32
132.53
131.66
129.88
128.54
128.49
128.05
127.35
126.67
121.79
120.54
92.29
77.59
77.37
77.16
77.16 CDCl3
17.90
Cl
OH
N
N
O
f1 (ppm)

4b

YYZ-X272.1.fid

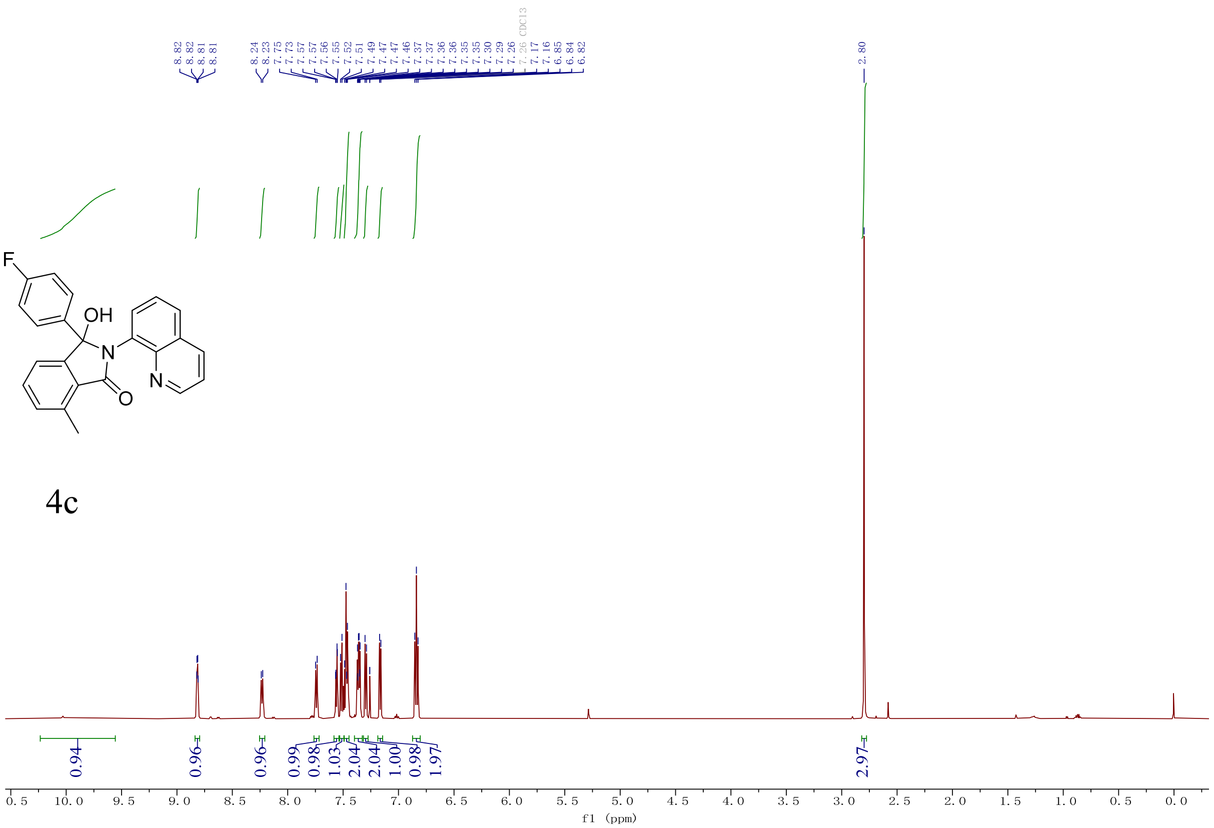

YYZ-X272.2.fid

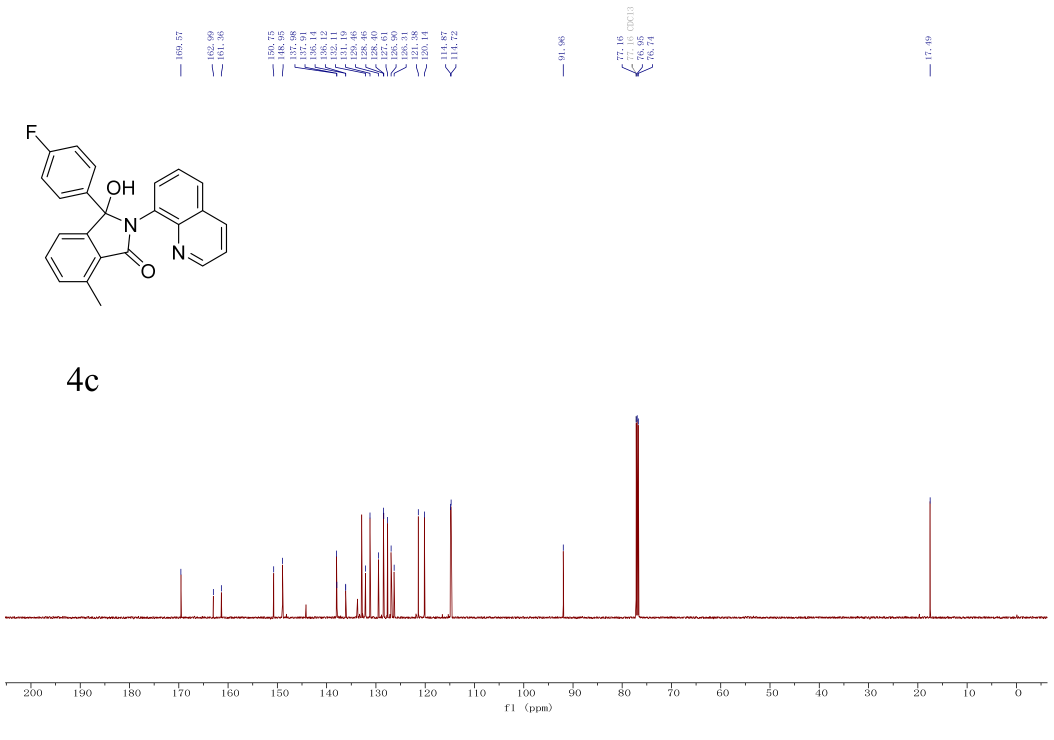

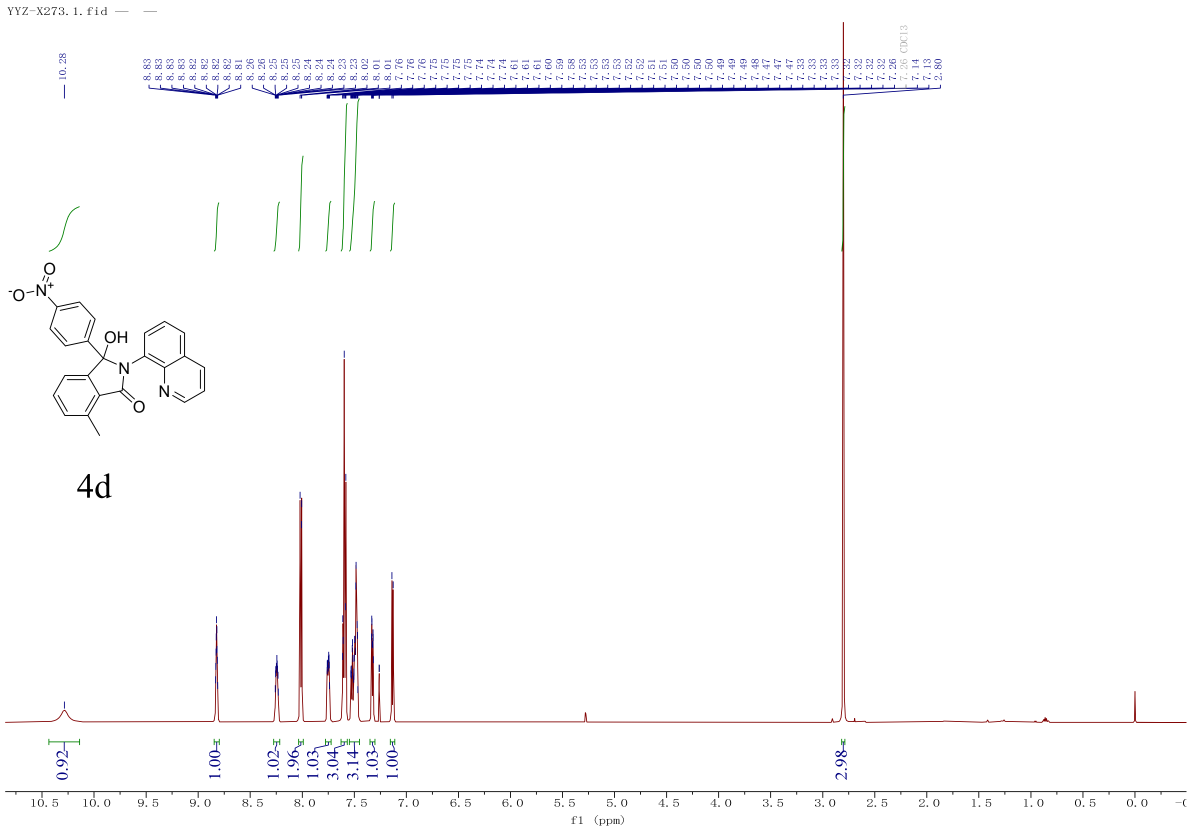
YYZ-X273.1.fid — —
10.28
8.83 8.83 8.83 8.83 8.82 8.82 8.82 8.82 8.81 8.26 8.26 8.25 8.25 8.25 8.24 8.24 8.24 8.24 8.23 8.23 8.02 8.01 8.01 7.76 7.76 7.75 7.75 7.75 7.75 7.74 7.74 7.74 7.61 7.61 7.61 7.60 7.59 7.58 7.53 7.53 7.53 7.53 7.52 7.52 7.51 7.51 7.50 7.50 7.50 7.50 7.49 7.49 7.49 7.48 7.47 7.47 7.47 7.33 7.33 7.33 7.33 7.33 7.32 7.32 7.32 7.32 7.26 7.26 CDCl3 7.14 7.13 2.80
0.92 1.00 1.02 1.96 1.03 3.04 3.14 1.03 1.00 2.98
10.5 10.0 9.5 9.0 8.5 8.0 7.5 7.0 6.5 6.0 5.5 5.0 4.5 4.0 3.5 3.0 2.5 2.0 1.5 1.0 0.5 0.0
f1 (ppm)

4d

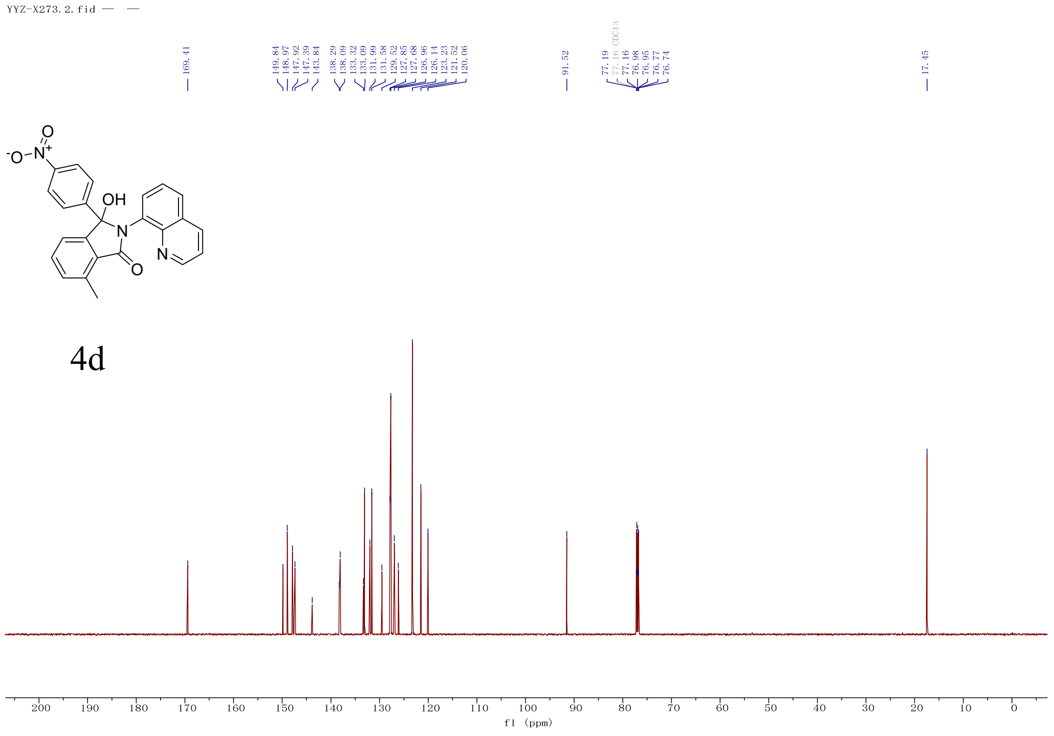
YYZ-X273.2.fid — —
169.41
149.84 148.97 147.92 147.39 143.84 138.29 138.09 133.32 133.09 131.99 131.58 129.52 127.85 127.68 126.96 126.14 123.23 121.52 120.06
91.52
77.19 77.16 CDCl3 77.16 76.98 76.95 76.77 76.74
17.45
200 190 180 170 160 150 140 130 120 110 100 90 80 70 60 50 40 30 20 10 0
f1 (ppm)

4d

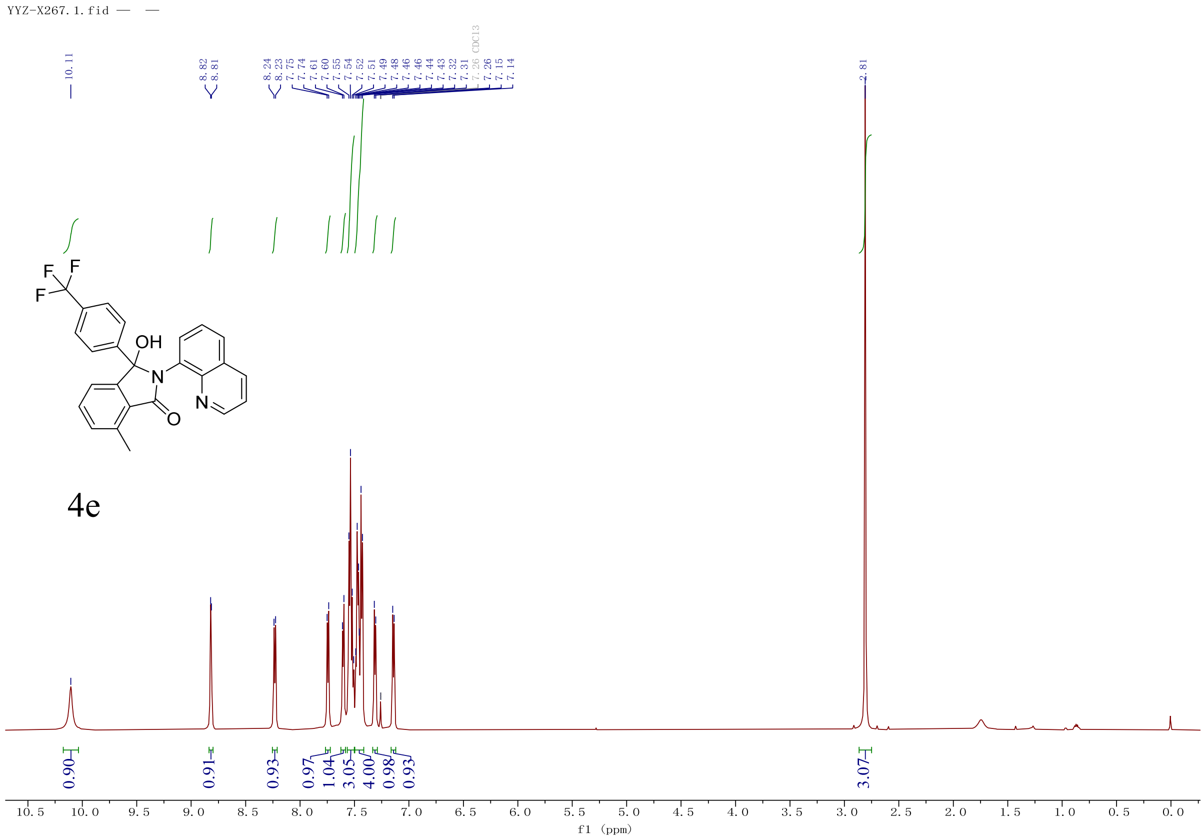

YYZ-X267.1.fid
10.11
8.82
8.81
8.24
8.23
7.75
7.74
7.61
7.60
7.55
7.54
7.52
7.51
7.49
7.48
7.46
7.46
7.44
7.43
7.32
7.31
7.26 CDCl3
7.26
7.15
7.14
2.81
OH
N
N
O
F
F
F
0.90
0.91
0.93
0.97
1.04
3.05
4.00
0.98
0.93
3.07
10.5 10.0 9.5 9.0 8.5 8.0 7.5 7.0 6.5 6.0 5.5 5.0 4.5 4.0 3.5 3.0 2.5 2.0 1.5 1.0 0.5 0.0
f1 (ppm)

4e

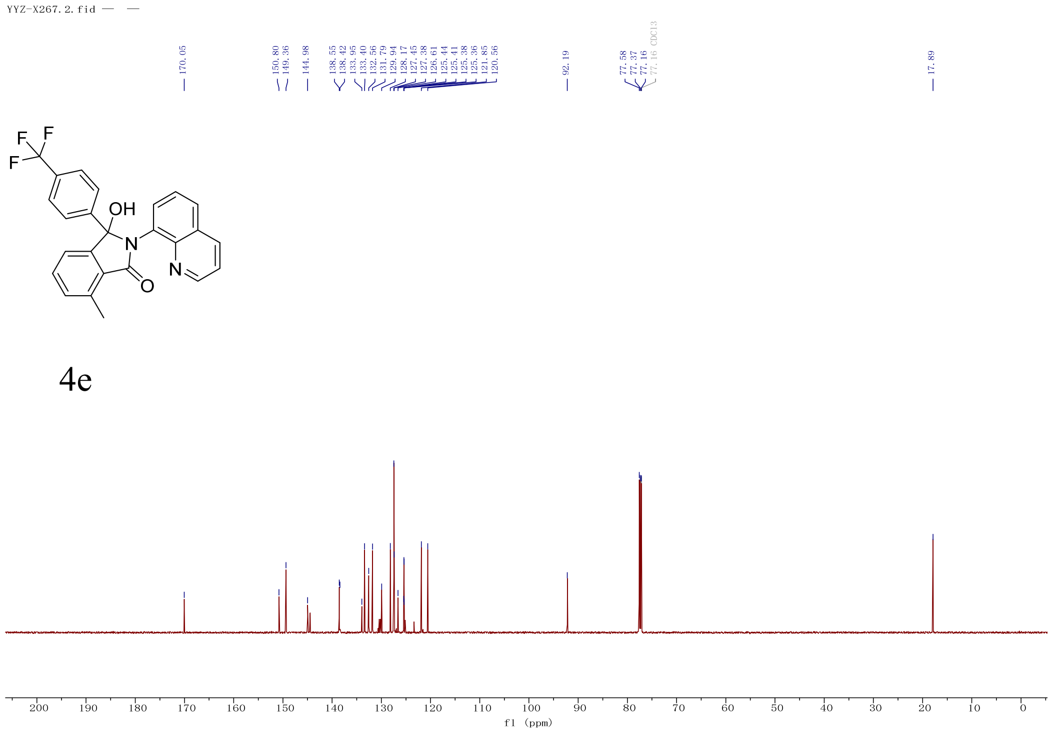

YYZ-X267.2.fid
170.05
150.80
149.36
144.98
138.55
138.42
133.95
133.40
132.56
131.79
129.94
128.17
127.45
127.38
126.61
125.44
125.41
125.38
125.36
121.85
120.56
92.19
77.58
77.37
77.16 CDCl3
17.89
OH
N
N
O
F
F
F
200 190 180 170 160 150 140 130 120 110 100 90 80 70 60 50 40 30 20 10 0
f1 (ppm)

4e

YYZ-X264.1.fid

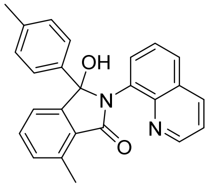

4f

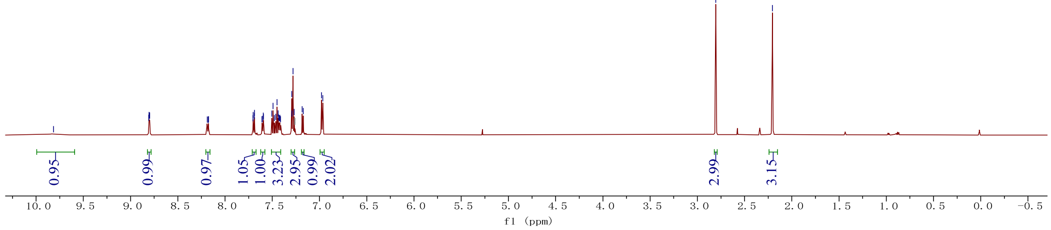

YYZ-X264.2.fid

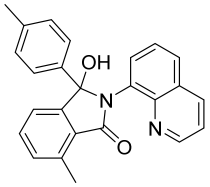

4f

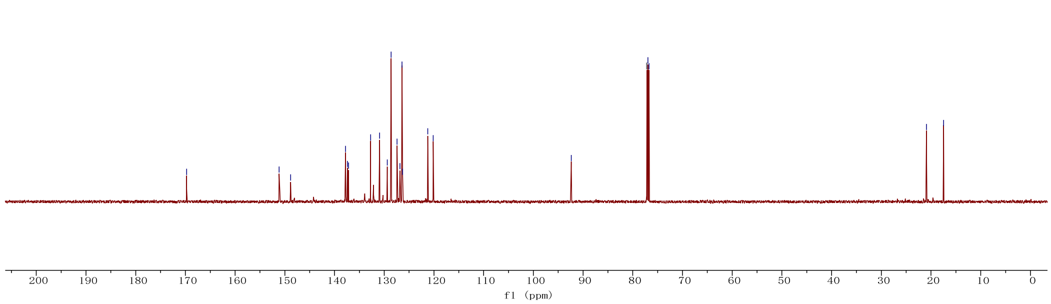

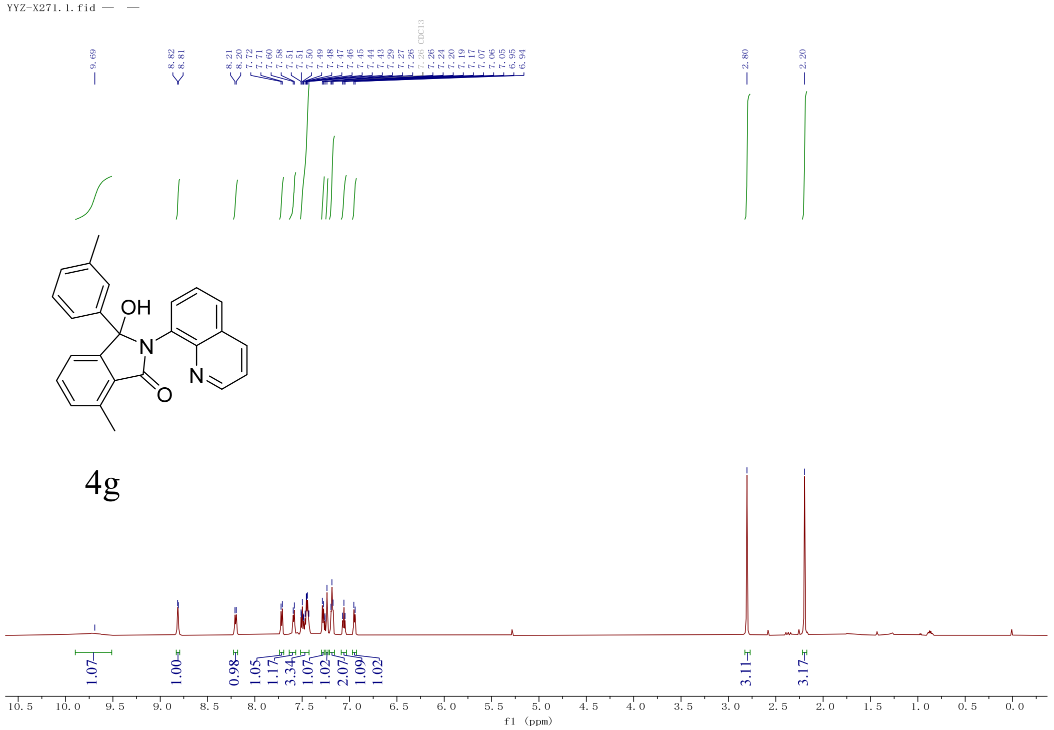
YYZ-X271.1.fid
9.69
8.82
8.81
8.21
8.20
7.72
7.71
7.60
7.58
7.51
7.51
7.50
7.49
7.48
7.47
7.46
7.45
7.44
7.43
7.29
7.27
7.26 CDCl3
7.26
7.24
7.20
7.19
7.17
7.07
7.06
7.05
6.95
6.94
2.80
2.20
OH
N
N
O
4g
1.07
1.00
0.98
1.05
1.17
3.34
1.07
1.02
2.07
1.09
1.02
3.11
3.17
10.5
10.0
9.5
9.0
8.5
8.0
7.5
7.0
6.5
6.0
5.5
5.0
4.5
4.0
3.5
3.0
2.5
2.0
1.5
1.0
0.5
0.0
f1 (ppm)

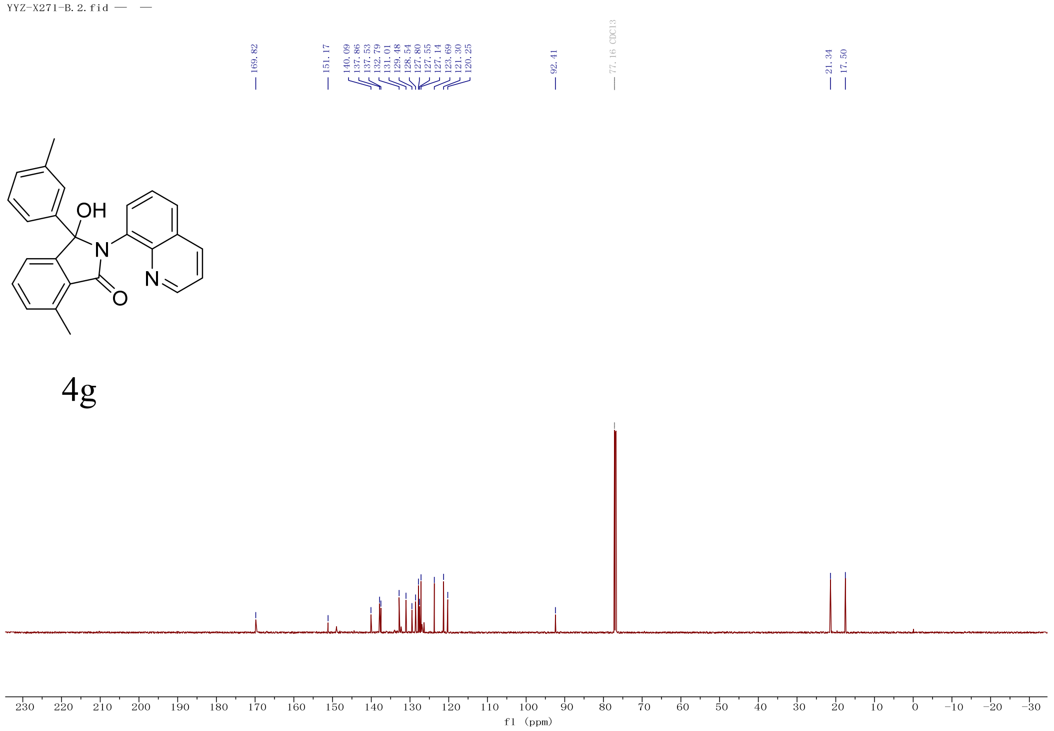
YYZ-X271-B.2.fid
169.82
151.17
140.09
137.86
137.53
132.79
131.01
129.48
128.54
127.80
127.55
127.14
123.69
121.30
120.25
92.41
77.16 CDCl3
21.34
17.50
OH
N
N
O
4g
230
220
210
200
190
180
170
160
150
140
130
120
110
100
90
80
70
60
50
40
30
20
10
0
-10
-20
-30
f1 (ppm)

YYZ-X270-B.1.fid — —

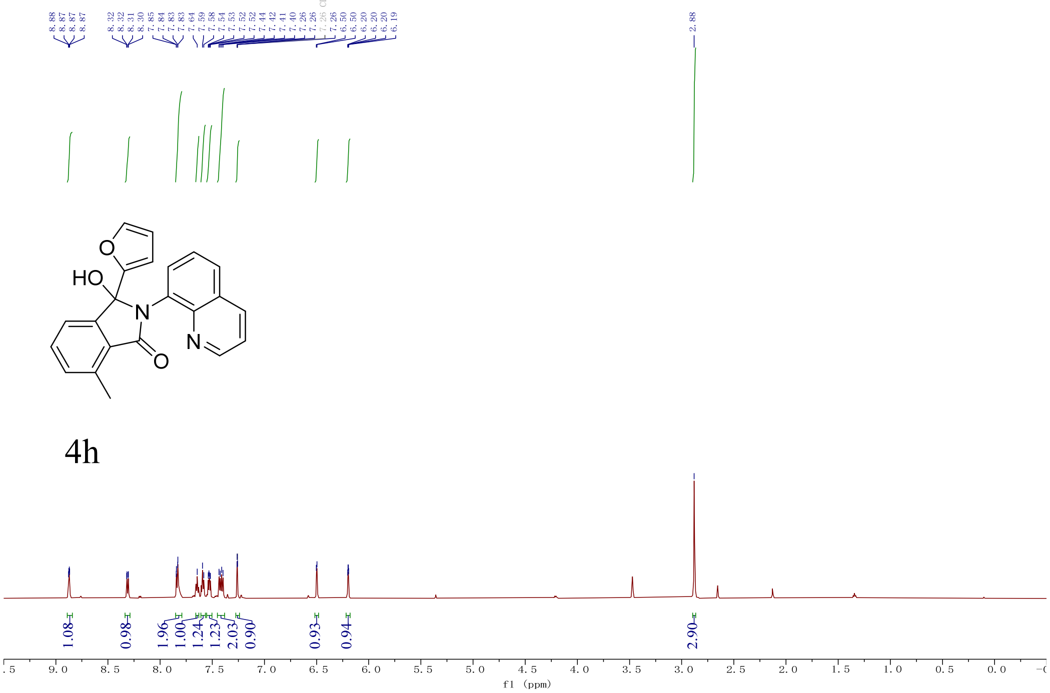

4h

YYZ-X270-B.2.fid — —

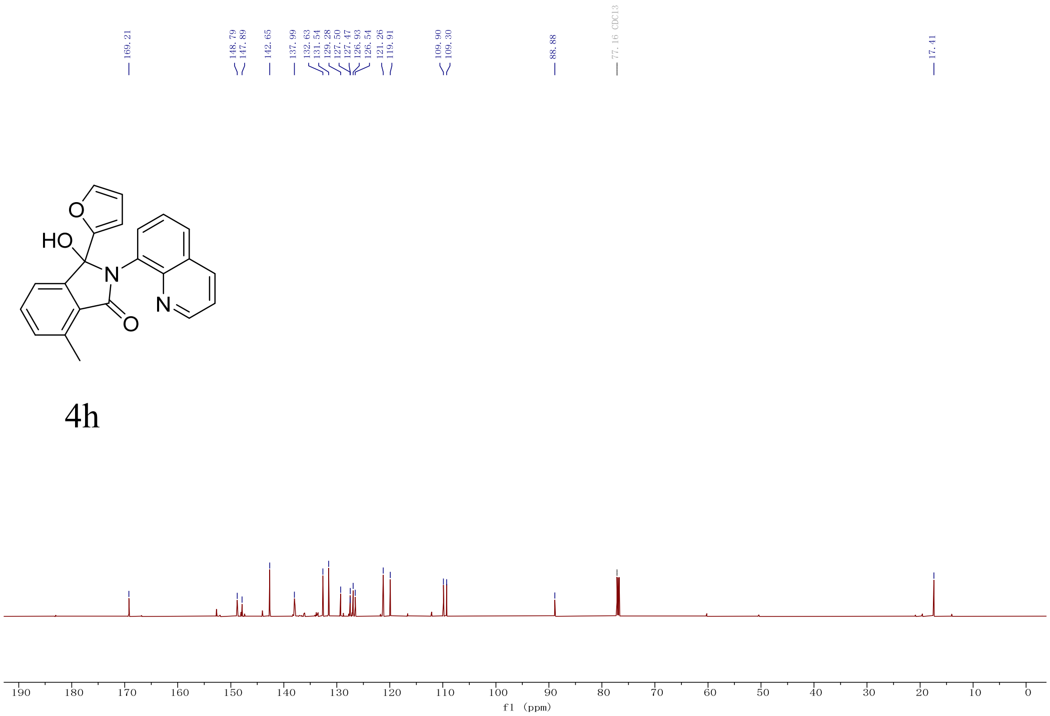

4h

3. Single-crystal information (3aa and 4a)

Single-crystal information (**3aa**):

The crystal data were collected on a Agilent Gemini E diffractometer （Mo，50kV 40mA）and reducted by CrysAlisPro (Rigaku). The structures were solved by direct methods using SHELXS-97. Refinements were performed with SHELXL-2013 using fullmatrix least-squares calculations on F2, with anisotropic displacement parameters for all the nonhydrogen atoms.

Description of sample preparation: 20 mg of compound 3aa was dissolved in 1 mL of DCMl. The vial was capped and stayed untouched for a couple of days until the formation of crystals for analysis.

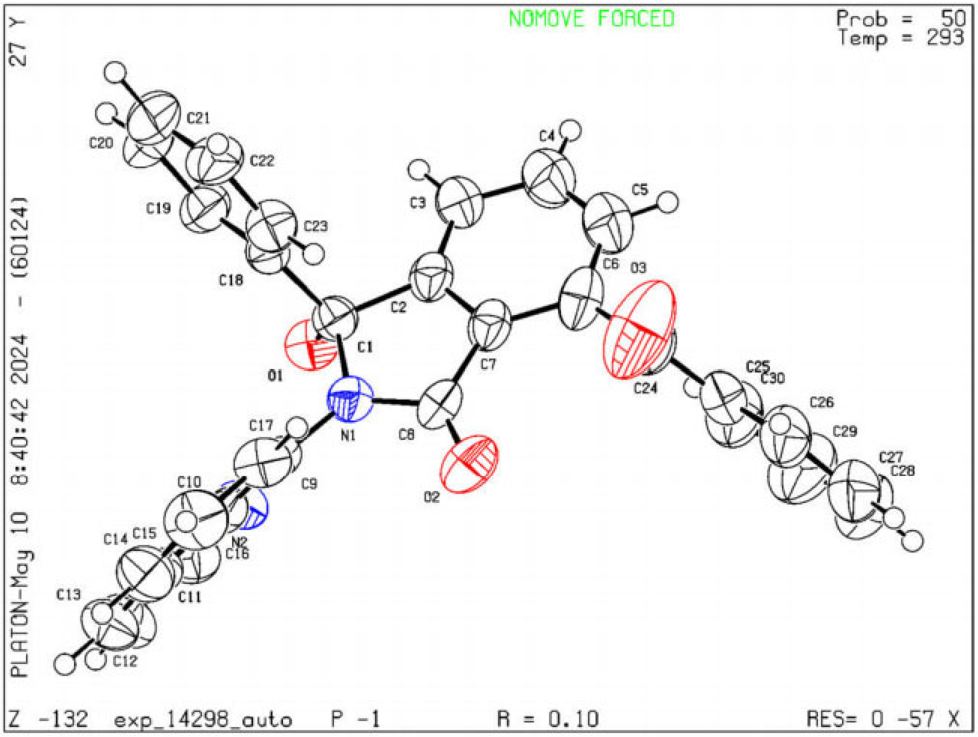

50% ellipsoidal ratio

exp_14298_auto

Table 1 Crystal data and structure refinement for exp_14298_auto.

Identification code	exp_14298_auto
Empirical formula	$C_{30}H_{19}N_2O_3$
Formula weight	455.47
Temperature/K	293(2)
Crystal system	triclinic
Space group	P-1
a/Å	8.3011(6)
b/Å	9.4240(10)
c/Å	17.4304(19)
α/°	90.120(9)
β/°	96.716(7)
γ/°	95.755(7)
Volume/Å^3	1347.3(2)
Z	2
ρ_{calc}g/cm^3	1.123
μ/mm^{-1}	0.073
F(000)	474.0
Crystal size/mm^3	0.15 × 0.12 × 0.08
Radiation	Mo Kα (λ = 0.71073)
2Θ range for data collection/°	6.26 to 49.992
Index ranges	-9 ≤ h ≤ 9, -11 ≤ k ≤ 10, -20 ≤ l ≤ 19
Reflections collected	9256
Independent reflections	4640 [R_{int} = 0.0417, R_{sigma} = 0.0929]
Data/restraints/parameters	4640/0/316
Goodness-of-fit on F^2	1.011
Final R indexes [I>=2σ (I)]	R_1 = 0.0998, wR_2 = 0.2259
Final R indexes [all data]	R_1 = 0.1580, wR_2 = 0.2678
Largest diff. peak/hole / e Å^{-3}	0.48/-0.32

Single-crystal information (**4a**):

The crystal data were collected on a Agilent Gemini E diffractometer （Mo，50kV 40mA）and reducted by CrysAlisPro (Rigaku). The structures were solved by direct methods using SHELXS-97. Refinements were performed with SHELXL-2013 using fullmatrix least-squares calculations on F2, with anisotropic displacement parameters for all the nonhydrogen atoms.

Description of sample preparation: 20 mg of compound 3aa was dissolved in 1 mL of DCMl. The vial was capped and stayed untouched for a couple of days until the formation of crystals for analysis.

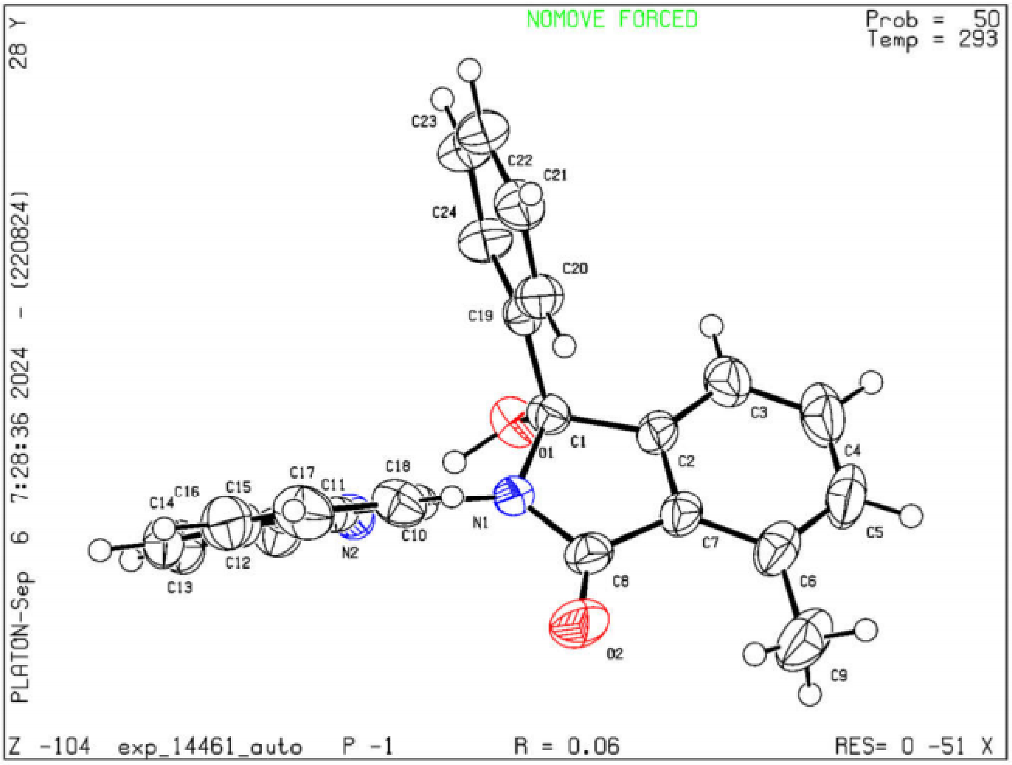

50% ellipsoidal ratio

exp_14461_auto

Table 1 Crystal data and structure refinement for exp_14461_auto.

Identification code	exp_14461_auto
Empirical formula	$C_{24}H_{18}N_2O_2$
Formula weight	366.40
Temperature/K	293(2)
Crystal system	triclinic
Space group	P-1
a/Å	8.3744(11)
b/Å	9.0919(9)
c/Å	12.6364(19)
α/°	99.343(10)
β/°	105.567(12)
γ/°	93.630(9)
Volume/Å^3	908.7(2)
Z	2
ρ_{calc}g/cm^3	1.339
μ/mm^{-1}	0.086
F(000)	384.0
Crystal size/mm^3	0.18 × 0.12 × 0.11
Radiation	Mo Kα (λ = 0.71073)
2Θ range for data collection/°	4.57 to 52
Index ranges	-10 ≤ h ≤ 7, -11 ≤ k ≤ 11, -15 ≤ l ≤ 15
Reflections collected	6836
Independent reflections	3576 [R_{int} = 0.0346, R_{sigma} = 0.0742]
Data/restraints/parameters	3576/0/258
Goodness-of-fit on F^2	1.055
Final R indexes [I>=2σ (I)]	R_1 = 0.0622, wR_2 = 0.1018
Final R indexes [all data]	R_1 = 0.1162, wR_2 = 0.1231
Largest diff. peak/hole / e Å^{-3}	0.16/-0.19
